# Supplementary material for: Recombinant Dabie bandavirus as a bivalent vaccine platform inducing protective immunity against intracellular pathogens and cancer
Source: Mol Ther. 2025 Oct 24;34(2):1084–103. doi: 10.1016/j.ymthe.2025.10.044 (PMC12882354; doi:10.1016/j.ymthe.2025.10.044)
Supplement: Document S1. Figures S1–S10 and Table S1 [file mmc1.pdf]

YMTHE, Volume *34*

Supplemental Information

Recombinant *Dabie bandavirus* as a bivalent vaccine platform inducing protective immunity against intracellular pathogens and cancer

Hyo-Jin Ro, Yebeen Lee, Kyeongseok Jeon, Yujin Kim, Seung Ho Baek, Green Kim, Joowan Kim, Jun-Gu Kang, Na-Yoon Jang, Si-Hyeon Lee, Sun-Young Kim, Yu-Jin Kim, Na-Young Ha, Yuri Kim, Young Ki Choi, Jae U. Jung, Jung Joo Hong, and Nam-Hyuk Cho

Table S1. Serum biochemistry in WT mice following intramuscular inoculation with SFTSV-ΔNSs or mock control

Sex	Male						Female					
D.P.I.[a]	2		17		31		2		17		31	
Group (n)	Mock (5)	SFTSV-ΔNSs (5)	Mock (9)	SFTSV-ΔNSs (10)	Mock (5)	SFTSV-ΔNSs (5)	Mock (5)	SFTSV-ΔNSs (5)	Mock (9)	SFTSV-ΔNSs (10)	Mock (5)	SFTSV-ΔNSs (5)
ALT (U/L)	33.7±17.0	26.6±6.5	29.0±5.8	27.4±4.4	28.0±7.5	30.7±3.6	32.0±16.1	27.4±6.0	30.3±5.6	31.7±9.7	44.2±16.0	40.7±14.4
AST (U/L)	63.2±22.2	54.4±12.8	56.6±11.4	60.1±26.0	45.4±4.5	46.7±8.4	55.4±16.5	54.6±9.5	81.2±19.0	94.5±33.4	129.3±24.8	185.7±49.1
ALP (U/L)	535.4±72.3	528.6±50.6	392.9±43.6	387.2±40.9	336.5±30.0	316.4±21.6	540.2±83.8	584.1±93.2	590.0±60.7	492.8±59.6**	425.7±22.8	419.1±40.7
GGT (U/L)	0.02±0.03	0.17±0.29	0.59±0.25	0.55±0.33	0.00±0.00	0.00±0.00	0.23±0.27	0.26±0.19	0.15±0.22	0.08±0.12	0.00±0.00	0.02±0.05
BUN (mg/dL)	23.4±2.8	20.7±4.4	27.4±2.9	24.6±3.8	24.0±1.7	25.1±1.4	19.2±2.0	19.8±1.7	24.6±3.3	27.4±2.9	28.5±2.9	27.2±2.0
Urea (mg/dL)	50±6	44±9	59±6	53±8	51±4	54±3	41±4	42±4	53±7	59±6	61±6	59±4
Crea (mg/dL)	0.36±0.02	0.33±0.02*	0.39±0.03	0.39±0.04	0.39±0.05	0.39±0.03	0.35±0.03	0.29±0.03*	0.31±0.03	0.32±0.05	0.36±0.07	0.37±0.05

[a]D.P.I: days post-inoculation. *, $p<0.05$ by Mann Whitney test.

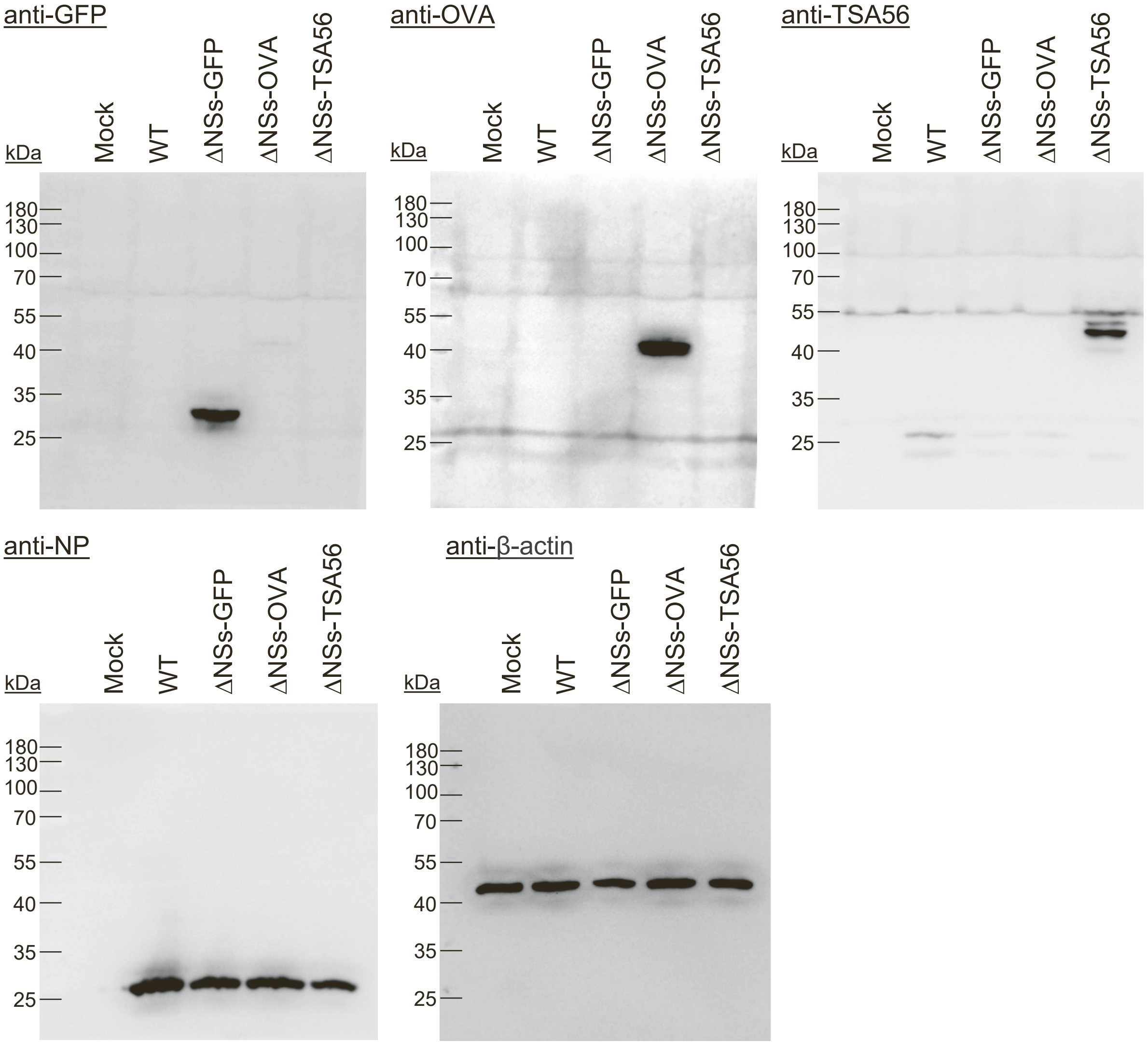

Figure S1. Immunoblot analysis of Vero E6 cells infected with SFTSV ΔNSs vectors expressing GFP, OVA, or TSA56 for 48 hours. Whole-membrane blots probed with the indicated antibodies are shown.

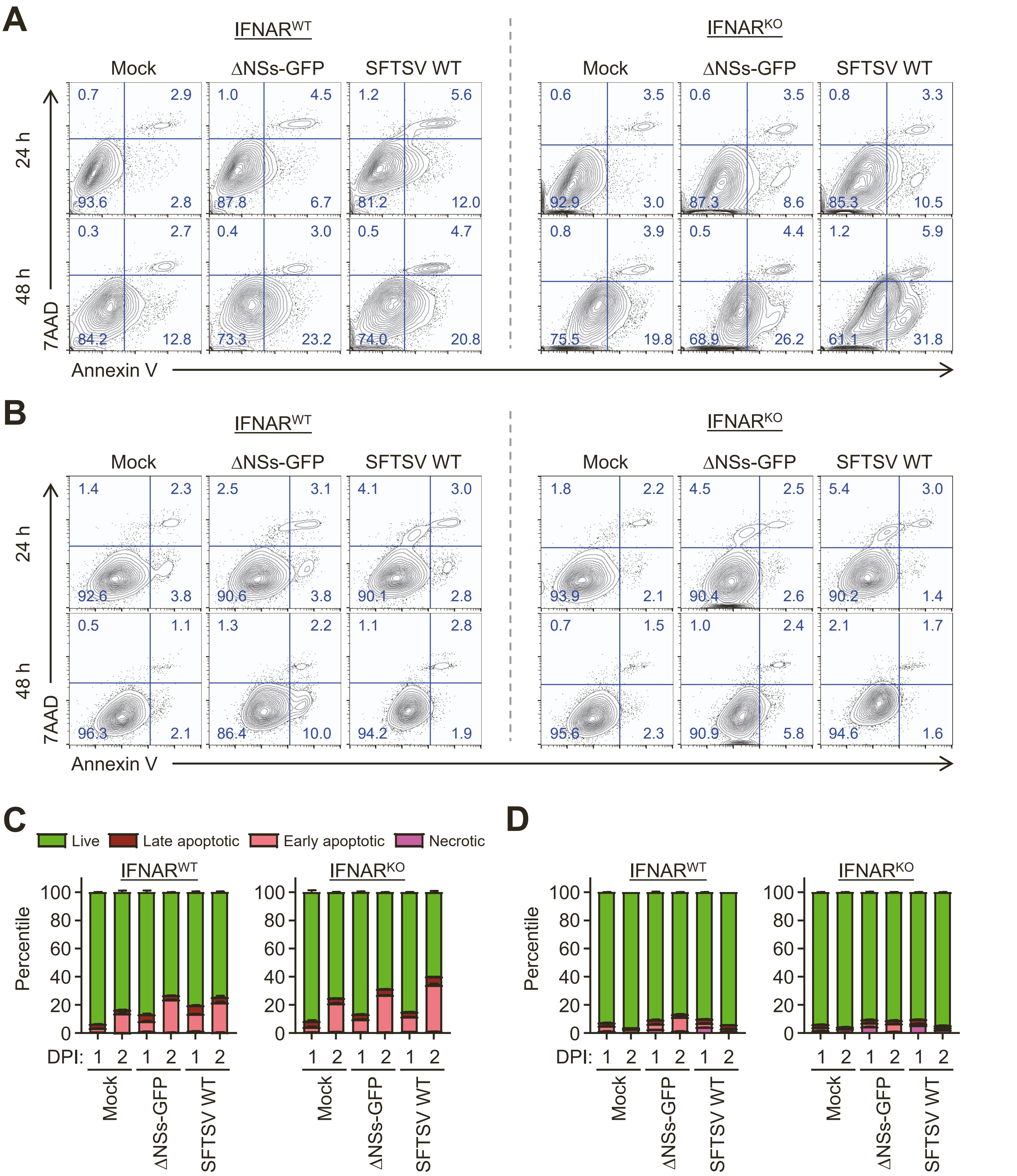

Figure S2. Apoptotic and necrotic cell death in SFTSV-infected APCs. BMDCs and BMDMs from WT or IFNAR KO mice were infected with SFTSV ΔNSs-GFP at an MOI of 1. Apoptotic and necrotic cell populations were analyzed by flow cytometry at 24 and 48 hours post-infection. **(A, B)** Representative flow cytometry plots showing Annexin V and 7-AAD staining in BMDCs **(A)** and BMDMs **(B)**. **(C, D)** Quantification of apoptotic and necrotic cell fractions in BMDCs **(C)** and BMDMs **(D)**. Cells were categorized as live (Annexin V^{-} 7-AAD^{-}), late apoptotic (Annexin V^{+} 7-AAD^{+}), early apoptotic (Annexin V^{+} 7-AAD^{-}), necrotic (Annexin V^{-} 7-AAD^{+}).

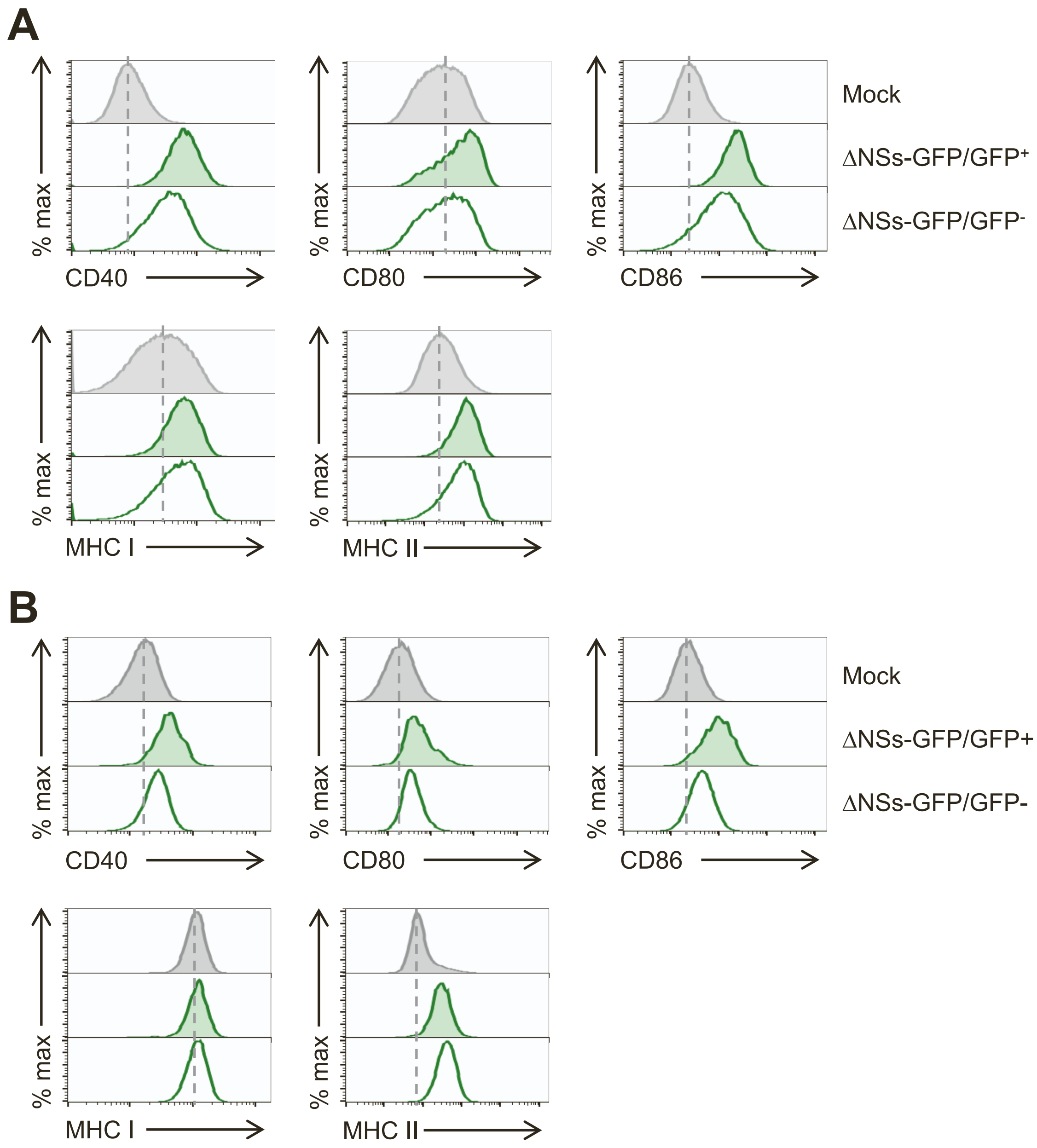

Figure S3. Histogram of co-stimulatory and MHC expression in GFP⁺ and GFP⁻ APCs. (A, B) WT BMDCs **(A)** and BMDMs **(B)** were infected with SFTSV ΔNSs-GFP at an MOI of 1. Representative histograms show the expression profiles of co-stimulatory molecules (CD40, CD80, CD86) and MHC class I and II in GFP⁺ (infected) versus GFP⁻ (uninfected) cell populations.

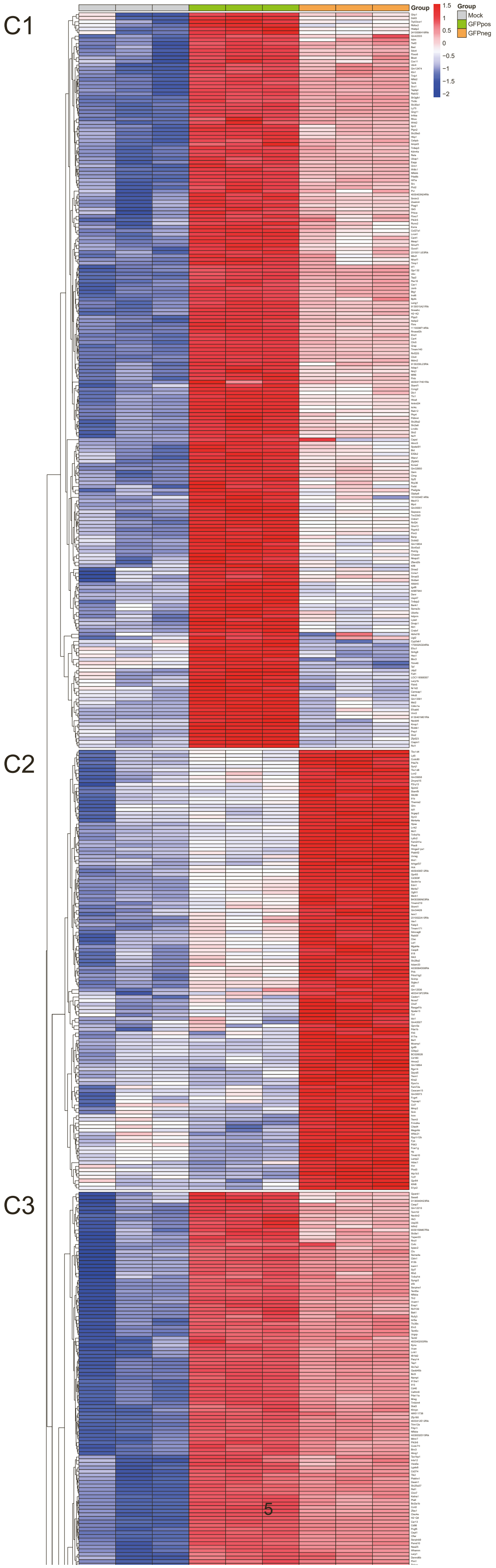
C1
C2
C3
Group
Mock
GFPpos
GFPneg
1.5
1
0.5
0
−0.5
−1
−1.5
−2

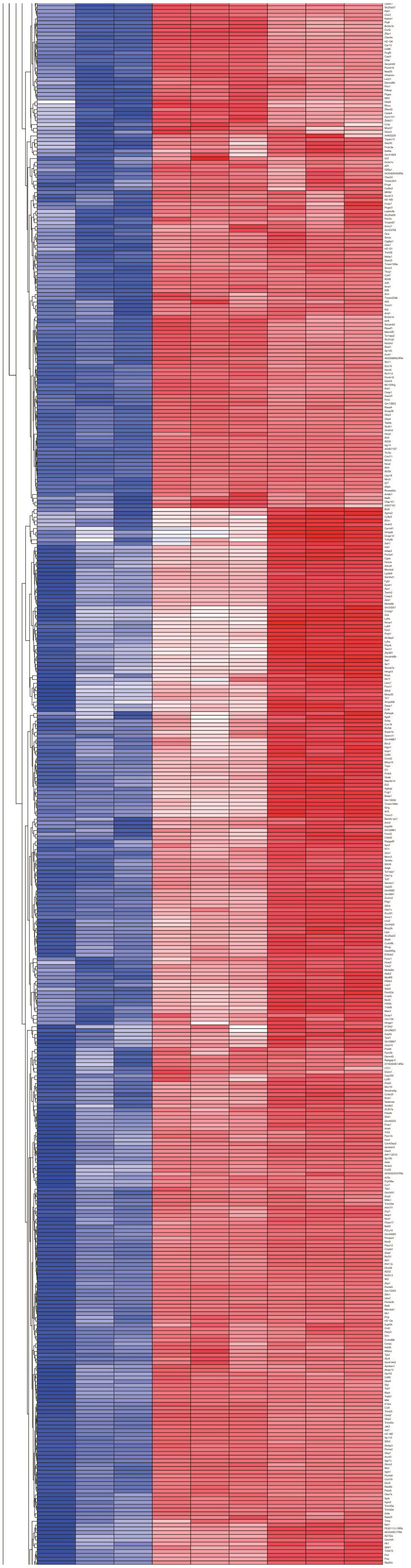

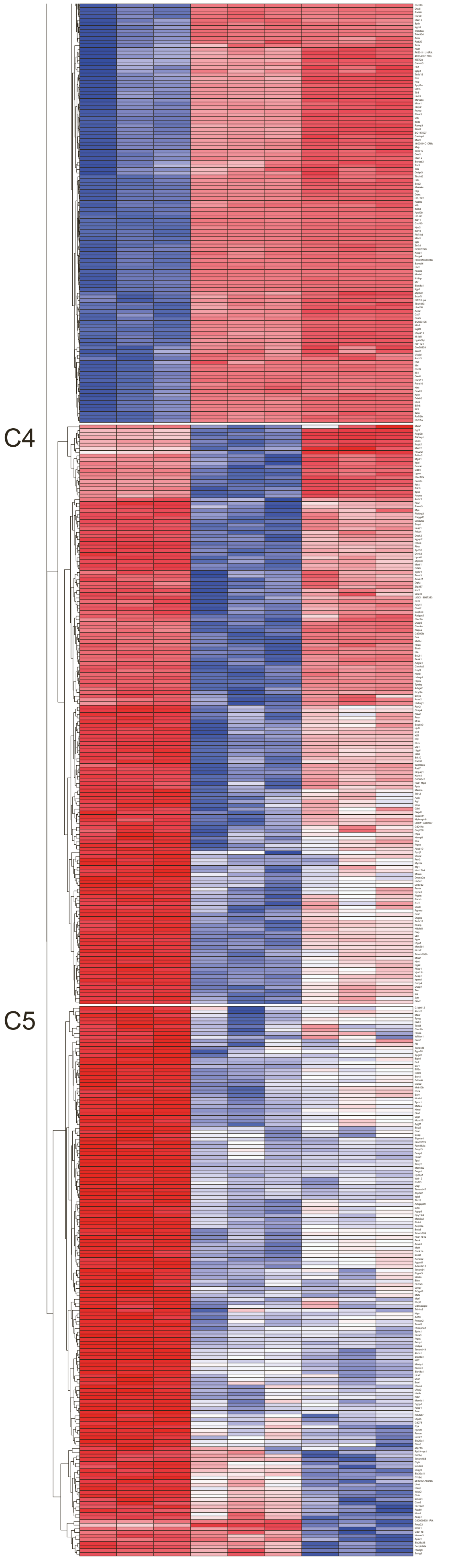

Figure S4. Heatmap of differentially expressed genes (DEGs) across groups. Genes with |Log2 (fold change, FC)| > 1 and FDR < 0.05 (relative to mock) were clustered into five modules (C1–C5) by unsupervised hierarchical clustering.

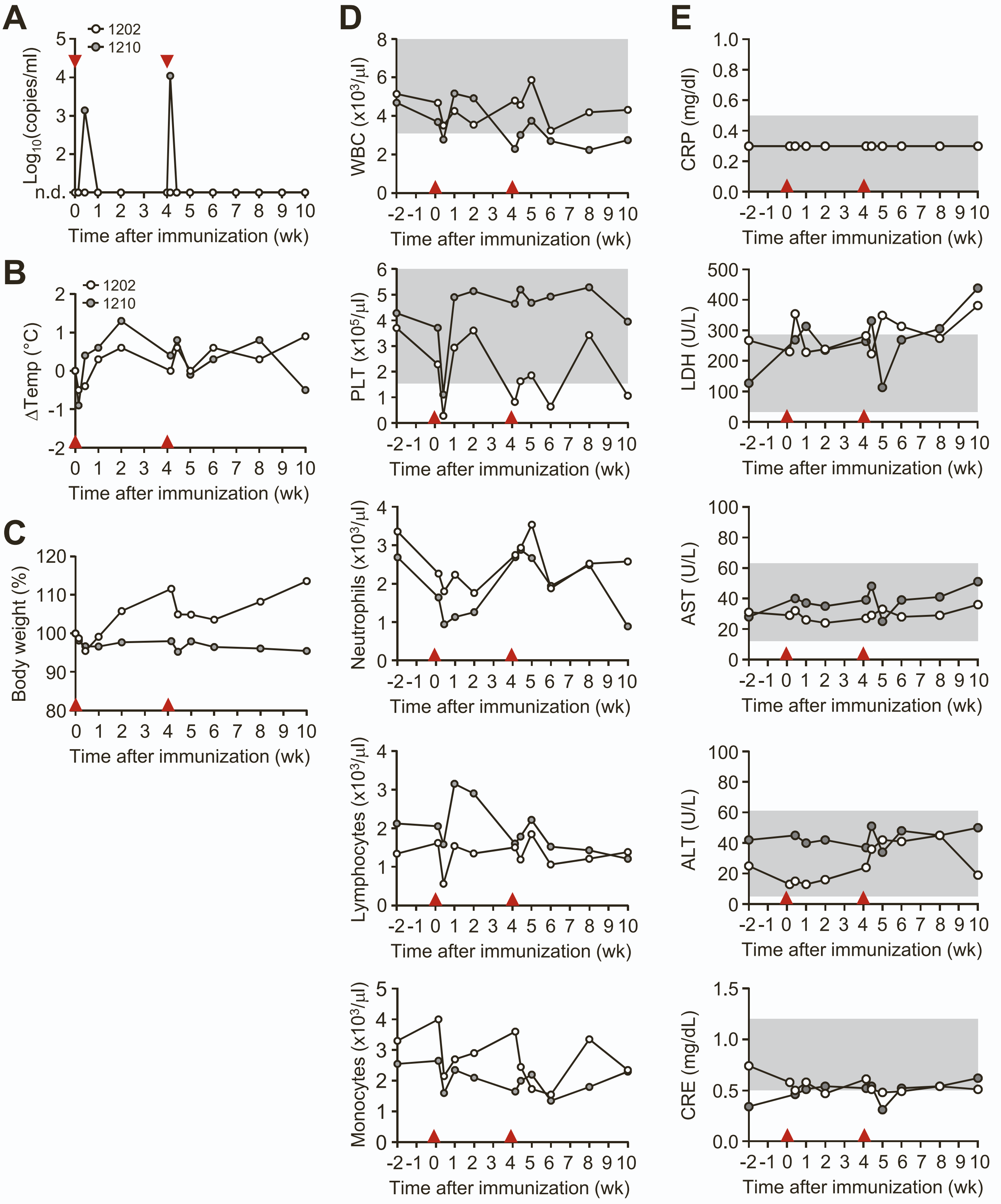

Figure S5. Hematological and biochemical monitoring following SFTSV ΔNS immunization in Rhesus macaques. Rhesus macaques were immunized twice with 10^6 FFU of SFTSV ΔNS at four-week intervals (i.m.). Red arrows indicate immunization time points. Body weight, temperature, viremia, complete blood count (CBC), and serum biochemistry were monitored over 10 weeks. **(A–C)** Viral RNA levels in serum **(A)**, body temperature **(B)**, and body weight **(C)** were measured at indicated time points. **(D)** Hematological parameters included WBC, platelet (PLT) count, neutrophils, lymphocytes, and monocytes. Gray shading indicates normal reference ranges for Rhesus macaques. **(E)** Biochemical markers included C-reactive protein (CRP), lactate dehydrogenase (LDH), AST, ALT, and creatinine (CRE).

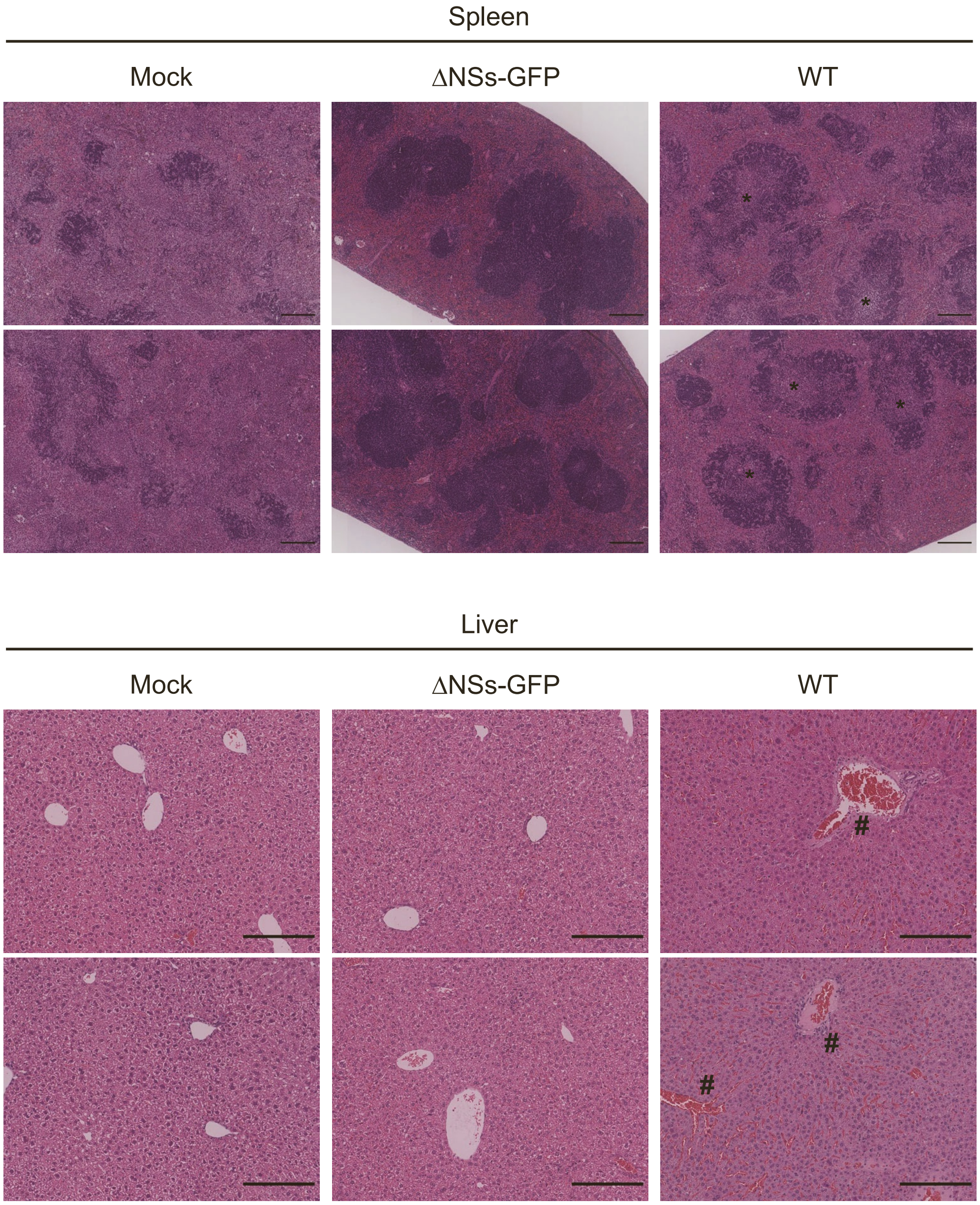

Figure S6. Histopathological analysis of IFNAR KO mice following infection with SFTSV. IFNAR KO mice were subcutaneously inoculated with 10^3 FFU of WT or ΔNSs-GFP SFTSV and sacrificed at day 3 post-infection. Representative hematoxylin and eosin (H&E)-stained spleen (left) and liver (right)sections. White pulp atrophy (*) in spleen and vascular congestion (#) in liver from mice infected with WT SFTSV are indicated. Scale bars, 200 µm.

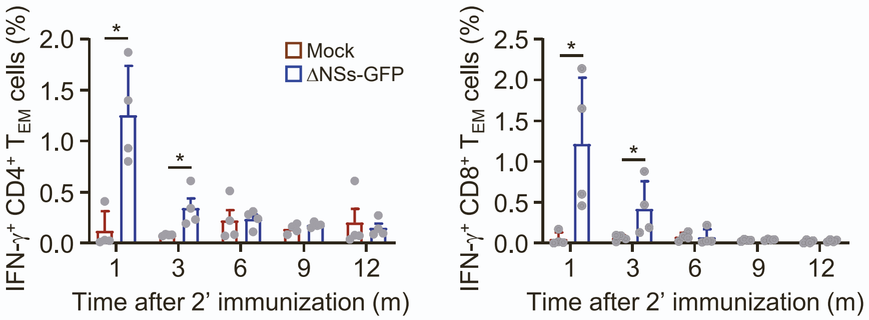

Figure S7. Immunization of recombinant SFTSV ΔNSs induces durable cellular immunity in IFNAR KO mice up to 3 months post-immunization. IFN-γ-producing $CD4^+$ and $CD8^+$ T cells were assessed by flow cytometry following NP stimulation of splenocytes collected at indicated time post-boost immunization (n = 4/group). *, $p < 0.05$ by Mann Whitney test.

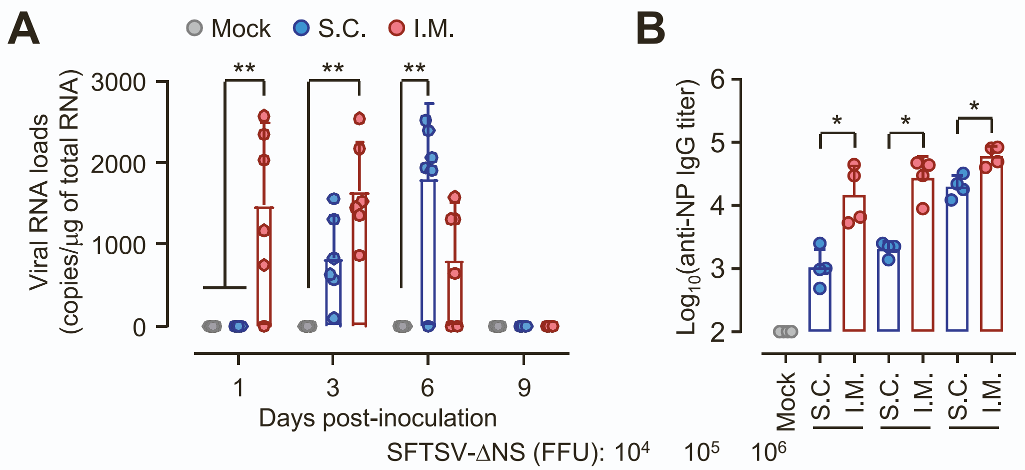

Figure S8. Differential viral dissemination and antibody responses according to inoculation route of SFTSV-ΔNSs in WT mice. (A) Viral RNA levels in draining (inguinal) lymph nodes at the indicated days post-inoculation (10^6 FFFU/mouse). **(B)** Anti-NP IgG responses measured two weeks after viral inoculation via the indicated routes and doses. S.C., subcutaneously; I.M., intramuscularly. *, $p < 0.05$; **, $p < 0.01$ by Kruskal Wallis test or Mann Whitney test.

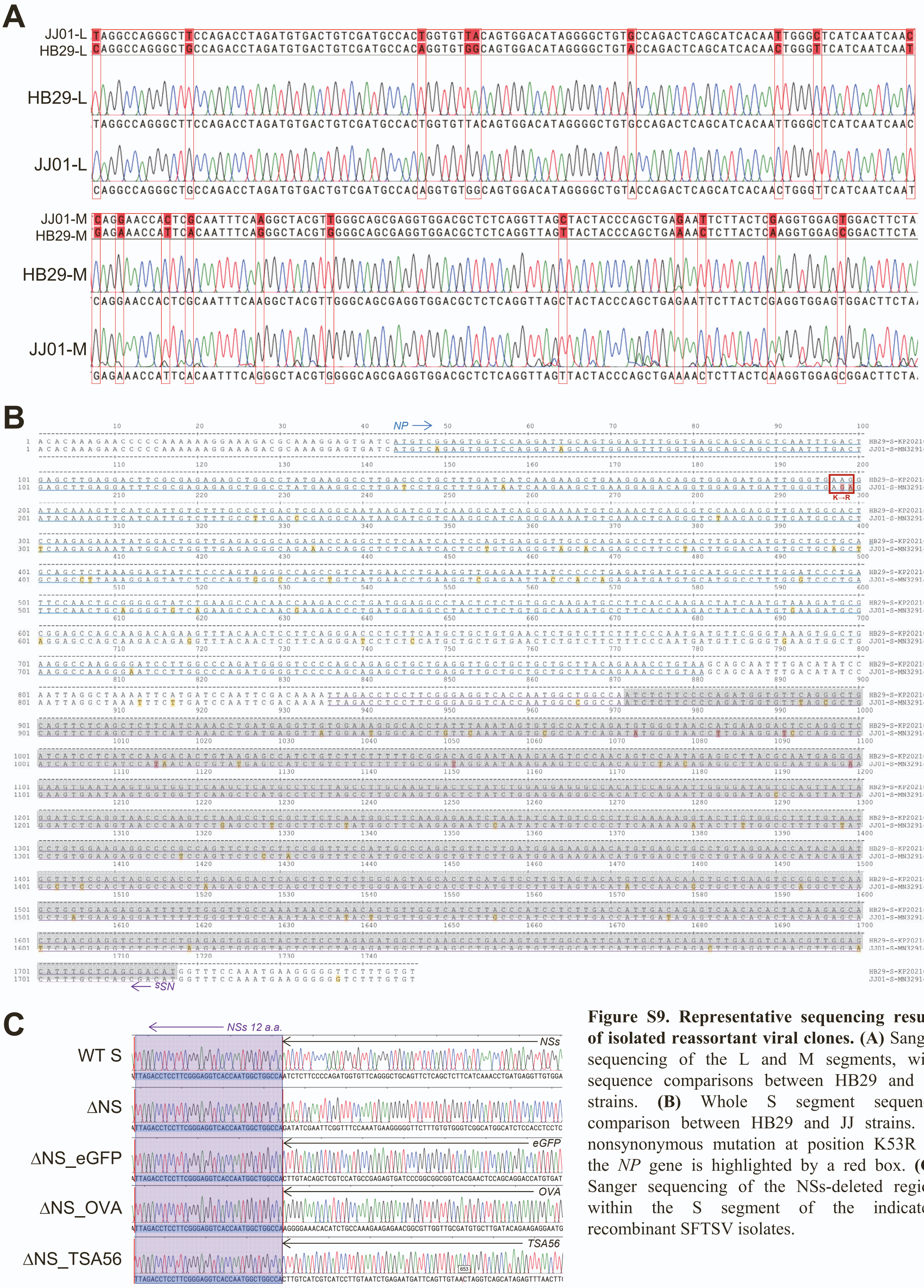

Figure S9. Representative sequencing results of isolated reassortant viral clones. (A) Sanger sequencing of the L and M segments, with sequence comparisons between HB29 and JJ strains. **(B)** Whole S segment sequence comparison between HB29 and JJ strains. A nonsynonymous mutation at position K53R in the *NP* gene is highlighted by a red box. **(C)** Sanger sequencing of the NSs-deleted region within the S segment of the indicated recombinant SFTSV isolates.

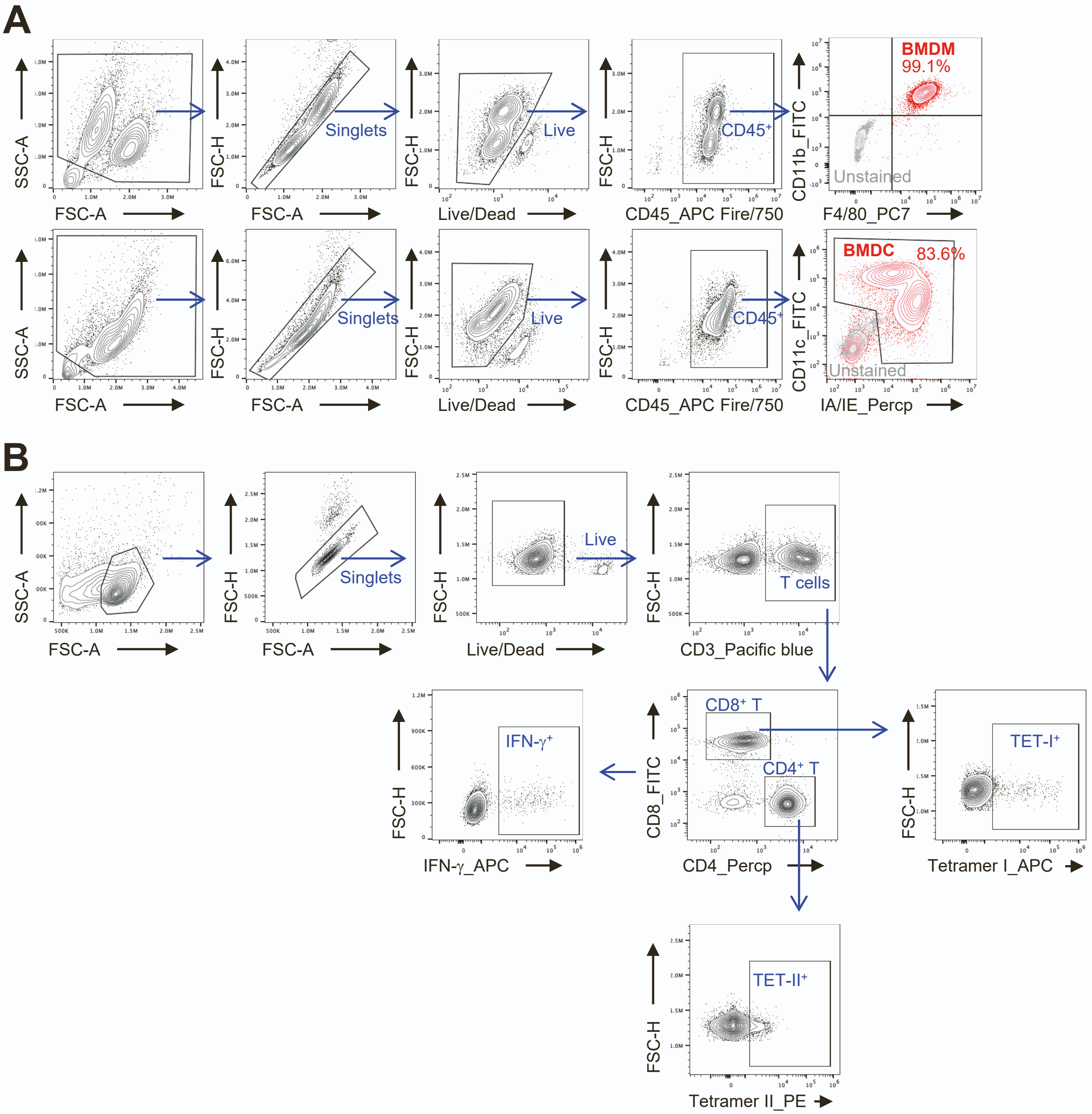

Figure S10. Representative flow cytometry gating strategies for APC and antigen-specific T cell identification. (A) Gating strategies for identifying BMDMs and BMDCs. BMDMs were defined as $CD11b^+$ $F4/80^+$ cells and BMDCs as $CD11c^+$ IA/IE^+ cells. **(B)** Antigen-specific T cells were gated on $CD3^+$ populations, with further identification of $CD4^+$ and $CD8^+$ subsets. Intracellular IFN-γ^+ cells, as well as OVA-specific tetramer I^+ and tetramer II^+ cells, were analyzed using appropriate isotype and negative controls.
