# Supplementary material for: Recombinant Dabie bandavirus as a bivalent vaccine platform inducing protective immunity against intracellular pathogens and cancer
Source: Mol Ther. 2025 Oct 24;34(2):1084–103. doi: 10.1016/j.ymthe.2025.10.044 (PMC12882354; doi:10.1016/j.ymthe.2025.10.044)
Supplement: Document S2. Article plus supplemental information [file mmc2.pdf]

Molecular Therapy
Original Article

Recombinant *Dabie bandavirus* as a bivalent vaccine platform inducing protective immunity against intracellular pathogens and cancer

Hyo-Jin Ro,[1,2,12] Yebeen Lee,[1,2,12] Kyeongseok Jeon,[1,2] Yujin Kim,[3] Seung Ho Baek,[3] Green Kim,[3] Joowan Kim,[1,2] Jun-Gu Kang,[4] Na-Yoon Jang,[1,2,5] Si-Hyeon Lee,[1,2] Sun-Young Kim,[1,2] Yu-Jin Kim,[1,2,5] Na-Young Ha,[6] Yuri Kim,[7] Young Ki Choi,[8] Jae U. Jung,[9] Jung Joo Hong,[3,10] and Nam-Hyuk Cho[1,2,5,11]

[1]Department of Microbiology and Immunology, Seoul National University College of Medicine, Seoul 03080, Republic of Korea; [2]Department of Biomedical Sciences, Seoul National University College of Medicine, Seoul 03080, Republic of Korea; [3]National Primate Research Centre, Korea Research Institute of Bioscience and Biotechnology (KRIBB), Cheongju, Chungcheongbuk-do 28116, Republic of Korea; [4]Korea Zoonosis Research Institute, Jeonbuk National University, Iksan 54531, Republic of Korea; [5]Institute of Endemic Disease, Seoul National University Medical Research Center, Seoul 03080, Republic of Korea; [6]Translational Immunology Institute, Chungnam National University, Daejeon 35015, Republic of Korea; [7]Department of Microbiology, Soon Chun Hyang University College of Medicine, Cheonan, Chungcheongnam-do 33151, Republic of Korea; [8]Center for Study of Emerging and Re-emerging Viruses, Korea Virus Research Institute, Institute for Basic Science (IBS), Daejeon 34126, South Korea; [9]Department of Infection Biology, Global Center for Pathogen and Human Health Research, Cleveland Clinic, Cleveland, OH 44195, USA; [10]KRIBB School of Bioscience, Korea University of Science & Technology (UST), Daejeon 34141, Republic of Korea; [11]Seoul National University Bundang Hospital, Seongnam, Gyeonggi-do 13620, Republic of Korea

Severe fever with thrombocytopenia syndrome virus (SFTSV) is an emerging tick-borne pathogen endemic to East Asia, associated with high mortality and lacking approved vaccines or therapies. We developed a live-attenuated vaccine platform based on a recombinant SFTSV, which lacks the nonstructural protein on the S segment (*NSs*) gene, a key virulence factor that suppresses type I interferon responses. The resulting ΔNSs viruses, reassorted with the prevalent genotype B, showed attenuated replication in interferon-competent cells while retaining the ability to infect and activate antigen-presenting cells (APCs). Immunization with the ΔNSs virus elicited robust SFTSV-specific humoral and cellular immune responses in mice and non-human primates, conferring complete protection against lethal challenge across multiple SFTSV genotypes, with immunity lasting up to 12 months. Additionally, recombinant ΔNSs viruses encoding heterologous antigens, such as ovalbumin (OVA) or type-specific antigen 56 (TSA56) from *Orientia tsutsugamushi*, induced strong antigen-specific T cell responses and conferred protection against OVA-expressing melanoma or scrub typhus, respectively. Mechanistically, ΔNSs viruses enhanced APC activation, improved antigen presentation, and reduced apoptosis in infected cells, supporting effective T cell priming. These findings establish SFTSV ΔNSs as an immunogenic and broadly protective vaccine candidate and a versatile bivalent vector platform for targeting viral, intracellular bacterial, and tumor-associated antigens.

INTRODUCTION

Dabie bandavirus, also known as severe fever with thrombocytopenia syndrome virus (SFTSV), is an emerging tick-borne pathogen responsible for severe fever with thrombocytopenia syndrome (SFTS) in humans.[1] SFTS is endemic to East Asia, with cases reported in China, South Korea, Japan, Vietnam, and neighboring countries.[2–10] The disease is characterized by high fever, thrombocytopenia, leukopenia, and multi-organ failure, with case fatality rates ranging from 10% to 30%.[2,11] *Haemaphysalis longicornis* ticks are the primary vectors of SFTSV,[12] and transmission occurs mainly through tick bites. However, both animal-to-human and human-to-human transmissions via blood or bodily fluids have also been documented.[13] Due to its epidemic potential, SFTSV has been designated a priority pathogen by the World Health Organization in 2017 and a category C agent by the US National Institute of Allergy and Infectious Diseases (NIAID).[14,15] Despite its clinical importance, there are currently no licensed vaccines or specific antiviral treatments available for SFTSV.

SFTSV belongs to the *Bandavirus* genus within the Phenuiviridae family.[1] It is a negative-sense, single-stranded RNA virus with a tripartite genome (L, M, and S segments) encapsulated by a lipid bilayer envelope. The L segment encodes the RNA-dependent RNA polymerase (RdRp), while the M segment encodes glycoprotein precursors that are processed into two major surface glycoproteins, Gn and Gc. These glycoproteins mediate viral entry, with Gn involved in receptor binding and Gc in membrane fusion.[16–18] The S segment encodes the viral

Received 8 April 2025; accepted 17 October 2025;
https://doi.org/10.1016/j.ymthe.2025.10.044.

[12]These authors contributed equally

Correspondence: Nam-Hyuk Cho, Department of Microbiology and Immunology, Seoul National University College of Medicine, Seoul 03080, Republic of Korea.
E-mail: chonh@snu.ac.kr

 © 2025 The Author(s).
Published by Elsevier Inc. on behalf of The American Society of Gene and Cell Therapy.
This is an open access article under the CC BY license (http://creativecommons.org/licenses/by/4.0/).

nucleoprotein (NP) and a nonstructural protein (NSs). NP is essential for genome encapsidation and replication.[19] The NSs protein is a well-characterized virulence factor that antagonizes host innate immunity, particularly through suppression of type I interferon (IFN) responses.[20–26] It disrupts antiviral signaling by inhibiting IFN-β induction and interfering with downstream pathways, including the TPL2 cascade.[26] These immune evasion functions make NSs an attractive target for genetic attenuation in vaccine development.

Various vaccine platforms have been explored for SFTSV, including protein subunits, DNA vaccines, whole-inactivated viruses, live-attenuated viruses, viral vectors, and mRNA-based approaches.[27] Most efforts have focused on full-length glycoproteins (Gn/Gc) or a single glycoprotein subunit (Gn) to induce neutralizing antibodies, often with limited cellular immune responses. Notably, a recent study showed that deletion or mutation of the *NSs* gene yielded a highly attenuated yet immunogenic virus that conferred complete protection in an aged ferret model, supporting the feasibility of live-attenuated SFTSV vaccines.[28]

Building on this, we developed a series of recombinant SFTSV vectors in which the *NSs* gene was deleted (ΔNSs) and replaced with various exogenous antigens, establishing the platform as a bivalent vaccine vector. Like other segmented RNA viruses, SFTSV shows high substitution and reassortment rates, contributing to the broad geographic circulation of diverse lineages and potentially influencing its virulence and fatality.[29] To ensure compatibility with circulating strains, the recombinant viruses were further reassorted with the JJ strain (genotype B), the most prevalent SFTSV genotype in South Korea and Japan.[30] The resulting recombinant viruses showed limited replication in IFN-competent cells while maintaining the ability to infect and activate antigen-presenting cells (APCs), such as dendritic cells (DCs), macrophages, and B cells. Consistently, this platform induced robust SFTSV-specific humoral and cellular immune responses in both mice and a non-human primate, rhesus macaques. In mice, immunization conferred complete protection against lethal challenge with multiple SFTSV genotypes, and protection was sustained for up to 12 months, indicating long-term memory. Antigen-specific antibody and T cell responses were also elicited in non-human primates, underscoring the translational potential of the platform. Furthermore, the vector efficiently expressed heterologous antigens, triggering strong antigen-specific T cell responses and protective cellular immunity against both cancer and an obligate intracellular pathogen in an antigen-dependent manner. Collectively, these findings highlight the versatility of the attenuated recombinant SFTSV as a bivalent vaccine vector capable of inducing protective immunity not only against SFTSV itself but also against unrelated intracellular pathogens and cancer.

RESULTS

Generation of an attenuated recombinant SFTSV encoding exogenous antigen

To generate attenuated recombinant SFTSV, we utilized a reverse genetics system based on the HB29 strain of SFTSV (genotype D).[31,32] Virulence attenuation was achieved by either deleting the *NSs* gene in the S segment, as previously reported,[26,28] or replacing it with an exogenous gene encoding enhanced green fluorescent protein (EGFP), ovalbumin (OVA), or type-specific antigen 56 (TSA56) derived from *Orientia tsutsugamushi*, an obligate intracellular bacteria causing scrub typhus (Figure 1A).[33] These recombinant viruses were further engineered through genetic reassortment with the wild-type (WT) SFTSV JJ strain (genotype B), which is the most prevalent genotype in South Korea and Japan.[32] The resulting reassortant viruses contained L and M segments from the JJ strain and a recombinant S segment from the HB29 strain. Expression of the viral NP and exogenous proteins was confirmed via immunofluorescence and immunoblot analyses following infection of Vero E6 cells (Figures 1B, 1C, and S1). We further examined the viral replication of the recombinant viruses in various host cells, including Vero E6, BJAB, murine bone-marrow-derived DCs (BMDCs), and murine bone-marrow-derived macrophages (BMDMs), and compared them with the parental SFTSV JJ strain (Figure 1D). The recombinant virus lacking the *NSs* gene and expressing GFP (SFTSV ΔNSs-GFP) exhibited replication kinetics similar to the WT SFTSV in Vero E6 cells, albeit with slightly lower efficiency. This observation aligns with the fact that Vero E6 cells have lost the ability to produce type I IFN due to spontaneous gene deletions.[34] In contrast, viral RNA replication of SFTSV ΔNSs-GFP in the type I IFN-competent BJAB cell line decreased from 24 h post-infection, indicating effective attenuation in the presence of an intact IFN response.[32] Conversely, the SFTSV WT continued to replicate robustly, overwhelming the competent host cells. Differential production of infectious virus in the culture supernatants of Vero E6 and BJAB cells was confirmed by focus-forming unit (FFU) assays (Figure 1E). A similar trend was observed in BMDMs derived from immunocompetent WT mice, where SFTSV ΔNSs-GFP replicated less efficiently and was eventually controlled, while SFTSV WT maintained higher replication levels (Figure 1D). Interestingly, both viruses replicated persistently and more efficiently in BMDCs derived from WT mice. These differences in replication kinetics between BMDMs and BMDCs were less pronounced in cells derived from IFN-α receptor1 (IFNAR)-knockout (KO) mice, indicating that both viruses can replicate efficiently in phagocytic APCs deficient in the innate immune response. Overall, these findings demonstrate that deletion of the *NSs* gene (SFTSV-ΔNSs) leads to reduced virulence *in vitro*, particularly in host cells, such as B cells (BJAB) and macrophages (BMDMs), with competent type I IFN responses.

Recombinant SFTSV efficiently activates professional APCs

We next investigated the activation of APCs derived from WT mice following infection with recombinant SFTSV. Infection of BMDCs, BMDMs, and B cells with either WT SFTSV or ΔNSs-OVA recombinant virus generally led to the upregulation of surface expression of co-stimulatory molecules, including CD40, CD80, and CD86, as well as major histocompatibility complex (MHC) class I and II molecules (Figure 2). Notably, surface expression of CD86 and MHC class I was significantly higher in cells infected with the ΔNSs-OVA virus compared to mock-infected controls (Figures 2F and 2G). Moreover,

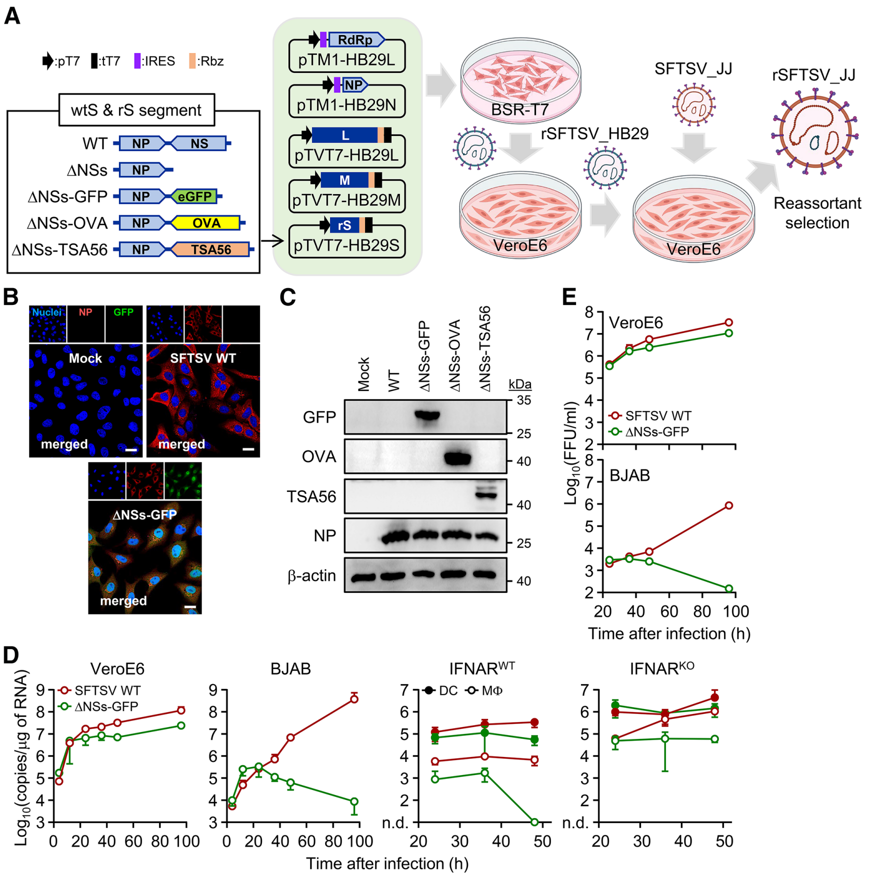

Figure 1. Generation and *in vitro* characterization of recombinant SFTSV ΔNS viruses

(A) Schematic of recombinant SFTSV ΔNSs generation. HB29-based plasmids were transfected into BSR-T7 cells, and supernatants were transferred to Vero E6 cells. Recombinant viruses were reassorted with the WT SFTSV JJ strain and plaque purified. (B) Immunofluorescence staining of Vero E6 cells infected with SFTSV WT or ΔNSs-GFP (MOI = 1) at 48 h after infection. Cells were fixed and stained with an anti-NP antibody. Mock-infected cells served as controls. Scale bar: 10 μm. (C) Immunoblot analysis of Vero E6 cells infected with SFTSV ΔNSs vectors expressing GFP, OVA, or TSA56 (MOI = 1) at 48 h after infection. (D) Replication kinetics of WT and ΔNSs-GFP viruses in Vero E6, BJAB, bone-marrow-derived dendritic cells (BMDCs), and bone-marrow-derived macrophages (BMDMs) from wild-type ($IFNAR^{WT}$) or interferon-receptor-deficient ($IFNAR^{KO}$) mice, as assessed by real-time qPCR of cell lysates. n.d., not detected. (E) Viral titers in supernatants of Vero E6 and BJAB cells infected with WT or ΔNSs-GFP, measured as FFUs/mL at the indicated time points. Data are shown as mean ± SD from triplicate measurements.

the expression levels of these markers induced by the recombinant virus were comparable to, or even higher than, those induced by WT SFTSV. These findings suggest that deletion of the *NSs* gene does not significantly impair, and may even enhance, the virus's ability to activate professional APCs, such as DCs and macrophages.

Apoptotic cell death of APCs infected with SFTSV may represent a pathogenic mechanism that impairs both innate and adaptive immune responses.[35] Indeed, a previous study reported that early-stage apoptosis of monocytes during lethal infection in human patients reduced antigen presentation by DCs, impaired differentiation and function of T follicular helper cells, and contributed to a failure in mounting virus-specific humoral responses.[36] To assess the extent of apoptosis in macrophages and DCs following infection with recombinant SFTSV, we infected BMDMs and BMDCs derived from WT and IFNAR-KO mice and analyzed necrotic and apoptotic cell death using annexin V and 7-aminoactinomycin D (7-AAD) staining (Figure S2). Only limited necrotic cell death was observed in BMDCs (average: 0.4%–1.3%) and BMDMs (average: 0.9%–5.4%), while a moderate increase in apoptosis was observed in BMDCs (average: 12.0%–39.0%) and BMDMs (average: 4.9%–12.0%) up to 2 days post-infection. Apoptotic cell death was slightly higher in DCs than in macrophages. Both the WT and recombinant SFTSV (ΔNSs-GFP) induced comparable levels of cell death, suggesting that the *NSs* gene plays a limited role in mediating apoptosis during infection in phagocytic APCs (Figure S2).

To assess whether viral infection induces APC activation and cell death directly or indirectly, we distinguished infected (GFP-positive) from uninfected (GFP-negative) populations within BMDM and BMDC cultures following infection with the recombinant ΔNSs-GFP virus and analyzed the proportions of apoptotic cells along with the expression levels of activation markers (Figures 3 and S3). As expected, the proportion of GFP-positive BMDCs and BMDMs was substantially higher in IFNAR-KO mice compared to WT mice (Figures 3A–3E). Interestingly, the frequency of both early and late apoptotic cells was higher among GFP-negative cells than in GFP-positive cells, suggesting that active viral infection and replication may suppress apoptosis in both IFNAR-KO and WT cells (Figures 3F and 3G). Furthermore, expression of all activation markers tested was markedly higher in GFP-positive cells from WT mice, with the exception of MHC class II in BMDMs, where uninfected (GFP-negative) cells displayed higher MHC class II expression than infected cells (Figures 3H and S3).

To further investigate the mechanistic basis underlying the differential responses of professional APCs to direct infection versus indirect stimulation, we performed transcriptome profiling of BMDCs infected with ΔNSs-GFP virus after sorting them into GFP-positive and GFP-negative populations (Figure 4). Principal-component analysis (PCA) of the transcriptome data clearly separated infected (GFP^+), non-infected (GFP^-), and mock-infected control cells (Figure 4A). Unsupervised hierarchical clustering of 1,222

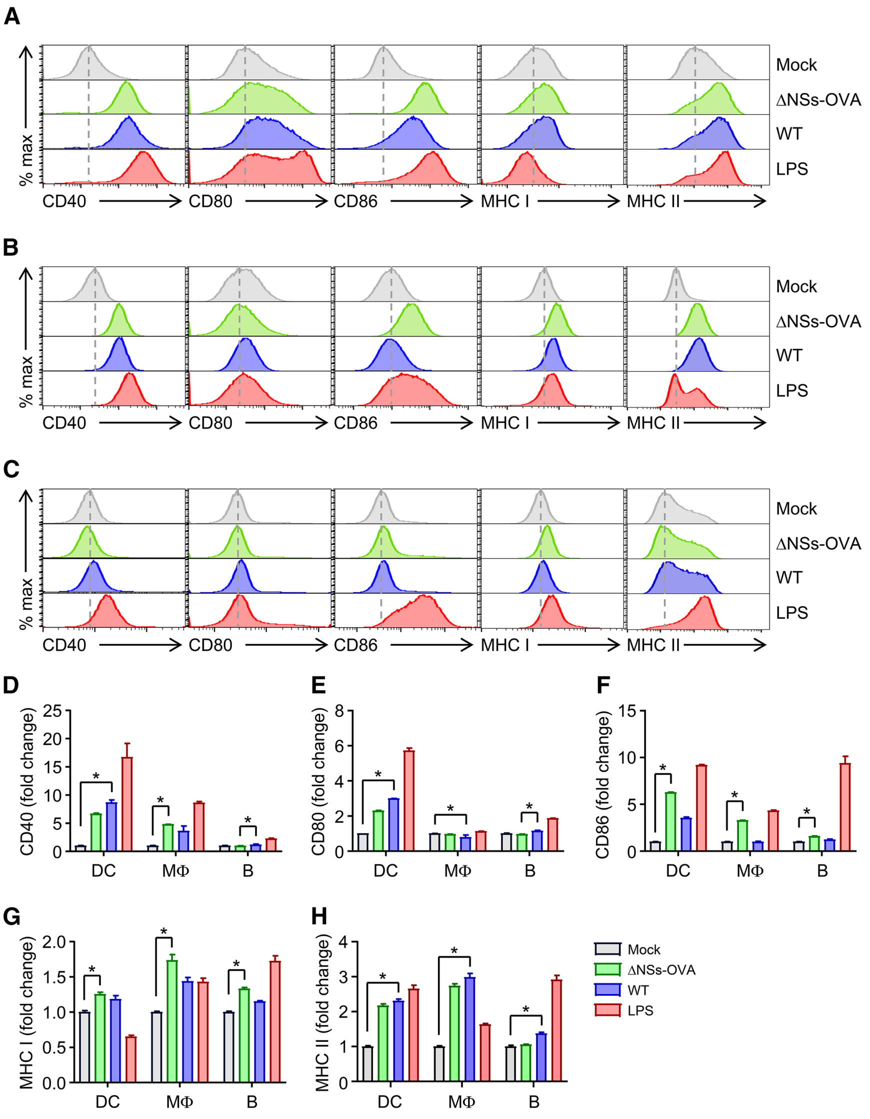

Figure 2. Recombinant SFTSV ΔNSs efficiently activate antigen-presenting cells

BMDCs, BMDMs, and splenic B cells from WT mice were infected with SFTSV ΔNSs-OVA or WT at an MOI of 1. After 24 h, surface expression of activation markers and MHC molecules was analyzed by flow cytometry. Lipopolysaccharide (LPS; 1 μg/mL) was used as a positive control. (A–C) Representative histograms showing surface marker expression in BMDCs (A), BMDMs (B), and B cells (C). (D–H) Quantification of co-stimulatory and MHC molecule expression levels in APC subsets. Expression of CD40 (D), CD80 (E), CD86 (F), MHC class I (G), and MHC class II (H) was measured. Mean fluorescence intensity (MFI) fold changes were calculated relative to mock-infected controls. Data are presented as mean ± SD from triplicate measurements. *$p < 0.05$ by Kruskal-Wallis test with post hoc multiple comparisons.

differentially expressed genes (DEGs) across experimental groups revealed five distinct clusters (C1–C5; Figures 4B and S4). C1 genes were strongly upregulated in GFP$^+$ cells compared with both GFP$^-$ and mock controls and were enriched for regulators of apoptosis, including both pro-apoptotic (e.g., *DDIT3*,[37] *FADD*,[38] *BID*,[39] and *BAD*[39]) and anti-apoptotic (e.g., *TRAF2*[40] and *MDM2*[41]) genes, indicating robust but bidirectional modulation of apoptotic pathways (Figures 4C–4E). C2 genes were preferentially induced in GFP$^-$ cells and included inflammatory mediators, such as *IL18*, *IL1B*, *TNF*, and *ICOS*, consistent with bystander activation in response to extracellular stimuli. C3 genes were elevated in both GFP$^+$ and GFP$^-$ populations and were primarily associated with antiviral activation and antigen presentation, including *IL12B*, *CD40*, *IFNB1*, *CD40*, *CD86*, and *MHC* molecules (Figure 4E, left). By contrast, C4 and C5 genes were downregulated either in GFP$^+$ or GFP$^-$ populations and encompassed pathways involved in endocytosis, cellular differentiation, and fatty acid metabolism. These transcriptional signatures are generally consistent with our phenotypic data shown in Figure 3, demonstrating enhanced antigen presentation and activation of DCs upon ΔNSs-GFP infection. Notably, the simultaneous induction of both pro-apoptotic (*DDIT3*, *FADD*, *DIABLO*,[42] *BAD*, and *BID*) and anti-apoptotic (*TRAF2*, *MDM2*, and *CLFAR*[43]) regulators highlights a complex, finely balanced apoptotic program triggered by the mutant viral infection (Figure 4E, right).

Taken together, these results indicate that direct infection of APCs, including DCs and macrophages, induces moderate levels of apoptosis but robust upregulation of co-stimulatory and MHC molecules, which is essential for the activation of antigen-specific T cell responses and the efficient induction of adaptive immunity.

Recombinant SFTSV induces antigen-specific immunity in mice and a non-human primate model

To evaluate the induction of antigen-specific adaptive immunity following immunization with the recombinant SFTSV-ΔNSs, we vaccinated WT mice intramuscularly twice and analyzed viral antigen-specific antibody and T cell responses 1 week after each immunization (Figure 5A). Primary inoculation with the recombinant virus (10^6 FFUs/mouse) caused minimal morbidity and limited systemic replication, as indicated by the absence of detectable viral RNA in the spleen, the primary target organ for SFTSV infection and replication (Figure 5B).[44,45] Viral RNA was undetectable in the lung and only transiently detected in the liver (3.1×10^2 copies/μg of total RNA in one of three mice). By contrast, low but sustained

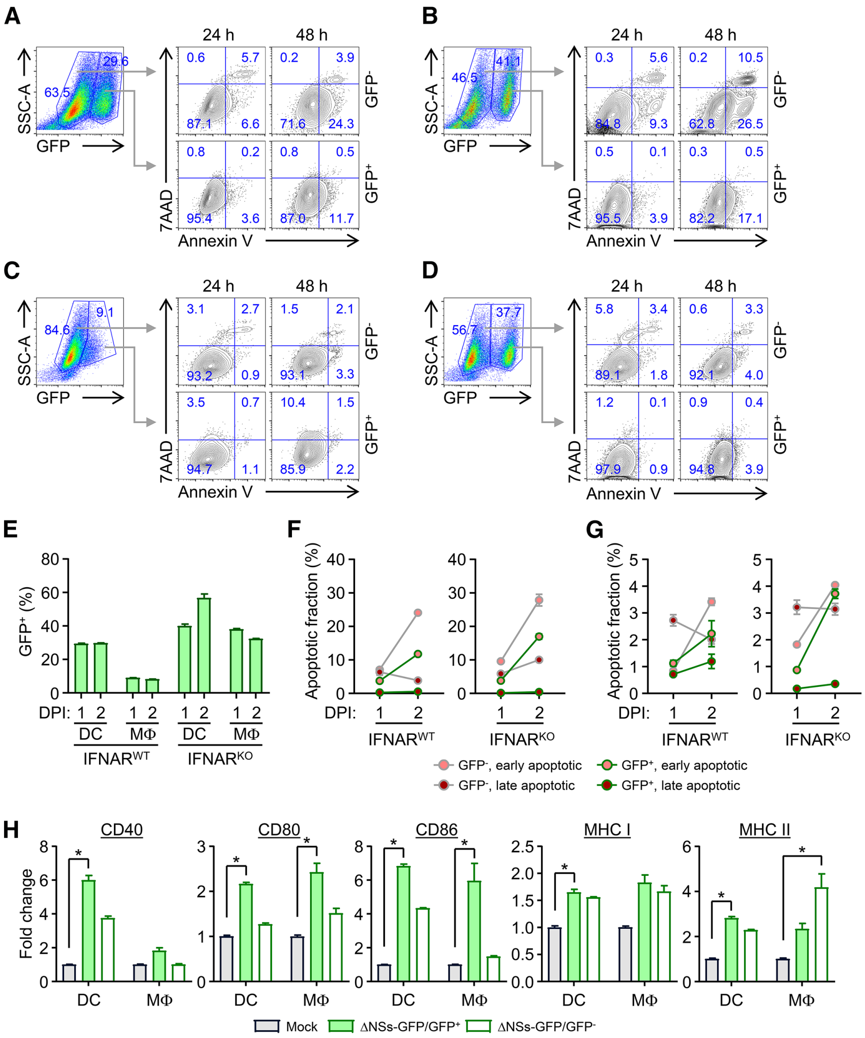

Figure 3. Differential apoptosis and activation profiles in SFTSV ΔNSs-GFP-infected and non-infected APCs

(A–G) BMDCs and BMDMs from WT or IFNAR-KO mice were infected with SFTSV ΔNSs-GFP at an MOI of 1. Apoptotic populations were analyzed by flow cytometry at 24 and 48 h post-infection. (A and B) Representative flow cytometry plots of BMDCs from WT (A) and KO (B) mice showing apoptotic populations in GFP^+ and GFP^- cells. (C and D) Representative flow cytometry plots of BMDMs from WT (C) and KO (D) mice. (E) Percentage of GFP^+ cells among BMDCs and BMDMs isolated from WT and KO mice at indicated time points. (F and G) Quantification of early apoptotic (Annexin V^+ $7\text{-}AAD^-$) and late apoptotic (Annexin V^+ $7\text{-}AAD^+$) fractions in BMDCs (F) and BMDMs (G) following SFTSV ΔNSs-GFP infection. (H) Expression levels of CD40, CD80, CD86, MHC class I, and MHC class II in GFP^+ versus GFP^- BMDCs and BMDMs from WT mice. Mean fluorescence intensity (MFI) fold changes were calculated relative to mock-infected controls. Mock, uninfected; ΔNSs-GFP/GFP^+, GFP-gated infected cells; ΔNSs-GFP/GFP^-, uninfected cells within infection cultures. Data are presented as mean ± SD from triplicate measurements. *$p < 0.05$ by Kruskal-Wallis test with post hoc multiple comparisons.

levels of viral RNA (~10^3 copies/μg of total RNA) were detected in the draining (inguinal) lymph nodes up to 6 days after the first inoculation. Serum biochemistry showed no evidence of systemic inflammation or organ injury compared with mock-infected controls (Table S1), indicating minimal systemic impact and *in vivo* toxicity of the attenuated virus in WT mice. Upon NP antigen stimulation, the frequency of IFN-γ-secreting $CD4^+$ and $CD8^+$ T cells in splenocytes increased markedly in a dose-dependent manner at 1 week after the booster immunization (Figure 5C). Antibody responses specific to the viral NP and Gn, including neutralizing antibodies, were also significantly elevated following primary and/or booster immunization in a dose-dependent manner (Figure 5D). We further validated the induction of antigen-specific adaptive immunity by vaccinating two non-human primates (rhesus macaques) with the recombinant SFTSV-ΔNSs virus. Following intramuscular inoculation with 10^6 FFUs/monkey, transient viremia was observed in one of the two animals. Both subjects exhibited a slight increase in body temperature and minor hematological changes, such as transient thrombocytopenia, within 1–2 days after the primary and/or secondary immunization (Figures S5A–S5D). Blood chemistry analyses revealed minimal systemic inflammatory responses following the sequential immunizations (Figure S5E). Despite these mild and transient effects, antigen-specific antibody responses against NP and Gn, as well as neutralizing antibodies, were enhanced after primary and booster immunizations (Figure 5E). Additionally, NP-specific T cell responses were markedly increased 1 week after the booster dose, as measured by ELISpot assays using peripheral blood mononuclear cells (PBMCs) (Figure 5F). Collectively, these findings demonstrate that the attenuated SFTSV lacking the *NSs* gene elicits strong viral antigen-specific humoral and cellular immune responses in both mouse and non-human primate models, without inducing significant clinical symptoms.

The recombinant SFTSV provides long-lasting protective memory against SFTSV infections

Next, we evaluated the protective efficacy of the adaptive immune response induced by immunization with the recombinant ΔNSs-GFP virus. A single inoculation of IFNAR-KO mice, which are highly susceptible to WT SFTSV infection,[46,47] with the attenuated recombinant virus caused minimal morbidity at a low dose (10^1 FFUs/mouse) and moderate weight loss at higher doses (10^3 or 10^5 FFUs/mouse) between days 4 and 6 post-inoculation (Figure 6A). All mice recovered rapidly thereafter. Transient viremia was detected

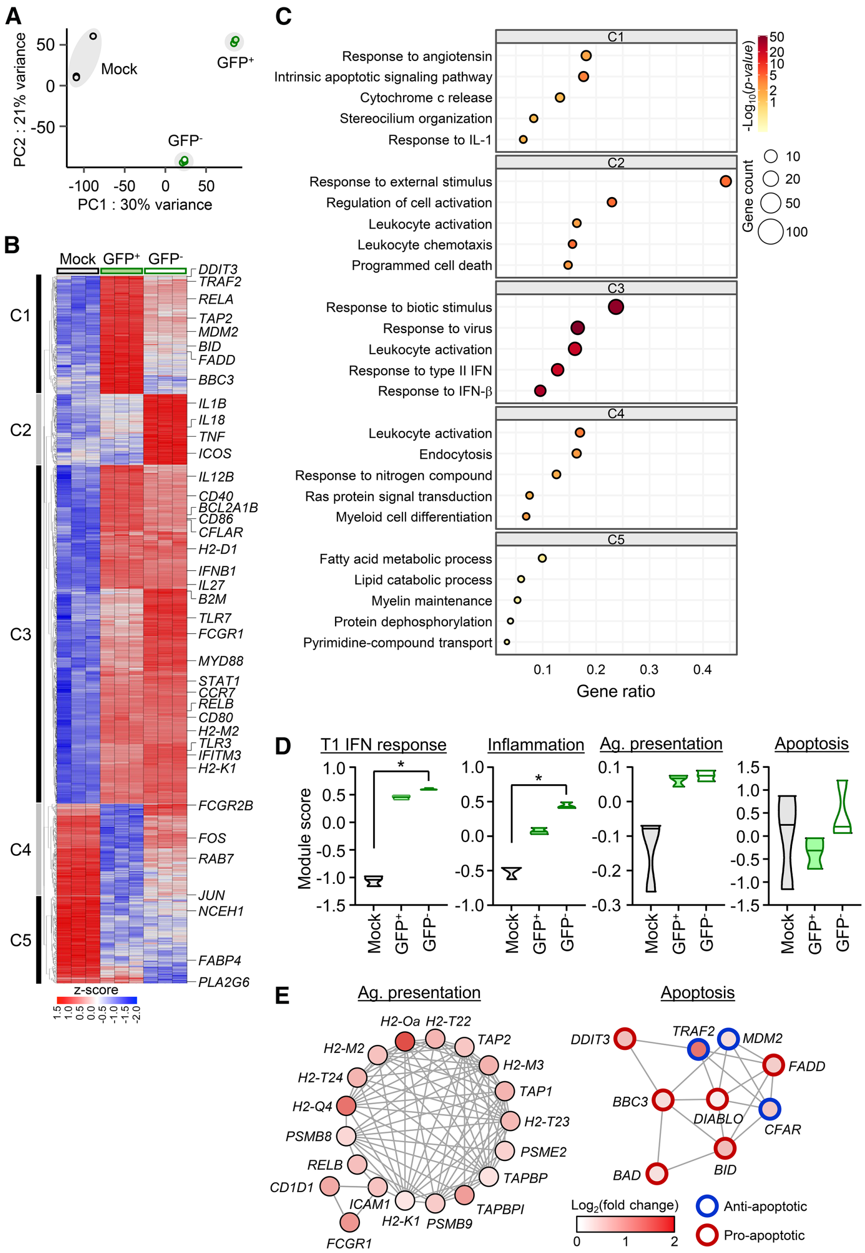

Figure 4. Transcriptomic profiling of BMDCs following ΔNSs-GFP infection

BMDCs were mock infected or infected with ΔNSs-GFP at an MOI of 1, and cells were sorted into GFP-positive (infected) and GFP-negative (bystander) populations for bulk RNA sequencing (RNA-seq) at 24 h post-infection. (A) PCA of transcriptomes from mock, GFP-negative (GFP^{-}), and GFP-positive (GFP^{+}) BMDCs (n = 3/group). (B) Heatmap of differentially expressed genes (DEGs) across groups. Genes with a $|\log_2$ (fold change [FC])$|$ > 1 and an FDR < 0.05 (relative to mock) were clustered into five modules (C1–C5) by unsupervised hierarchical clustering. (C) Gene Ontology (GO) enrichment analysis of DEGs within each cluster (C1–C5). The top five biological process terms are shown. (D) Module score analysis of type I interferon (IFN) response, inflammation, antigen (Ag.) presentation, and apoptosis pathways. Violin plots show score distributions across groups; *p < 0.05 by Kruskal-Wallis test with post hoc multiple comparisons. (E) Protein-protein interaction (PPI) networks of DEGs related to antigen presentation and apoptosis. Genes were selected based on differential expression between GFP^{+} and GFP^{-} BMDCs ($|\log_2(FC)|$ > 1, FDR < 0.05). Node fill color represents $\log_2$(FC) of GFP^{+} cells relative to mock controls, while node outline denotes functional annotation (red, pro-apoptotic; blue, anti-apoptotic).

in the spleen up to 6 days post-immunization, with complete viral clearance by day 9 (Figures 6B and 6C). In addition, mice developed mild to moderate thrombocytopenia in a dose-dependent manner. Histopathology showed that WT SFTSV induced marked hepatic congestion and white pulp atrophy in IFNAR-KO mice, whereas ΔNSs-GFP infection preserved normal tissue architecture and was associated with germinal center expansion (Figure S6). As expected, all the mock-immunized IFNAR-KO mice challenged with WT SFTSV (10^3 FFUs/mouse) succumbed to infection, exhibiting a sharp increase in splenic viral load and prolonged thrombocytopenia (Figures 6A–6C). In contrast, IFNAR-KO mice primed with a single dose of the recombinant virus survived the same WT SFTSV challenge without significant weight loss (Figure 6D), indicating that even a low priming dose (10^1 FFUs/mouse) conferred complete protection against lethal infection. Furthermore, mice immunized with recombinant SFTSV-ΔNSs (10^3 FFUs/mouse) were fully protected from lethal challenge (10^3 FFUs/mouse) with various SFTSV genotypes, including JJ (genotype B), HB (genotype D), and GW (genotype A) strains (Figure 6E), suggesting that immunization with the recombinant virus confers broad cross-genotypic protection.

To assess the durability of protective immune memory, IFNAR-KO mice immunized twice with recombinant SFTSV (10^3 FFUs/mouse) were challenged with WT SFTSV (10^3 FFUs/mouse) at 3, 6, 9, and 12 months post-immunization (Figure 6F). All vaccinated mice survived without significant morbidity or weight loss. At day 3 post-challenge, viral loads were significantly reduced or undetectable in vaccinated mice compared to mock-immunized controls (Figure 6G). Moreover, vaccinated mice did not develop thrombocytopenia, whereas control mice exhibited significant platelet loss (Figure 6H). Serological analysis revealed persistently high antibody titers against NP and Gn up to 9 months post-immunization, with only a slight decline observed at 12 months. Neutralizing antibody levels peaked at 3 months and

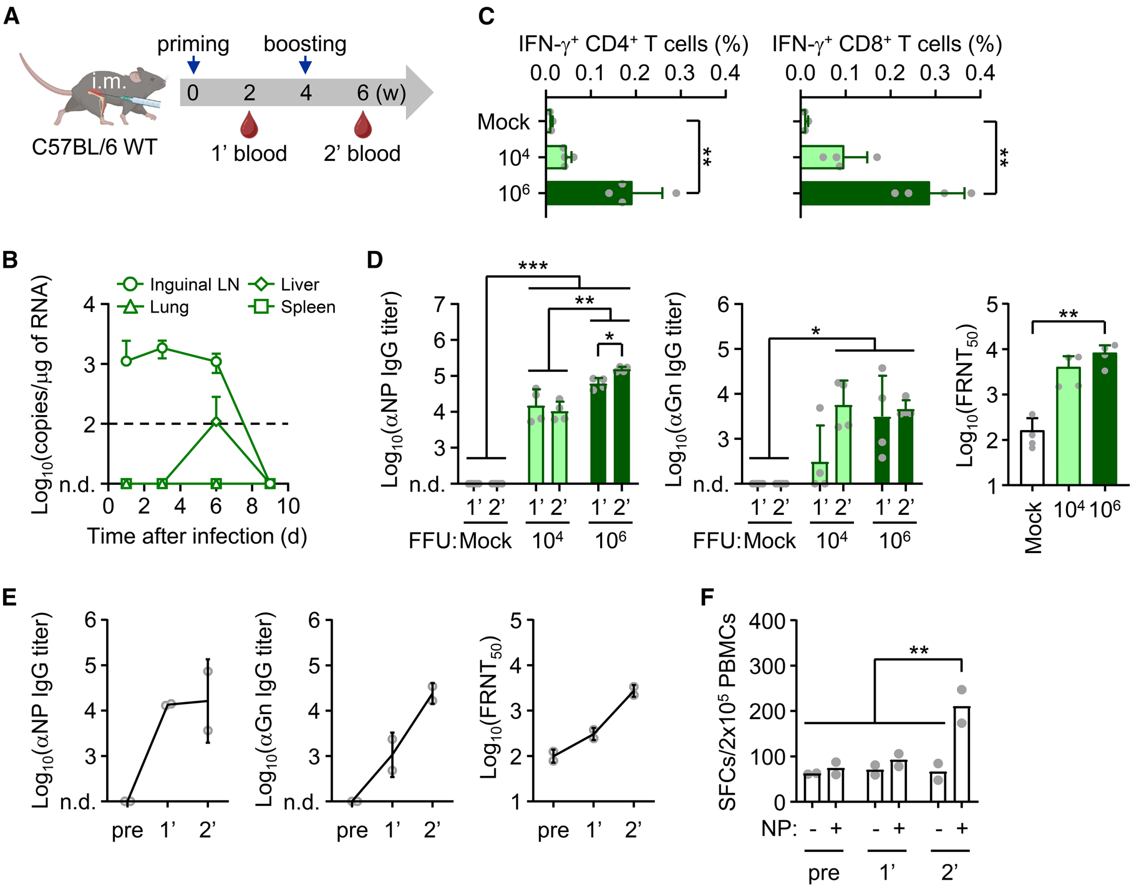

Figure 5. SFTSV ΔNSs induce humoral and cellular immune responses in mice and rhesus macaques

(A) Immunization schedule in WT mice receiving two intramuscular (i.m.) doses (10^4 or 10^6 FFUs) of SFTSV ΔNS-OVA at 2-week intervals. (B) Viral RNA loads in draining (inguinal) lymph nodes, spleen, liver, and lung were quantified by RT-qPCR at days 1, 3, 6, and 9 ($n = 3$/group). Dashed line: limit of detection. (C) IFN-γ-producing $CD4^+$ and $CD8^+$ T cells were assessed by flow cytometry following NP stimulation of splenocytes collected at 2 weeks post-boost immunization ($n = 4$/group). (D) SFTSV-specific IgG titers were measured across different doses (10^4 or 10^6 FFUs) and immunization schedule (prime or boost) groups, while neutralizing antibody titers were assessed in the boost groups (10^4 or 10^6 FFUs) ($n = 4$/group). (E and F) Rhesus macaques were immunized twice with 10^6 FFUs of SFTSV ΔNS at 4-week intervals (i.m.). Blood was collected 2 weeks after each immunization ($n = 2$/group). (E) NP- and Gn-specific IgG and neutralizing antibody titers were measured by ELISA and FRNT. (F) NP-specific T cell responses were evaluated by ELISpot following peptide pool stimulation (1 μg/mL per peptide). Data are presented as mean ± SD. *$p < 0.05$, **$p < 0.01$, and ***$p < 0.001$ by Kruskal-Wallis test with post hoc multiple comparisons or by Mann-Whitney test. n.d., not detected.

remained significantly elevated throughout the study, in contrast to negligible responses in mock-immunized mice (Figures 6I and 6J). In the case of antigen-specific T cell responses, both $CD4^+$ and $CD8^+$ T cell responses were significantly higher in mice inoculated with the recombinant virus compared to the mock-immunized group up to 3 months post-immunization, whereas the differences were not significant beyond 6 months (Figure S7). These findings suggest that long-lasting neutralizing antibodies play a central role in sustaining protective immunity.

To validate the contribution of humoral immunity, pooled immune sera obtained from mice 3–12 months post-immunization were passively transferred into naive recipients. Mice receiving immune sera exhibited significant resistance to lethal WT SFTSV challenge, whereas all control mice given non-immune sera succumbed to infection (Figure 6K). These findings confirm that long-lived neutralizing antibodies elicited by the recombinant SFTSV play a central role in protection. However, the passive transfer provided only partial protection at a lower challenge dose (10^1 FFUs/mouse) and merely delayed mortality at a higher dose (10^3 FFUs/mouse). This limited efficacy may reflect the single, relatively small volume of sera administered (150 μL/mouse) and further suggests that additional immune mechanisms beyond neutralizing antibodies likely contribute to the durable protection observed in immunized animals.

The bivalent SFTSV vector carrying an exogenous antigen confers protective cellular immunity against intracellular pathogen and cancer

Finally, we investigated the potential of recombinant SFTSV encoding an exogenous antigen as a vaccine vector to induce specific

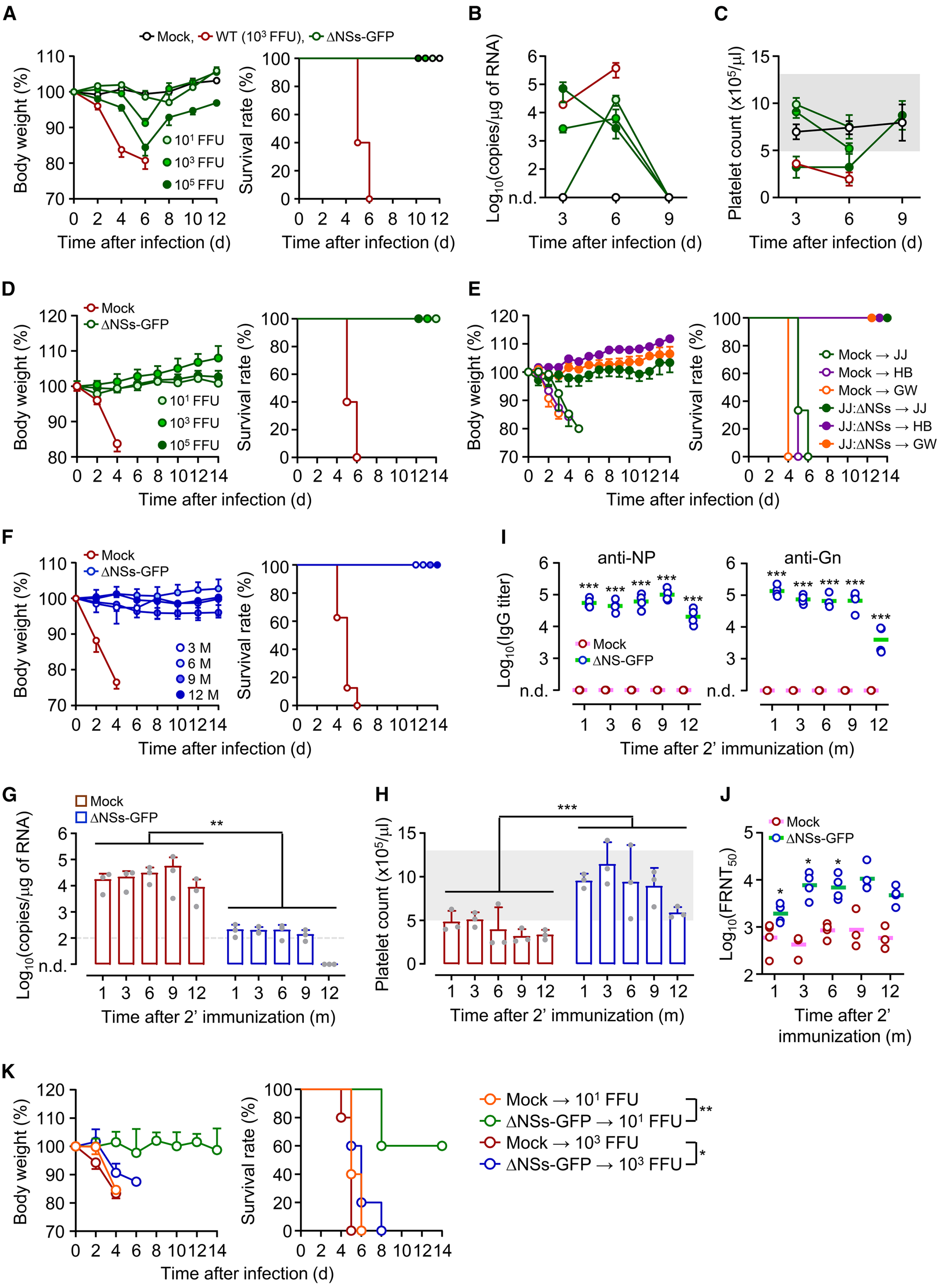

(legend on next page)

protective immunity. To this end, we infected BMDMs and BMDCs with recombinant SFTSV ΔNSs-OVA, which expresses the model antigen, OVA, and assessed antigen presentation via MHC class I by staining the cells with anti-H-2K^b:SIINFEKL antibodies. A significant increase in OVA peptide presentation via MHC class I was observed in APCs, particularly in DCs (Figures 7A and 7B). Notably, the surface expression level of the MHC class I:SIINFEKL complex in DCs infected with ΔNSs-OVA virus was comparable to that of cells treated with lipopolysaccharide (LPS) and SIINFEKL peptide, used as a positive control. Correspondingly, APCs, especially DCs, primed with the ΔNSs-OVA virus efficiently supported the *in vitro* proliferation of naive SIINFEKL-specific OT-I T cells (Figures 7C and 7D). *In vivo*, immunization of WT mice with the ΔNSs-OVA virus robustly induced OVA peptide-MHC tetramer-positive CD4$^+$ and CD8$^+$ T cells in the spleen 1 week after booster vaccination (Figure 7E). Furthermore, when these immunized mice were challenged with B16 melanoma cells expressing OVA, tumor growth was significantly suppressed compared to mice that were either mock immunized or vaccinated with the ΔNSs-GFP virus (Figure 7F), clearly demonstrating that the recombinant virus induced strong, antigen-specific protective cellular immunity against cancer.

To further evaluate the vaccine vector's utility against an obligate intracellular pathogen, we applied recombinant SFTSV ΔNSs-TSA56, expressing TSA56, a major outer membrane antigen of *O. tsutsugamushi*. Our previous study demonstrated that TSA56 confers protective immunity against lethal *O. tsutsugamushi* infection in mice.[48] In the current study, WT mice immunized twice with ΔNSs-TSA56 exhibited significantly higher frequencies of *O. tsutsugamushi*-specific CD4$^+$ and CD8$^+$ T cells in the spleen compared with mock-immunized or ΔNSs-GFP-immunized controls (Figure 7G). Moreover, ΔNSs-TSA56 immunization conferred significantly enhanced protection against lethal *O. tsutsugamushi* challenge relative to both control groups (mock and ΔNSs-GFP group), achieving protective efficacy comparable to that of mice immunized with the TSA56 subunit antigen (Figure 7H). Consistently, bacterial loads in the spleen at day 9 post-infection were significantly lower in ΔNSs-TSA56-immunized mice than in either negative control group (Figure 6I).

Collectively, these findings demonstrate that recombinant SFTSV vectors encoding exogenous antigens can elicit strong antigen-specific T cell responses, thereby providing protective cellular immunity against both intracellular pathogens and cancer.

DISCUSSION

Vaccines are fundamentally designed to elicit protective immune responses while maintaining acceptable safety margins. Over time, vaccine platforms have progressed from first-generation whole-pathogen vaccines to second-generation protein subunit and viral vector approaches and, most recently, to third-generation nucleic acid and nanoparticle-based systems.[49] For SFTSV, the principal antigenic targets have been the surface glycoproteins (Gn/Gc), which mediate viral entry, and the NP to a lesser extent. Comparative studies and systematic reviews suggest that platforms capable of *in situ* antigen expression (e.g., live-attenuated viruses, viral vectors, and DNA and mRNA vaccines) generally induce more robust neutralizing antibody and T cell responses than protein subunit vaccines, though no licensed SFTSV vaccine exists to date.[27,50,51] mRNA vaccines encoding Gn or Gn/Gc antigens consistently generate high neutralizing titers and complete protection in lethal mouse challenge models.[52–55] Their strengths include rapid design and scalable manufacture, but limitations remain: reliance on lipid nanoparticle (LNP) delivery, greater reactogenicity compared with protein vaccines, stringent cold-chain requirements, and as yet unproven durability and cross-genotype breadth in larger models. Protein subunit vaccines (e.g., soluble Gn/Gc or nanoparticle formulations) are the safest and most scalable option and can achieve solid protection when paired with potent adjuvants or used in heterologous prime-boost regimens.[47,56] However, they typically require multiple doses or strong adjuvants to achieve the cellular immunity observed with gene-based platforms. Several viral vector platforms have shown promise for SFTSV vaccination, including adenovirus, vesicular stomatitis virus (VSV), vaccinia virus (VACV), and rabies-virus-based vectors. Recombinant VSV (rVSV) and recombinant VACV (rVACV) expressing SFTSV Gn/Gc antigens protected animals from lethal infection, with rVSV-Gn/Gc achieving 100% survival even in aged, IFNAR-deficient mice.[57–59] However, some studies lacked T cell response data, leaving cellular immunity unverified. Notably, rVSV-Gn/Gc also conferred cross-protection against Heartland virus, a closely related bandavirus, highlighting its potential for broad-spectrum protection within the genus.[59] Other vectors include rabies-virus-based vaccines and recombinant adenovirus type 5 (rAd5) platforms expressing Gn.[60–62] These induced humoral and cellular responses, though protection against lethal challenge was inconsistently assessed. A heterologous rAd5 prime-protein boost strategy showed promise[63] but was limited by high global Ad5 sero-prevalence, which may reduce efficacy.[64] Similarly, pre-existing immunity compromised rVACV efficacy[58] but has not been

Figure 6. Immunization of recombinant SFTSV ΔNSs confers durable protective immunity in IFNAR-KO mice

(A–C) IFNAR-KO mice were subcutaneously (s.c.) injected with varying doses of SFTSV WT or ΔNSs-GFP. (A) Body weight and survival were monitored (n = 5/group). (B and C) Viral loads (B) and platelet counts (C) were measured in the spleen at days 3, 6, and 9 (n = 3/group). (D) Mice immunized with ΔNSs-GFP were challenged with 10^3 FFUs of WT SFTSV 2 weeks later (s.c.). Body weight and survival were tracked (n = 5/group). (E) Cross-genotypic protection was evaluated using 10^3 FFUs of JJ, HB29, or GW WT SFTSV strains (n = 5/group). (F–J) To assess long-term immunity, mice were immunized twice with 10^3 FFUs of ΔNSs-GFP and challenged at 3, 6, 9, or 12 months. (F) Body weight and survival were recorded (n = 5/group). (G and H) Spleen viral loads (G) and platelet counts (H) were measured on day 3 post-challenge (n = 3/group). (I and J) NP- and Gn-specific IgG titers (I) and neutralizing antibody levels (J) were assessed by ELISA and FRNT (n = 4/group). (K) For passive transfer, pooled immune sera from immunized mice were administered to naive recipients (150 μL/mouse) 1 day before WT SFTSV challenge as indicated. Body weight and survival were monitored (n = 5/group). *$p < 0.05$, **$p < 0.01$, and ***$p < 0.001$ by Kruskal-Wallis test or by log rank test (survival curves). n.d., not detected.

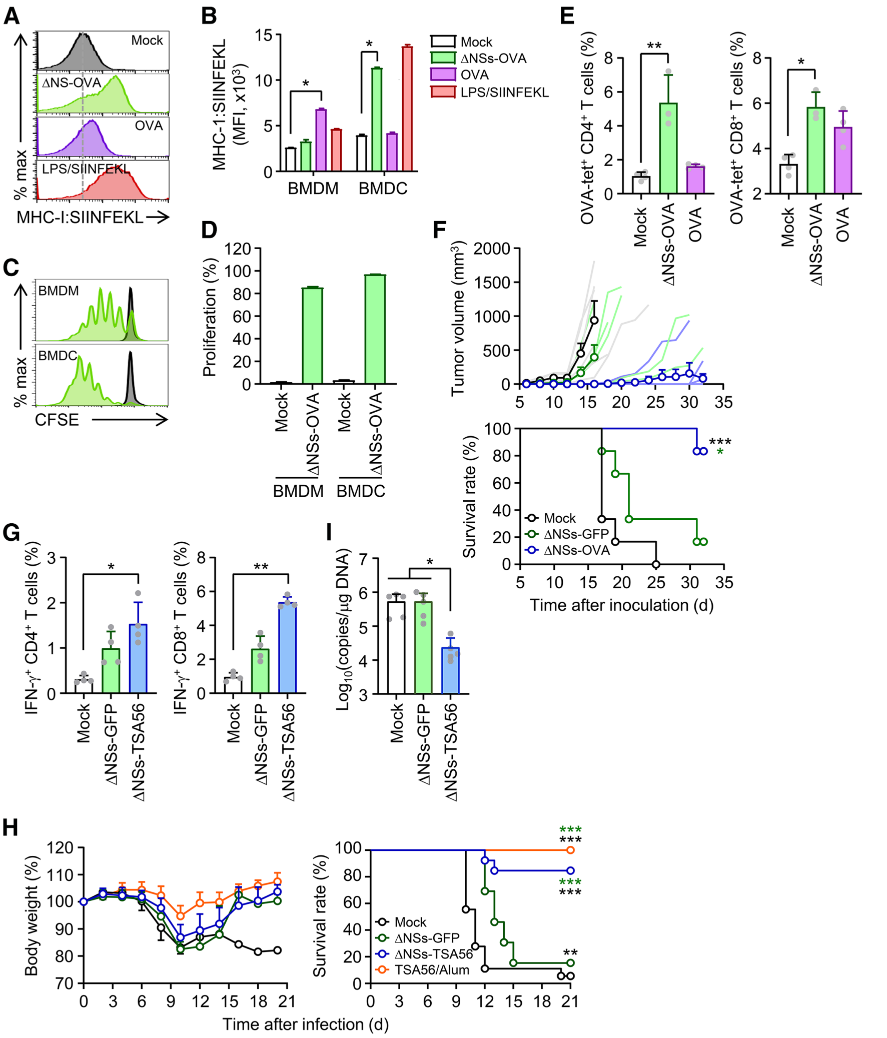

Figure 7. SFTSV ΔNS vectors expressing heterologous antigens elicit protective cellular immunity against tumor and *O. tsutsugamushi* infection

(A and B) Antigen presentation was assessed in WT BMDMs and BMDCs infected with ΔNSs-OVA (MOI = 1) or treated with OVA protein (10 μg) at 24 h after infection. LPS and SIINFEKL peptide (1 μg/mL) served as positive controls. Surface H-2Kb:SIINFEKL complex expression was measured by flow cytometry. (C and D) T cell proliferation was evaluated by CFSE dilution after co-culture of ΔNSs-OVA-infected APCs with CFSE-labeled OT-I splenocytes for 3 days. (E) OVA-specific CD4$^+$ and CD8$^+$ T cells were detected using MHC tetramers in mice immunized with ΔNSs-OVA (10^6 FFUs, i.m., twice) or OVA protein with Alum adjuvant (10 μg, s.c., three times) (n = 3/group). (F) Mice immunized twice with ΔNSs-OVA were challenged with 5×10^5 B16MO5 melanoma cells (s.c.). Tumor growth was monitored every 2 days; mice were euthanized when tumors exceeded 2,000 mm^3 (n = 6/group). (G–I) WT mice were immunized twice with ΔNSs-GFP or ΔNSs-TSA56 (10^6 FFUs, i.m.) and intravenously (i.v.) challenged with 2×10^5 FFUs of *O. tsutsugamushi*. (G) IFN-γ-producing CD4$^+$ and CD8$^+$ T cells were analyzed by intracellular cytokine staining following TSA56 stimulation (10 μg) (n = 4/group). (H) Body weight and survival were monitored in mock-immunized mice (PBS, n = 18) and in mice immunized with ΔNSs-GFP (n = 13), ΔNSs-TSA56 (n = 13), or TSA56 protein formulated with Alum adjuvant (10 μg per dose, administered twice at 2-week intervals, n = 7). (I) Lung bacterial loads were measured by RT-qPCR on day 7 (n = 5/group). Black asterisks indicate significance versus mock; green asterisks indicate significance versus ΔNSs-GFP. Data are presented as mean ± SD. *$p < 0.05$, **$p < 0.01$, and ***$p < 0.001$ by Kruskal-Wallis test with post hoc multiple comparisons or by log rank test (survival curves).

evaluated for rVSV vectors, an important gap. Though VSV-based vectors are immunogenic, they can cause mild systemic effects and transient viremia.[65,66] Safety concerns, such as vector genome integration, recombination, or reversion, apply broadly across viral vector platforms.[67,68] Live-attenuated SFTSV vaccines, particularly ΔNSs variants, provide the additional advantage of presenting the full viral antigen repertoire while avoiding pre-existing vector immunity. These viruses have demonstrated robust immunogenicity, protective efficacy across various genotypes, and versatility for expressing heterologous antigens.[28] Nonetheless, translation is constrained by the high-consequence nature of SFTSV, necessitating rigorous biosafety containment, extensive nonclinical safety testing (dose escalation, shedding and reversion risk, and neuro- or viscerotropism), and cautious population targeting. Overall, recent syntheses highlight that platform choice must balance immunogenic breadth (including cross-strain neutralization), durability, safety margins in vulnerable populations, vector-specific risks, and logistical feasibility (cold-chain, dose number, and cost). Among emerging strategies, heterologous combinations, such as viral vector or mRNA priming followed by protein boosting, appear especially promising, offering a practical way to maximize both the breadth and durability of protection while mitigating safety and deployment challenges.[63,69]

From a cost-effectiveness perspective, protein subunit platforms generally incur the lowest cost of goods and a more manageable cold chain, supporting more favorable incremental cost-effectiveness ratios (ICERs), though the requirement for multiple doses or adjuvant systems increases programmatic cost risk. mRNA platforms allow rapid strain adaptation but currently carry higher manufacturing and formulation costs and stricter cold-chain demands; their cost effectiveness improves if durable immunity can be achieved with fewer doses. Empirical vaccine economic analyses in other domains suggest mRNA vaccines are sometimes less cost effective when the dose cost is high (e.g., comparisons in COVID-19 vaccine modeling),[70,71] whereas viral vector vaccines often strike a more favorable balance between efficacy and cost in resource-limited

settings, as shown in Nigeria's COVID vaccine modeling.[72] The fixed costs associated with biosafety-level-compliant manufacturing, enhanced safety monitoring, and regulatory burden could reduce the cost advantage of live-attenuated SFTSV vectors unless uptake is high and health gains substantial. Ultimately, rigorous economic modeling, accounting for local healthcare costs, disease burden, logistics, vaccine uptake, durability, and genotype coverage, will be required to compare platforms reliably; our immunogenicity and protection data furnish key input parameters, but a definitive cost-effectiveness ranking must await full pharmacoeconomic modeling.

The *NSs* gene of SFTSV is a well-characterized virulence factor known to suppress host innate immunity by antagonizing type I IFN signaling.[20–25] Accordingly, deletion of the *NSs* gene using a reverse genetics system led to the generation of an attenuated SFTSV strain, which conferred complete protection against lethal challenge in aged ferret models.[28] In the present study, we extended this approach by engineering ΔNSs recombinant SFTSV vectors capable of expressing exogenous antigens (Figures 1A–1C). These recombinant viruses demonstrated attenuated replication in IFN-competent cells, retained the capacity to activate professional APCs, and elicited potent antigen-specific humoral and cellular immune responses in both murine and non-human primate models. Notably, the ΔNSs platform conferred complete and broad protection against lethal SFTSV challenge across multiple genotypes, with protective immunity sustained for up to 12 months post-vaccination in the mouse model. Additionally, the platform provided immune protection against intracellular bacterial infection and cancer. We selected these two disease models to assess the functional relevance of SFTSV-ΔNSs-induced T cell responses in distinct but clinically important contexts. The B16 melanoma model is a well-established system for evaluating cytotoxic T cell-mediated tumor control, and similarly, *O. tsutsugamushi*, the causative agent of scrub typhus, was chosen because protection against this obligate intracellular bacterium critically depends on cellular immunity, particularly $CD8^+$ T cell responses.[48] Notably, scrub typhus shares seasonal prevalence and risk groups (e.g., elderly farmers) with SFTS in endemic regions.[7,73]

The ability of the SFTSV-ΔNSs vector to express and present heterologous antigens, such as OVA and TSA56, underscores its potential as a versatile bivalent vaccine platform. Consistent with this, recombinant SFTSV-ΔNSs-OVA effectively suppressed OVA-expressing B16 melanoma (Figure 7F), likely through efficient MHC class I-mediated peptide presentation by DCs (Figures 7A and 7B) and expansion of antigen-specific effector T cells (Figures 7C–7E). Similarly, SFTSV ΔNSs-TSA56 robustly elicited TSA56-specific $CD4^+$ and $CD8^+$ T cell responses, which facilitated the clearance of *O. tsutsugamushi* and enhanced survival following lethal challenge (Figures 7G and 7H). Given the critical role of cellular immunity in both tumor surveillance and defense against intracellular bacterial pathogens,[48,74–76] the ΔNSs-based platform offers a promising "two-birds-with-one-stone" strategy for broad protection against both scrub typhus and SFTS.

Mechanistically, the *NSs* gene's role as an IFN antagonist, its deletion markedly reduced viral replication in IFN-competent cells while allowing productive infection in Vero E6 and IFNAR-deficient cells (Figures 1D and 1E). This IFN-sensitive attenuation enhances the platform's safety profile. Interestingly, SFTSV replication varied across APC subsets: both WT and ΔNSs viruses replicated similarly in BMDCs, but the WT virus showed higher replication in BMDMs (Figure 1D), suggesting cell-type-specific replication mechanisms. Despite differences in replication dynamics, both WT and ΔNS viruses robustly activated APCs (Figure 2). ΔNSs viruses induced expression of co-stimulatory molecules (CD40, CD80, and CD86) and MHC class I and II at levels comparable to, or greater than, those induced by WT virus. Direct infection with the SFTSV ΔNSs-GFP virus also enhanced MHC expression in GFP^+ cells (Figure 3H), supporting efficient priming and proliferation of antigen-specific T cells, an essential feature of effective vaccine-mediated immunity. Furthermore, the transcriptional signatures of BMDCs infected with the ΔNSs virus revealed a mechanistic basis for APC activation via both direct infection and indirect stimulation, ultimately leading to enhanced antigen presentation capacity (Figure 4). Interestingly, MHC class II expression was selectively reduced in GFP^+ macrophages compared to GFP^- (non-infected) cells, despite elevated expression of other co-stimulatory molecules in the GFP^+ population. This suggests virus-driven, cell-type-specific modulation of antigen presentation. One plausible mechanism involves interference with the class II transactivator CIITA, the master regulator of MHC class II expression, which is controlled by cell-type-specific promoters (pI, pIII, and pIV).[77,78] The macrophage-specific downregulation of MHC class II, without a global reduction in co-stimulatory markers, implies that SFTSV may selectively suppress CIITA transcription. Similar immune evasion strategies have been documented in other viruses, such as herpesviruses, which suppress CIITA or disrupt MHC class II transport via viral proteins.[79–81] However, further research is needed to determine whether SFTSV directly targets CIITA pathways in macrophages.

In parallel, we observed limited apoptosis in ΔNSs-GFP-infected APCs (Figures 3A–3G), indicating that infection supports cellular activation while minimizing cytopathic effects and preserving APC function. Notably, DCs and macrophages that were directly infected (GFP^+) exhibited lower apoptosis than uninfected bystanders (GFP^-), and this pattern was similar in WT and IFNAR-deficient cells, suggesting independence from type I IFN receptor signaling. Previous studies on SFTSV-induced apoptosis have primarily relied on bulk population analyses,[82,83] which fail to distinguish between infected and uninfected cells. By separating GFP^+ and GFP^- subsets, our data indicate that SFTSV-ΔNSs may engage intrinsic survival pathways in infected cells to delay death while maintaining function. In GFP^+ cells, pro-apoptotic mediators (*DDIT3/CHOP*, *BID*, *BAD*, *BBC3/PUMA*, and *DIABLO/SMAC*) were upregulated, consistent with endoplasmic reticulum (ER) and mitochondrial stress,[84–86] while anti-apoptotic regulators (*CFLAR/c-FLIP*, *TRAF2*, and *MDM2*) and survival signaling genes (*NF-κB[RELA]*, *PKCε*,[87] *NHERF1*,[88] and *HIF1α*[89]) were concomitantly induced (Figures 4 and S4). This dual

induction supports a "primed-but-restrained" state in which apoptotic programs are transcriptionally engaged yet functionally delayed, preserving antigen presentation. By contrast, GFP^{-} bystanders showed weaker intrinsic apoptotic signatures, with limited *BBC3* and *DIABLO* induction, implying a greater reliance on extrinsic cues (e.g., tumor necrosis factor [TNF] or type I IFN). Similar dichotomous regulation of apoptosis has been reported in other viral infections, where the ultimate cell fate depends on the interplay between pro- and anti-apoptotic signals. For example, hepatitis B virus (HBV) can promote hepatocyte survival by upregulating *BCL-2*, yet its HBx protein has also been shown to sensitize cells to apoptosis in a genotype- and context-dependent manner,[90] paralleling the mixed regulatory pattern we observed in SFTSV-ΔNSs. Likewise, human cytomegalovirus (HCMV) activates the PI3K/Akt pathway and encodes the viral Bcl-2 homolog, viral mitochondria-localized inhibitor of apoptosis (vMIA), to inhibit the intrinsic pathway,[91] while herpes simplex virus (HSV) employs proteins such as ICP6 to directly suppress initiator caspase-8 activity.[92] These parallels suggest that SFTSV likewise manipulates cell death programs to prolong the survival of infected APCs while leaving neighboring immune cells more vulnerable to apoptosis. Interestingly, a recent study demonstrated that SFTSV NSs interacts with the kinase domain of RIPK3 to form biocondensates, thereby promoting RIPK3 autophosphorylation and activation of necroptosis.[93] Moreover, the SFTSV-$NSs^{N122A-S123A}$ mutant, which exhibits reduced interactions with RIPK3, resulted in more pronounced apoptosis than infection with WT NSs, suggesting that NSs not only drives necroptosis but also concomitantly suppresses apoptosis. Although the precise molecular mechanisms remain to be clarified, such dual regulation of cell death pathways implies that NSs contributes to immune evasion by shifting host responses away from apoptosis while favoring necroptotic signaling. In the context of vaccination, the anti-apoptotic property of the ΔNSs virus may prolong antigen presentation and facilitate adaptive immune priming, as demonstrated in the present study. Taken together, our findings support a model in which SFTSV strategically modulates both antigen presentation and programmed cell death to evade innate immunity while preserving features advantageous for vaccine-induced immune activation.

Importantly, immunization with SFTSV ΔNSs-GFP provided complete protection against lethal SFTSV challenge from genotypes B, D, and F, with durable immunity lasting up to 12 months (Figure 6). Passive serum transfer experiments confirmed that long-lived neutralizing antibodies contributed to protection. However, sera alone did not prevent mortality at higher challenge doses (10^3 FFUs), providing only a significant delay in death, while partial protection with enhanced survival (60%) was observed at lower doses (10^1 FFUs). These findings suggest that while neutralizing antibodies play an important role in limiting viral entry, they may not be solely adequate for complete protection. Rather, sustained antigen-specific T cell responses, detected up to 3 months post-immunization in IFNAR-KO mice (Figure S7), likely provide complementary contributions to comprehensive immunity. Collectively, these results underscore the importance of eliciting both humoral and cellular responses in the development of effective SFTS vaccine strategies.

Our findings highlight the strong immunogenicity and protective efficacy of the SFTSV ΔNSs platform, demonstrating its potential as a versatile bivalent vaccine vector. Importantly, however, we recognize that rigorous evaluation of safety remains essential for future translation. WT SFTSV causes substantial morbidity and mortality in humans, and therefore, even attenuated live derivatives must be approached with caution. In our study, ΔNSs infection produced only transient and mild hematologic changes in mice and non-human primates, without sustained viremia, and histopathological analyses revealed no evidence of systemic inflammation or organ damage. Consistent attenuation was also reported in aged ferrets infected with the ΔNSs virus.[28] While these observations suggest a favorable safety margin relative to the WT virus, they do not constitute a comprehensive safety profile.

Future work will need to systematically address this limitation through expanded preclinical studies, including dose escalation, long-term monitoring, and evaluation in immunocompromised and aged models. Such efforts will be required to fully define the safety parameters of the ΔNSs vector and determine its suitability for clinical application. Thus, while our present findings support the feasibility of the ΔNSs vaccine strategy, they should be interpreted as a proof-of-concept foundation rather than a definitive demonstration of clinical readiness.

The inoculation route of an attenuated viral vaccine can influence viral dissemination, reactogenicity, and the efficacy of antigen-specific adaptive immunity.[94] A previous study demonstrated that WT SFTSV exhibited no differences in viral dissemination, morbidity, or mortality in IFNAR-KO mice, regardless of whether inoculation was performed subcutaneously or intramuscularly.[95] In contrast, our preliminary data indicate that the ΔNSs virus disseminated more rapidly into draining lymph nodes and elicited stronger antibody responses when delivered intramuscularly than when administered subcutaneously in WT mice (Figure S8). Consistent with this, a recent review concluded that intramuscular injection generally induces more robust immune responses and fewer local adverse events compared to subcutaneous administration.[94] These findings suggest that intramuscular delivery of the ΔNSs virus may represent a preferable route for achieving enhanced immunity with reduced local reactogenicity, although more extensive preclinical and clinical studies are warranted.

Taken together, these data indicate that the ΔNSs virus elicits immune activation without overt tissue damage, supporting a favorable preclinical safety profile. Nevertheless, potential immunosuppression, long-term safety, and efficacy in aged or immunocompromised hosts should be evaluated in future studies. Head-to-head comparisons with conventional vaccine platforms (e.g., subunit or mRNA vaccines) will also be critical to delineate the distinct advantages and limitations of the SFTSV ΔNSs vector.

Collectively, our findings provide a strong preclinical foundation for developing SFTSV ΔNSs as both an effective live-attenuated vaccine for SFTSV and a flexible bivalent vaccine vector. Immunization with the ΔNSs virus conferred complete and durable protection across multiple SFTSV genotypes, with immunity sustained for up to 12 months in the mouse model. Moreover, robust humoral and cellular immune responses were confirmed in non-human primates, underscoring its translational potential. Beyond its efficacy as an SFTSV vaccine, the ΔNS platform's ability to express heterologous antigens supports its broader application as a bivalent vaccine, offering protective T cell-mediated immunity against intracellular bacterial infections and tumors. These combined attributes position the SFTSV ΔNS vector as a promising next-generation vaccine platform with broad applicability and significant clinical potential.

MATERIALS AND METHODS

Cells culture

BSR-T7/5, Vero E6, and L929 cells were maintained in Dulbecco's modified Eagle's medium (DMEM; Welgene #LM001-05), supplemented with 10% fetal bovine serum (FBS; Gibco #12483020) and 1% penicillin/streptomycin (P/S; Gibco #15140122). OVA-expressing melanoma B16MO5 cells were maintained in RPMI, supplemented with 10% FBS and 1% PS. Cells were incubated at 37°C in a humidified incubator with 5% CO_2.

Plasmid construction

To generate the pTVT7-HB29SΔNSs construct, the NSs gene was deleted by PCR, while retaining the terminal 12 amino acids (36 nucleotides) at the 3′ end that are required for proper transcription termination,[96] and an EcoRI recognition site was introduced. For gene insertion, the NP gene segment containing the XhoI site was designated as part 1, while EGFP, OVA, and TSA56 genes were individually amplified using primers incorporating an EcoRI sequence, forming part 2. These two fragments were designed with an overlapping region of approximately six amino acids (18 nucleotides) to facilitate seamless fusion. Subsequently, overlapping PCR was performed to assemble part 1 and part 2 into a single continuous sequence. The final construct was cloned into pTVT7-HB29SdelNSs following digestion with XhoI and EcoRI. Plasmid transformation was carried out in *E. coli*, followed by culture in Luria broth supplemented with ampicillin. Plasmids were extracted using a Qiagen Plasmid Maxiprep Kit (Qiagen #CMP112) for downstream applications.

Virus propagation and recombinant virus generation

The SFTSV strain 2015-JJ01 (JJ strain; NCBI GenBank: MN329148–MN329150) and recombinant viruses were propagated in Vero E6 cells (ATCC #CRL-1586). The reverse genetics system for SFTSV was described in a previous study.[31] To generate recombinant viruses expressing different target proteins, various plasmid constructs, including those encoding EGFP, OVA, and TSA56, were utilized. Specifically, pTVT7-HB29S constructs carrying these inserts were transfected into BSR-T7/5 cells along with four additional helper plasmids to facilitate viral genome rescue. Briefly, 7.5×10^5 BSR-T7/5 cells were transfected with a plasmid mix containing 0.1 μg pTM1-HB29ppL; 0.5 μg pTM1-HB29N; 1 μg of pTVT7-HB29S-EGFP, pTVT7-HB29S-OVA, or pTVT7-HB29S-TSA56; 1 μg pTVT7-HB29M; and 1 μg pTVT7-HB29ppL using TransIT-LT1 (Mirus #MIR2304) at a ratio of 3 μL per μg of DNA. After 5 days, virus-containing supernatants were harvested, further amplified in Vero E6 cells, and stored at −80°C. Reassortant viruses were generated using the plaque purification method as previously described.[32] In brief, a monolayer of Vero E6 cells was coinfected with both a recombinant virus derived from the HB29 strain and the JJ strain at a multiplicity of infection (MOI) of 10 for each virus simultaneously. The viral inoculum was added to the cellular monolayer in a T-75 flask and incubated at 37°C for 90 min. After washing, the cells were cultured in 10 mL of DMEM with 2% FBS and incubated at 37°C with 5% CO_2 for 4 days. Following incubation, the supernatant was serially diluted and transferred to a healthy Vero E6 monolayer for plaque assay. At 9 days post-infection, well-separated SFTSV plaques were picked with a pipette, transferred to a microfuge tube containing 100 μL of serum-free DMEM, and then propagated in Vero E6 cells. The isolated viral clones were amplified using an RT-PCR kit (Accupower Taq PCR PreMix; Bioneer) with specific primer sets[32] targeting L, M, and S segments for sequence analysis. Representative sequencing results of the reassortant viruses without any contamination are presented in Figure S9.

Focus-forming assay for viral quantification

The FFUs of SFTSV were determined using a focus-forming assay with methylcellulose overlay media. Briefly, viral supernatants were filtered and serially diluted in 10-fold increments before being applied to a monolayer of Vero E6 cells. Following a 2-h incubation at 37°C, the supernatants were removed by suction, and cells were overlaid with DMEM supplemented with 2% FBS, 1% P/S, and 0.8% (w/v) methylcellulose. The cultures were incubated at 37°C for 7 days. Cells were subsequently fixed and permeabilized with a 1:1 methanol-acetone solution for 20 min at room temperature (RT) under UV irradiation. SFTSV foci were detected using a 1:2,000 dilution of a rabbit anti-SFTSV NP polyclonal antibody, followed by a 1:5,000 dilution of a goat anti-rabbit immunoglobulin (Ig)G secondary antibody conjugated to horseradish peroxidase (HRP) (Invitrogen #31460). Viral foci were visualized using 3,3′-diaminobenzidine (DAB) substrate (Merck #D5637).

Bacterial culture and purification

O. tsutsugamushi strain Boryong (GenBank: AM494475) was semi-purified as previously described.[97] When more than 90% of L929 cells were infected, as determined by indirect immunofluorescence antibody staining, the cells were collected, homogenized using a glass Dounce homogenizer (Wheaton), and centrifuged at 500 × *g* for 5 min. The supernatant was collected and stored in liquid nitrogen until further use.

Western blot analysis

Immunoblotting was performed on Vero E6 cells infected with recombinant viruses at an MOI of 1. After 48 h post-infection, cells

were lysed using 1% Nonidet P-40 buffer. Total protein concentration was determined using the BCA protein assay to ensure equal protein loading. Lysates were subjected to sodium dodecyl sulfate-polyacrylamide gel electrophoresis (SDS-PAGE) and transferred onto a polyvinylidene difluoride (PVDF) membrane (Merck Millipore #IPVH00010) using a semi-dry transfer system (Bio-Rad) at 20 V for 30 min. Membranes were blocked with 5% (w/v) skim milk in PBS supplemented with 0.05% (v/v) Tween 20 for 1 h before incubation with primary antibodies. The following primary antibodies were used: rabbit anti-SFTSV NP polyclonal antibody (1:2,000 dilution),[32] mouse anti-OVA monoclonal antibody (1:5,000 dilution; Abcam #ab181688), mouse anti-GFP monoclonal antibody (1:2,000 dilution; Santa Cruz #sc-8334), and mouse anti-β-actin antibody (1:5,000 dilution; Santa Cruz #sc-47778). Membranes were then incubated with appropriate HRP-conjugated secondary antibodies at RT for 1 h. Bands were developed using WESTSAVE western blot detection reagent (AbFrontier #LF-QC0103) and visualized with an LAS-4000 imaging system (Fuji).

Confocal microscopy

Vero E6 cells were infected at an MOI of 1 and incubated for 2 h. At 24 h post-infection, cells were transferred onto coverslips and incubated for an additional 24 h. Cells were then fixed with 4% paraformaldehyde (PFA) for 10 min and permeabilized with 0.2% Triton X-100 for 15 min at RT. For immunostaining, cells were incubated with a rabbit anti-SFTSV NP polyclonal antibody (1:1,000 dilution) for 1 h, followed by incubation with an Alexa 594-conjugated anti-rabbit IgG secondary antibody (1:2,000 dilution; Invitrogen #21207) for 1 h. Nuclei were counterstained with DAPI (Thermo Fisher Scientific #PI62247) for 10 min. Slides were mounted using a mounting medium (Vectashield #H-1000), and confocal images were acquired using an Olympus FV1000 confocal microscope. Image processing and analysis were performed using dedicated software.

Primary cell isolation and differentiation

Bone marrow cells were isolated from Rag2 KO (The Jackson Laboratory, #008449) or IFNAR-KO[98] (C57BL/6 background) mice by dissecting the femurs and tibiae, severing at the joints, and flushing the marrow with PBS through a 23G needle. The collected cell suspension was filtered through a 70-μm cell strainer, centrifuged, and washed multiple times with PBS. For BMDC differentiation, bone marrow cells (1×10^6 cells/well) were seeded in 24-well plates and cultured in Iscove's modified Eagle's medium (IMDM; Gibco #12440-053) supplemented with 10% FBS, 1.5 ng/mL recombinant mouse granulocyte-macrophage colony-stimulating factor (GM-CSF) (PeproTech), 1.5 ng/mL recombinant mouse interleukin (IL)-4 (PeproTech), 1% P/S, 50 μg/mL gentamicin (Gibco #15750060), 2 mM L-glutamine (Gibco #25060-081), and 50 nM β-mercaptoethanol (Gibco #21985023). The medium was replaced every other day for 7 days, and immature BMDCs were collected for subsequent experiments. For BMDM differentiation, bone marrow cells were resuspended in DMEM supplemented with 20% macrophage CSF (M-CSF)-derived L929 cell supernatants, 10% FBS, and 1% P/S and plated in 10-cm dishes (5×10^6 cells per plate). On day 3, fresh medium containing 20% L929 supernatants was added. BMDMs were fully differentiated and ready for use after 7 days of culture. The differentiation of BMDCs and BMDMs was confirmed via flow cytometric analysis (Figure S10A).

Preparation of splenocytes

Spleens were mechanically dissociated using the flat end of a syringe plunger. The collected cell suspension was filtered through a 70-μm cell strainer, red blood cells (RBCs) were lysed using RBC lysis buffer (Sigma #R7757), and the reaction was stopped by adding RPMI 1640 supplemented with 10% FBS and 1% P/S. Cells were centrifuged at $500 \times g$ at 4°C for 5 min, and the supernatant was removed. The resulting cell pellet was resuspended in RPMI supplemented with 10% FBS and 1% P/S media and passed through a 70-μm strainer. Prepared splenocytes were used for downstream experiments.

Quantification of viral and bacterial load by RT-qPCR

The viral load in infected cells and mouse organs was quantified using quantitative real-time PCR (real-time qPCR). Total RNA was extracted using Trizol (Life Technology #15596018) or Trizol LS reagent (Life Technology #10296028), followed by reverse transcription into cDNA using the HiSenScript RH (−) RT Premix kit (Intron #25087). The cDNA was then subjected to real-time qPCR using the SensiFAST Probe Lo-ROX kit (Bioline #BIO-84020) with specific primers and a detection probe targeting the NP gene of SFTSV (forward: 5′-CCTTCAGGTCATGACAGCTGG-3′, reverse: 5′-ACCAGGCTCTCAATCACTCCTGT-3′, probe: 5′-6FAM-AGC ACATGTCCAAGTGGGAAGGCTCTG-BHQ1-3′). The lower limit of detection (LOD) for RT-qPCR was 10^2 copies/reaction; values below the LOD were considered undetectable (n.d.). For bacterial load quantification, DNA was extracted from spleen and lung tissues using the Qiagen DNA extraction kit (Qiagen #69506) according to the manufacturer's instructions. Specific primers were designed to detect the 47-kDa gene of *O. tsutsugamushi* in the bacterial genome (forward: 5′-AACTGATTTTATTCAAACTAATGCTGCT-3′, reverse: 5′-TATGCCTGAGT AAGATACATGAATGGAATT-3′, probe: 5′-FAM-GGGTAGCTTTGGTGGACCGATGTTT AATCT-BHQ1-3′). qPCR was performed using the Bio-Rad CFX Connect Real-Time PCR System.

Analysis of APCs

BMDCs, BMDMs, and B cells were infected with SFTSV at an MOI of 1 and incubated for 24 h. For activation marker analysis, LPS (1 μg/mL) (Sigma-Aldrich #LPS25) was used as a positive control. The expression of co-stimulatory molecules and activation markers was analyzed by flow cytometry. Cells were stained with fluorophore-conjugated antibodies, including anti-CD40 PE (clone 3/23, Pharmingen #553791), anti-CD80 APC (clone 16-10A1, Biolegend #104714), anti-CD86 BV605 (clone IT2.2, BioLegend #305428), anti-MHC class I FITC or Percp-Cy5.5 (clone AF6-88.5, BioLegend #116506), anti-MHC II Pacific Blue (clone AF6-120.1, BioLegend #116422), and anti-MHC I:SIINFEKL APC/Fire 750 (clone 25-D1.16, BioLegend #141613), for the analysis of co-stimulatory

molecule and activation marker expression. For apoptosis analysis, BMDCs, BMDMs, and B cells were infected with recombinant SFTSV or WT virus at an MOI of 1 and incubated for 24 or 48 h. Cells were stained with Annexin V (BioLegend #640920) in Annexin V binding buffer (140 mM NaCl and 2.5 mM $CaCl_2$) for 15 min at RT in the dark, followed by the direct addition of 7-AAD (Pharmingen #559925). After 10 min of incubation, the cells were washed and analyzed by flow cytometry.

Transcriptome analysis

BMDCs were mock infected or infected with recombinant SFTSV-ΔNSs-GFP and sorted into GFP^- and GFP^+ populations at 24 h post-infection. Total RNA was isolated using TRIzol reagent (Invitrogen). RNA integrity (RIN > 7.0) and concentration were assessed with an Agilent 4200 TapeStation System (Agilent Technologies) and Qubit fluorometer (Thermo Fisher Scientific). Libraries were prepared with the QuantSeq 3′mRNA-Seq v.2 kit (Lexogen) and sequenced on an Illumina NextSeq 2000 platform to generate 100-bp single-end reads. Raw reads were trimmed using fastp (v.0.23.1) and aligned to the *Mus musculus* reference genome (GRCm39) with STAR (v.2.7.10b). Gene-level counts were obtained using featureCounts (v.2.0.6). Differential expression analysis was performed with edgeR (v.3.42.4) using Trimmed Mean of M-values (TMM) normalization. Genes with a $|\log_2 \text{fold change}| > 1$ and a false discovery rate (FDR) < 0.05 were considered differentially expressed. Heatmaps were generated using pheatmap (v.1.0.12), and Gene Ontology (GO) enrichment analysis was conducted with clusterProfiler (v.4.6.2), applying Benjamini-Hochberg correction (FDR < 0.05). Protein-protein interaction (PPI) networks were generated from DEGs (GFP^+ versus GFP^-) using STRING (v.12.0) and visualized in Cytoscape (v.3.10.3).

Mouse study

All animal experiments were reviewed and approved by Seoul National University and were conducted in compliance with ethical regulations for animal testing and research. *Ifnar1*$^{-/-}$ mice (C57BL/6 background)[98] were housed in a clean facility, and breeding pairs and their pups were maintained under controlled conditions. WT C57BL/6 mice were purchased at 6 weeks of age, acclimated for 1 week, and subsequently used for experiments. For SFTSV infection, 7- to 9-week-old mice were injected subcutaneously in the dorsal neck region or intramuscularly in the thigh muscles of the hindlimb with the indicated dose of SFTSV in PBS on day 0. For *O. tsutsugamushi* infection, mice were injected intravenously with 2×10^5 FFUs. For OVA protein immunization, mice were intramuscularly immunized with 10 μg of OVA protein mixed with aluminum hydroxide (Alum) adjuvant at a 9:1 ratio (OVA solution: 2% Alhydrogel in PBS; InvivoGen, #vac-alu-250), administered at 2-week intervals for a total of three doses. For cancer cell inoculation, 5×10^5 B16MO5 cells were injected subcutaneously. The body weight of infected mice was recorded daily. Blood samples were collected via retro-orbital bleeding using heparin-coated tubes at the designated time points and centrifuged at $10{,}000 \times g$ for 10 min. The obtained serum was collected and stored at −80°C until further analysis. For adoptive transfer experiments, 150 μL of pooled serum was injected intravenously into each mouse. The following day, mice were subcutaneously infected with SFTSV.

Non-human primate study

Rhesus macaque (*Macaca mulatta*) were used in this study. All animals were female, aged 7 to 9 years, and housed at the Korea Research Institute of Bioscience and Biotechnology (KRIBB) under conditions compliant with relevant ethical and animal care guidelines. For infection, animals were intramuscularly injected in the thigh muscles of the hindlimb with 10^6 FFUs of SFTSV. Blood samples collected 2 weeks before the first immunization were used as controls, and body weight and temperature were monitored until 10 weeks after the initial immunization. PBMCs were isolated from heparinized whole blood using a Lymphoprep (STEMCELL Technologies, #18061) gradient, following the manufacturer's protocol. Serum was collected in EDTA-coated tubes, and complete blood count (CBC; Mindray, BC-5000) and serum biochemistry (Fujifilm, DRI-CHEM NX700) were measured on the same day. The remaining serum samples were stored at −80°C for subsequent analysis.

Production of recombinant proteins

The SFTS *NP* or *O. tsutsugamushi TSA56* gene, cloned into Novagen's pET-based expression vector (Sigma-Aldrich #69864), was transformed into *E. coli* BL21 cells. The transformed cells were plated on Luria-Bertani (LB) agar containing ampicillin. Colonies were picked and cultured in LB medium supplemented with ampicillin (100 μg/mL) at 37°C until the optical density (OD_{600}) reached 0.6. Protein expression was then induced with 0.01 mM IPTG (Duchdfa #367-93-1), and the culture was incubated at 16°C for 16 h. Cells were harvested by centrifugation at $8{,}000 \times g$ for 15 min at 4°C. After removing the supernatant, the cell pellet was resuspended in lysis buffer (1 mg/mL lysozyme; Bioworld #21560016-3, 1× protease inhibitor; Thermo Fisher Scientific #78430 in binding buffer), where the binding buffer consisted of 300 mM sodium chloride and 50 mM sodium phosphate in 1 L of distilled water. The resuspended cells were incubated at 4°C for 10 min and then sonicated for 20 min with 10-s on/off cycles. The lysate was clarified by centrifugation at $22{,}000 \times g$ for 50 min at 4°C, and the resulting supernatant was applied to Ni-NTA resin (Qiagen #30230) for affinity purification. After binding, the resin was washed with binding buffer containing imidazole, and the target protein was eluted using elution buffer (250 mM imidazole in binding buffer). The eluted recombinant protein was dialyzed against PBS and stored at −80°C.

Enzyme-linked immunosorbent assay

Enzyme-linked immunosorbent assay (ELISA) plates (Thermo Fisher Scientific #439454) were coated with 100 μL of 1 μg/mL recombinant SFTSV NP or Gn (eEnzyme #PV-GP-40P) in PBS and incubated overnight at 4°C. The plates were washed three times with wash buffer (PBS +0.05% [v/v] Tween 20) and blocked with assay buffer (5% [w/v] skim milk in wash buffer) for 2 h at RT. Serum samples from each animal were serially diluted in a 4-fold series across 8–10 points, starting at a 1:100 dilution. 100 μL of the diluted

samples was added to each well and incubated for 1 h at RT. Sera collected prior to immunization were used as negative controls to establish the cutoff titer, following the method previously described (mean OD ± f × SD at a 1:100 dilution), where f corresponds to the standard deviation multiplier (e.g., 5.077 for $n = 4$ in 99% confidence level) used to define the confidence threshold based on sample size.[99] For detection, HRP-conjugated secondary antibodies were used: goat anti-mouse IgG (Thermo Fisher Scientific #31430) or goat anti-monkey IgG (NOVUS #NB7215). After incubation, 3,3′,5,5′-tetramethylbenzidine (TMB) peroxidase substrate solution (BioLegend #421101) was added, and color development was allowed to proceed for 7 min. The reaction was stopped by adding 1 M H_3PO_4, and the absorbance was measured at 450 nm using a TECAN microplate reader.

SFTSV focus reduction neutralization test

Serum samples were heat inactivated at 56°C for 30 min and serially diluted 4-fold across five points (ranging from 1:40 to 1:10,240). Each serum dilution was mixed in equal volume with SFTSV solution containing 100 FFUs and incubated at 4°C for 1 h. The mixture was then applied to a monolayer of Vero E6 cells cultured in a 24-well plate (TTP #92006). The subsequent steps, including viral focus visualization, were performed as described in the FFU assay. The percentage of focus reduction was calculated using Equation 1:

$$\text{focus reduction } (\%) = ((\text{no. of plaques without serum}) - (\text{no. of plaques with serum})) / (\text{no. of plaques without serum}) \times 100. \quad \text{(Equation 1)}$$

All experiments were performed in duplicate, and focus reduction neutralization test $(FRNT)_{50}$ values were determined using nonlinear regression analysis (log[inhibitor] versus normalized response) in GraphPad Prism Software v.8.0. Negative control sera were included in each assay to establish the LOD under experimental conditions.

Flow cytometry

Cells were washed twice with fluorescence-activated cell sorting (FACS) buffer (PBS +3% FBS) and stained with Zombie Live/Dead dye (BioLegend #423102) for 20 min at 4°C, followed by two additional washes with FACS buffer. Cells were then blocked with blocking buffer (FACS buffer containing 10% mouse, goat, and rat serum and 10 μg/μL of FcR Block:2.4G2 BD Pharmingen #553142) for 30 min at 4°C. For tetramer staining, cells were incubated with fluorophore-conjugated antibodies against anti-CD3 Pacific Blue (clone 17-A2, BioLegend #100214), anti-CD4 Percp (clone RM4-5, BioLegend #100538), anti-CD8 FITC (clone 53-6.7, BioLegend #100706), Tetramer I APC (H-2K(b) chicken OVA 257-264 SIINFEKL; NIH core facility), and Tetramer II PE (I-A(b) chicken OVA 329-337 AAHAEINEA; NIH core facility) for 20 min at 4°C. For intracellular cytokine staining, 2×10^6 cells per well were stimulated overnight with 10 μg of either SFTSV NP or *O. tsutsugamushi* TSA56 protein. After 12 h, brefeldin A (BD #555029) was added, followed by an additional 4 h of incubation. Cells were then subjected to Live/Dead staining and subsequently stained for surface markers, including anti-CD3 Pacific Blue, anti-CD4 Percp, anti-CD8 FITC, anti-CD44 APC Fire/750 (clone NIM-R8, BioLegend #156004), and anti-CD62L BV605 (clone MEL-14, BD Horizon #563252), for 20 min at 4°C. After surface staining, cells were fixed and permeabilized using the Cytofix/Cytoperm Fixation/Permeabilization Solution Kit (BD Biosciences #554714), followed by intracellular staining with anti-IFN-γ APC (clone XMG1.2, BD Pharmingen #554413) and anti-TNF-α PE (clone MP6-XT22, Invitrogen #12-7321-81) for 30 min at 4°C. For immune cell phenotyping, cells were stained with antibodies against anti-CD45 FITC (clone 30-F11, BioLegend #103107) or APC Fire/750 (clone 30-F11, BioLegend #103153), anti-CD11b FITC (clone M1/70, BioLegend #101205), anti-F4/80 PE-Cy7 (clone BM8, BioLegend #123114), anti-IA/IE Percp (clone M5/114.15.2, BioLegend #107623), anti-CD19 PE (clone 1D3, eBioscience #12-0193-83), and anti-CD3 Pacific Blue. Flow cytometry data were acquired using CytoFLEX S (Beckman Coulter) and analyzed with FlowJo software. The gating strategies are shown in Figure S10B.

ELISpot assay

PBMCs were resuspended in RPMI 1640 supplemented with 10% FBS, 1% P/S, 1× non-essential amino acids (NEAAs, Gibco #11140050), and 50 nM β-mercaptoethanol and then counted. The monkey IFN-γ ELISpot kit (Mabtech #3421M-4AST-2) was used to quantify IFN-γ-producing T cells, following the manufacturer's protocol. Briefly, precoated plates were blocked with resuspension medium at RT for 30 min. 200 μL of 1×10^6 PBMCs/mL was added to each well. For stimulation, 200 μL of media only (negative control), SFTSV NP pooled peptides (each peptide at 1 mg/mL), or 1 mg/mL PMA/ionomycin (positive control) was added to the wells, followed by incubation for 24 h at 37°C in 5% CO_2. Plates were developed according to the manufacturer's instructions and analyzed using the AID ELISpot Reader (Autoimmun Diagnostika).

Histopathology analysis

Liver and spleen tissues were collected from IFNAR-KO mice at day 3 post-infection and fixed in 4% PFA overnight at 4°C. Samples were embedded in paraffin, sectioned at 4–5 μm, and stained with hematoxylin and eosin (H&E) using standard protocols. Whole-slide images were acquired under bright-field microscopy and imported into QuPath (v.0.6.0) for viewing. Representative images were selected to illustrate characteristic pathological changes, including vascular congestion in the liver and white pulp atrophy in the spleen.

Statistical analysis

Statistical analyses of different groups were performed using a two-tailed Mann-Whitney test or nonparametric one-way analysis of variance, followed by the Kruskal-Wallis test for multiple comparisons. Survival curves were generated by the Kaplan-Meier method and compared using the log rank (Mantel-Cox) test.

Correlations between variables were assessed using Spearman's correlation test. $p < 0.05$ was considered statistically significant. All data analyses were performed using the GraphPad Prism Software v.8 (GraphPad software).

DATA AND CODE AVAILABILITY

The data and materials that support the findings of this study are available from the corresponding author (N.-H.C.) upon reasonable request.

ACKNOWLEDGMENTS

This work was supported by a grant from the Korea Health Technology R&D Project through the Korea Health Industry Development Institute, funded by the Ministry of Health and Welfare (HV22C0009); the Korea-US Collaborative Research Project, funded by the Ministry of Science, ICT and Future Planning and the Ministry of Health and Welfare of Korea (RS-2024-00467046); the Korea Research Institute of Bioscience and Biotechnology (KRIBB) Research Initiative Programs (grant number KGM4572532); and the National Institutes of Health (AI152190 and AI171201). H.-J.R., Y.L., K.J., J.K., N.-Y.J., S.-H.L., and S.-Y.K. received a scholarship from the BK21-plus education program provided by the National Research Foundation of Korea. All experiments involving live, WT SFTSV were conducted by certified personnel in biosafety level 3 (BSL-3) laboratories for cell culture and animal biosafety level 3 (ABSL-3) laboratories for animal studies. Experiments using live NS-deleted SFTSV were performed in BSL-2 laboratories for cell culture and ABSL-2 laboratories for animal studies, following biosafety guidelines. The non-human primate study was exceptionally conducted in ABSL-3 laboratories, in compliance with biosafety regulations. All animal experiments were approved by the institutional animal care and use committee (IACUC) of Seoul National University (approval number: IACUC- SNU-230216-4-1) and the KRIBB (approval numbers: IACUC-KRIBB-AEC-23149 and 24006).

AUTHOR CONTRIBUTIONS

N.-H.C. conceived and conceptualized the study and designed the experiments. H.-J.R., Y.L., K.J., J.K., N.-Y.J., S.-H.L., and S.-Y.K. performed the *in vitro* experiments. H.-J.R., Y.L., Yujin Kim, S.H.B., G.K., J.-G.K., and J.J.H. performed the *in vivo* studies, supported by N.-Y.J., S.-H.L., S.-Y.K., Y.-J.K., N.-Y.H., and Yuri Kim H.-J.R., Y.L., Y.K.C., J.U.J., J.J.H., and N.-H.C performed data analysis and drafted the manuscript. All authors edited and approved the final paper.

DECLARATION OF INTERESTS

N.-H.C., H.-J.R., and Y.L. are co-inventors on a patent based on the recombinant bandavirus vector described in this manuscript.

DECLARATION OF GENERATIVE AI AND AI-ASSISTED TECHNOLOGIES IN THE WRITING PROCESS

During the preparation of this work, the authors used ChatGPT in order to enhance readability and improve English grammar. After using this tool/service, the authors reviewed and edited the content as needed and take full responsibility for the content of the publication.

SUPPLEMENTAL INFORMATION

Supplemental information can be found online at https://doi.org/10.1016/j.ymthe.2025.10.044.

REFERENCES

1. Casel, M.A., Park, S.J., and Choi, Y.K. (2021). Severe fever with thrombocytopenia syndrome virus: emerging novel phlebovirus and their control strategy. Exp. Mol. Med. *53*, 713–722.
2. Yu, X.-J., Liang, M.-F., Zhang, S.-Y., Liu, Y., Li, J.-D., Sun, Y.-L., Zhang, L., Zhang, Q.-F., Popov, V.L., Li, C., et al. (2011). Fever with thrombocytopenia associated with a novel bunyavirus in China. N. Engl. J. Med. *364*, 1523–1532.
3. Takahashi, T., Maeda, K., Suzuki, T., Ishido, A., Shigeoka, T., Tominaga, T., Kamei, T., Honda, M., Ninomiya, D., Sakai, T., et al. (2014). The first identification and retrospective study of severe fever with thrombocytopenia syndrome in Japan. J. Infect. Dis. *209*, 816–827.
4. Tran, X.C., Yun, Y., Van An, L., Kim, S.H., Thao, N.T.P., Man, P.K.C., Yoo, J.R., Heo, S.T., Cho, N.H., and Lee, K.H. (2019). Endemic severe fever with thrombocytopenia syndrome, Vietnam. Emerg. Infect. Dis. *25*, 1029–1031.
5. Kim, K.H., Yi, J., Kim, G., Choi, S.J., Jun, K.I., Kim, N.H., Choe, P.G., Kim, N.J., Lee, J.K., and Oh, M.D. (2013). Severe fever with thrombocytopenia syndrome, South Korea, 2012. Emerg. Infect. Dis. *19*, 1892–1894. https://doi.org/10.3201/eid1911.130792.
6. Yun, Y., Heo, S.T., Kim, G., Hewson, R., Kim, H., Park, D., Cho, N.H., Oh, W.S., Ryu, S.Y., Kwon, K.T., et al. (2015). Phylogenetic Analysis of Severe Fever with Thrombocytopenia Syndrome Virus in South Korea and Migratory Bird Routes Between China, South Korea, and Japan. Am. J. Trop. Med. Hyg. *93*, 468–474. https://doi.org/10.4269/ajtmh.15-0047.
7. Win, A.M., Nguyen, Y.T.H., Kim, Y., Ha, N.Y., Kang, J.G., Kim, H., San, B., Kyaw, O., Htike, W.W., Choi, D.O., et al. (2020). Genotypic Heterogeneity of Orientia tsutsugamushi in Scrub Typhus Patients and Thrombocytopenia Syndrome Co-infection, Myanmar. Emerg. Infect. Dis. *26*, 1878–1881. https://doi.org/10.3201/eid2608.200135.
8. Zohaib, A., Zhang, J., Saqib, M., Athar, M.A., Hussain, M.H., Chen, J., Sial, A.U.R., Tayyab, M.H., Batool, M., Khan, S., et al. (2020). Serologic Evidence of Severe Fever with Thrombocytopenia Syndrome Virus and Related Viruses in Pakistan. Emerg. Infect. Dis. *26*, 1513–1516. https://doi.org/10.3201/eid2607.190611.
9. Rattanakomol, P., Khongwichit, S., Linsuwanon, P., Lee, K.H., Vongpunsawad, S., and Poovorawan, Y. (2022). Severe Fever with Thrombocytopenia Syndrome Virus Infection, Thailand, 2019-2020. Emerg. Infect. Dis. *28*, 2572–2574. https://doi.org/10.3201/eid2812.221183.
10. Thi Hai Yen, N., Kim, C., Jeong, S., Jeon, K., Choi, H., Ro, H.J., Kim, H.I., Kim, Y., Kang, J.G., Park, D., et al. (2019). Severe Fever with Thrombocytopenia Syndrome Virus Infection or Mixed Infection with Scrub Typhus in South Korea in 2000-2003. Am. J. Trop. Med. Hyg. *101*, 1096–1099. https://doi.org/10.4269/ajtmh.19-0392.
11. He, Z., Wang, B., Li, Y., Du, Y., Ma, H., Li, X., Guo, W., Xu, B., and Huang, X. (2020). Severe fever with thrombocytopenia syndrome: a systematic review and meta-analysis of epidemiology, clinical signs, routine laboratory diagnosis, risk factors, and outcomes. BMC Infect. Dis. *20*, 575.
12. Saijo, M. (2018). Pathophysiology of severe fever with thrombocytopenia syndrome and development of specific antiviral therapy. J. Infect. Chemother. *24*, 773–781.
13. Woo, D., Michelow, I.C., Choi, Y., Lee, H., and Park, S. (2025). Transmission of Severe Fever with Thrombocytopenia Syndrome (SFTS) to humans: A systematic review of individual participant data and meta-analysis. J. Infect. Public Health *18*, 102685. https://doi.org/10.1016/j.jiph.2025.102685.
14. Anonymous (2018). Annual Review of Diseases Prioritized under the Research and Development Blueprint (World Health Organization Informal consultation).
15. National Institute of Allergy and Infectious Diseases (NIAID) (2003). NIAID Biodefense Research Agenda for Category B and C Priority Pathogens. NIH Pub. No. *03-5315*. https://downloads.regulations.gov/FDA-2008-N-0567-0020/content.pdf.
16. Tani, H., Shimojima, M., Fukushi, S., Yoshikawa, T., Fukuma, A., Taniguchi, S., Morikawa, S., and Saijo, M. (2016). Characterization of glycoprotein-mediated entry of severe fever with thrombocytopenia syndrome virus. J. Virol. *90*, 5292–5301.
17. Halldorsson, S., Behrens, A.-J., Harlos, K., Huiskonen, J.T., Elliott, R.M., Crispin, M., Brennan, B., and Bowden, T.A. (2016). Structure of a phleboviral envelope glycoprotein reveals a consolidated model of membrane fusion. Proc. Natl. Acad. Sci. USA *113*, 7154–7159.
18. Plegge, T., Hofmann-Winkler, H., Spiegel, M., and Pöhlmann, S. (2016). Evidence that processing of the severe fever with thrombocytopenia syndrome virus Gn/Gc polyprotein is critical for viral infectivity and requires an internal Gc signal peptide. PLoS One *11*, e0166013.
19. Zhou, H., Sun, Y., Wang, Y., Liu, M., Liu, C., Wang, W., Liu, X., Li, L., Deng, F., Wang, H., et al. (2013). The nucleoprotein of severe fever with thrombocytopenia syndrome virus processes a stable hexameric ring to facilitate RNA encapsidation. Protein Cell *4*, 445–455.

20. Santiago, F.W., Covaleda, L.M., Sanchez-Aparicio, M.T., Silvas, J.A., Diaz-Vizarreta, A.C., Patel, J.R., Popov, V., Yu, X.-j., García-Sastre, A., and Aguilar, P.V. (2014). Hijacking of RIG-I signaling proteins into virus-induced cytoplasmic structures correlates with the inhibition of type I interferon responses. J. Virol. *88*, 4572–4585.

21. Ning, Y.-J., Feng, K., Min, Y.-Q., Cao, W.-C., Wang, M., Deng, F., Hu, Z., and Wang, H. (2015). Disruption of type I interferon signaling by the nonstructural protein of severe fever with thrombocytopenia syndrome virus via the hijacking of STAT2 and STAT1 into inclusion bodies. J. Virol. *89*, 4227–4236.

22. Ning, Y.-J., Wang, M., Deng, M., Shen, S., Liu, W., Cao, W.-C., Deng, F., Wang, Y.-Y., Hu, Z., and Wang, H. (2014). Viral suppression of innate immunity via spatial isolation of TBK1/IKKε from mitochondrial antiviral platform. J. Mol. Cell Biol. *6*, 324–337.

23. Wu, X., Qi, X., Qu, B., Zhang, Z., Liang, M., Li, C., Cardona, C.J., Li, D., and Xing, Z. (2014). Evasion of antiviral immunity through sequestering of TBK1/IKKε/IRF3 into viral inclusion bodies. J. Virol. *88*, 3067–3076.

24. Chaudhary, V., Zhang, S., Yuen, K.-S., Li, C., Lui, P.-Y., Fung, S.-Y., Wang, P.-H., Chan, C.-P., Li, D., Kok, K.-H., et al. (2015). Suppression of type I and type III IFN signalling by NSs protein of severe fever with thrombocytopenia syndrome virus through inhibition of STAT1 phosphorylation and activation. J. Gen. Virol. *96*, 3204–3211.

25. Rezelj, V.V., Li, P., Chaudhary, V., Elliott, R.M., Jin, D.-Y., and Brennan, B. (2017). Differential antagonism of human innate immune responses by tick-borne phlebovirus nonstructural proteins. Msphere *2*, e00234-17. https://doi.org/10.1128/msphere.00234-00217.

26. Choi, Y., Park, S.J., Sun, Y., Yoo, J.S., Pudupakam, R.S., Foo, S.S., Shin, W.J., Chen, S.B., Tsichlis, P.N., Lee, W.J., et al. (2019). Severe fever with thrombocytopenia syndrome phlebovirus non-structural protein activates TPL2 signalling pathway for viral immunopathogenesis. Nat. Microbiol. *4*, 429–437. https://doi.org/10.1038/s41564-018-0329-x.

27. Ro, H.J., and Cho, N.H. (2025). Current Advances in Vaccine Development for Severe Fever with Thrombocytopenia Syndrome Virus (SFTSV). Zoonoses (Ireland) *5*, e981. https://doi.org/10.15212/ZOONOSES-2025-0005.

28. Yu, K.M., Park, S.J., Yu, M.A., Kim, Y.I., Choi, Y., Jung, J.U., Brennan, B., and Choi, Y.K. (2019). Cross-genotype protection of live-attenuated vaccine candidate for severe fever with thrombocytopenia syndrome virus in a ferret model. Proc. Natl. Acad. Sci. USA *116*, 26900–26908. https://doi.org/10.1073/pnas.1914704116.

29. Dai, Z.N., Peng, X.F., Li, J.C., Zhao, J., Wu, Y.X., Yang, X., Yang, T., Zhang, S.F., Dai, K., Guan, X.G., et al. (2022). Effect of genomic variations in severe fever with thrombocytopenia syndrome virus on the disease lethality. Emerg. Microbes Infect. *11*, 1672–1682. https://doi.org/10.1080/22221751.2022.2081617.

30. Lee, K., Seok, J.H., Kim, H., Park, S., Lee, S., Bae, J.Y., Jeon, K., Kang, J.G., Yoo, J.R., Heo, S.T., et al. (2023). Genome-informed investigation of the molecular evolution and genetic reassortment of severe fever with thrombocytopenia syndrome virus. Plos Negl. Trop. Dis. *17*, e0011630. https://doi.org/10.1371/journal.pntd.0011630.

31. Brennan, B., Li, P., Zhang, S., Li, A., Liang, M., Li, D., and Elliott, R.M. (2015). Reverse genetics system for severe fever with thrombocytopenia syndrome virus. J. Virol. *89*, 3026–3037. https://doi.org/10.1128/JVI.03432-14.

32. Jeon, K., Ro, H.J., Kang, J.G., Jeong, D.E., Kim, J., Lee, Y., Yoon, G.Y., Kang, J.I., Bae, J.Y., Kim, J.I., et al. (2023). A natural variation in the RNA polymerase of severe fever with thrombocytopenia syndrome virus enhances viral replication and in vivo virulence. J. Med. Virol. *95*, e29099. https://doi.org/10.1002/jmv.29099.

33. Kim, G., Ha, N.Y., Min, C.K., Kim, H.I., Yen, N.T.H., Lee, K.H., Oh, I., Kang, J.S., Choi, M.S., Kim, I.S., and Cho, N.H. (2017). Diversification of Orientia tsutsugamushi genotypes by intragenic recombination and their potential expansion in endemic areas. Plos Negl. Trop. Dis. *11*, e0005408. https://doi.org/10.1371/journal.pntd.0005408.

34. Mosca, J.D., and Pitha, P.M. (1986). Transcriptional and posttranscriptional regulation of exogenous human beta interferon gene in simian cells defective in interferon synthesis. Mol. Cell. Biol. *6*, 2279–2283. https://doi.org/10.1128/mcb.6.6.2279-2283.1986.

35. Wang, T., Xu, L., Zhu, B., Wang, J., and Zheng, X. (2022). Immune escape mechanisms of severe fever with thrombocytopenia syndrome virus. Front. Immunol. *13*, 937684. https://doi.org/10.3389/fimmu.2022.937684.

36. Song, P., Zheng, N., Liu, Y., Tian, C., Wu, X., Ma, X., Chen, D., Zou, X., Wang, G., Wang, H., et al. (2018). Deficient humoral responses and disrupted B-cell immunity are associated with fatal SFTSV infection. Nat. Commun. *9*, 3328. https://doi.org/10.1038/s41467-018-05746-9.

37. Ni, R., Cao, T., Ji, X., Peng, A., Zhang, Z., Fan, G.C., Stathopulos, P., Chakrabarti, S., Su, Z., and Peng, T. (2025). DNA damage-inducible transcript 3 positively regulates RIPK1-mediated necroptosis. Cell Death Differ. *32*, 306–319. https://doi.org/10.1038/s41418-024-01385-4.

38. Yang, C.Y., Lien, C.I., Tseng, Y.C., Tu, Y.F., Kulczyk, A.W., Lu, Y.C., Wang, Y.T., Su, T.W., Hsu, L.C., Lo, Y.C., and Lin, S.C. (2024). Deciphering DED assembly mechanisms in FADD-procaspase-8-cFLIP complexes regulating apoptosis. Nat. Commun. *15*, 3791. https://doi.org/10.1038/s41467-024-47990-2.

39. Verburg, S.G., Lelievre, R.M., Westerveld, M.J., Inkol, J.M., Sun, Y.L., and Workenhe, S.T. (2022). Viral-mediated activation and inhibition of programmed cell death. Plos Pathog. *18*, e1010718. https://doi.org/10.1371/journal.ppat.1010718.

40. Albini, A., Di Paola, L., Mei, G., Baci, D., Fusco, N., Corso, G., and Noonan, D. (2025). Inflammation and cancer cell survival: TRAF2 as a key player. Cell Death Dis. *16*, 292. https://doi.org/10.1038/s41419-025-07609-w.

41. Yao, Y., Zhang, Q., Li, Z., and Zhang, H. (2024). MDM2: current research status and prospects of tumor treatment. Cancer Cell Int. *24*, 170. https://doi.org/10.1186/s12935-024-03356-8.

42. Hu, L., Lu, J., Fan, H., Niu, C., Han, Y., Caiyin, Q., Wu, H., and Qiao, J. (2025). FAS mediates apoptosis, inflammation, and treatment of pathogen infection. Front. Cell. Infect. Microbiol. *15*, 1561102. https://doi.org/10.3389/fcimb.2025.1561102.

43. Zhang, H., Li, X., and Lin, L. (2025). Biological Functions and Clinical Implications of CFLAR: From Cell Death Mechanisms to Therapeutic Targeting in Immune Regulation. J. Inflamm. Res. *18*, 4911–4928. https://doi.org/10.2147/JIR.S519885.

44. Jin, C., Liang, M., Ning, J., Gu, W., Jiang, H., Wu, W., Zhang, F., Li, C., Zhang, Q., Zhu, H., et al. (2012). Pathogenesis of emerging severe fever with thrombocytopenia syndrome virus in C57/BL6 mouse model. Proc. Natl. Acad. Sci. USA *109*, 10053–10058. https://doi.org/10.1073/pnas.1120246109.

45. Kim, Y.M., Ro, H.J., Lee, J.H., Song, Y., Lee, H.W., and Cho, N.H. (2024). Limitations of a proper SFTSV mouse model using human C-type lectin receptors. Front. Microbiol. *15*, 1452739. https://doi.org/10.3389/fmicb.2024.1452739.

46. Kang, J.G., Jeon, K., Choi, H., Kim, Y., Kim, H.I., Ro, H.J., Seo, Y.B., Shin, J., Chung, J., Jeon, Y.K., et al. (2020). Vaccination with single plasmid DNA encoding IL-12 and antigens of severe fever with thrombocytopenia syndrome virus elicits complete protection in IFNAR knockout mice. Plos Negl. Trop. Dis. *14*, e0007813. https://doi.org/10.1371/journal.pntd.0007813.

47. Kim, S., Jeon, K., Choi, H., Jeong, D.E., Kang, J.G., and Cho, N.H. (2024). Comparative analysis of the efficacy of vaccines using structural protein subunits of the severe fever with thrombocytopenia syndrome virus. Front. Microbiol. *15*, 1348276. https://doi.org/10.3389/fmicb.2024.1348276.

48. Kim, H.I., Ha, N.Y., Kim, G., Min, C.K., Kim, Y., Yen, N.T.H., Choi, M.S., and Cho, N.H. (2019). Immunization with a recombinant antigen composed of conserved blocks from TSA56 provides broad genotype protection against scrub typhus. Emerg. Microbes Infect. *8*, 946–958. https://doi.org/10.1080/22221751.2019.1632676.

49. Acosta-Coley, I., Cervantes-Ceballos, L., Tejeda-Benítez, L., Sierra-Márquez, L., Cabarcas-Montalvo, M., García-Espiñeira, M., Coronell-Rodríguez, W., and Arroyo-Salgado, B. (2022). Vaccines platforms and COVID-19: what you need to know. Trop. Dis. Trav. Med. Vaccin. *8*, 20.

50. Kim, D., Lai, C.J., Cha, I., and Jung, J.U. (2024). Current Progress of Severe Fever with Thrombocytopenia Syndrome Virus (SFTSV) Vaccine Development. Viruses *16*, 128. https://doi.org/10.3390/v16010128.

51. Yu, C., Lin, Y., Dai, Y., Wu, B., Qi, Z., and Qian, X. (2024). Recent research advances in the development of Dabie Banda virus vaccines. Plos Negl. Trop. Dis. *18*, e0012411. https://doi.org/10.1371/journal.pntd.0012411.

52. Kim, J.Y., Jeon, K., Park, S.I., Bang, Y.J., Park, H.J., Kwak, H.W., Kim, D.H., Lee, S.Y., Choi, E.J., Cho, N.H., and Nam, J.H. (2023). mRNA vaccine encoding Gn provides protection against severe fever with thrombocytopenia syndrome virus in mice. NPJ Vaccin. *8*, 167. https://doi.org/10.1038/s41541-023-00771-2.

53. Kim, D., Lai, C.J., Cha, I., Kang, S., Yang, W.S., Choi, Y., and Jung, J.U. (2023). SFTSV Gn-Head mRNA vaccine confers efficient protection against lethal viral challenge. J. Med. Virol. *95*, e29203. https://doi.org/10.1002/jmv.29203.

54. Zhang, X., Zhang, X., Lu, J., Yang, P., Lu, M., Tan, S., Guo, W., Hu, X., Lin, J., and Shan, C. (2025). The mRNA vaccine encoding Gc protein confers complete protection against severe fever with thrombocytopaenia syndrome virus. Vaccine *63*, 127631. https://doi.org/10.1016/j.vaccine.2025.127631.

55. Lu, J., Liu, J., Wu, Y., He, X., Gao, X., Chen, X., Chen, S., Zhu, X., Peng, Y., Xiao, G., and Pan, X. (2024). A full-length glycoprotein mRNA vaccine confers complete protection against severe fever with thrombocytopenia syndrome virus, with broad-spectrum protective effects against bandaviruses. J. Virol. *98*, e0076924. https://doi.org/10.1128/jvi.00769-24.

56. Kim, D., Kim, E., Kim, S., Chung, Y., Lai, C.J., Cha, I., Cho, S.D., Choi, Y., Dai, X., Kim, S., et al. (2023). Self-assembling Gn head ferritin nanoparticle vaccine provides full protection from lethal challenge of Dabie bandavirus in aged ferrets. mBio *14*, e0186823. https://doi.org/10.1128/mbio.01868-23.

57. Dong, F., Li, D., Wen, D., Li, S., Zhao, C., Qi, Y., Jangra, R.K., Wu, C., Xia, D., Zhang, X., et al. (2019). Single dose of a rVSV-based vaccine elicits complete protection against severe fever with thrombocytopenia syndrome virus. npj Vaccin. *4*, 5.

58. Yoshikawa, T., Taniguchi, S., Kato, H., Iwata-Yoshikawa, N., Tani, H., Kurosu, T., Fujii, H., Omura, N., Shibamura, M., Watanabe, S., et al. (2021). A highly attenuated vaccinia virus strain LC16m8-based vaccine for severe fever with thrombocytopenia syndrome. Plos Pathog. *17*, e1008859.

59. Hicks, P., Manzoni, T.B., Westover, J.B., Petch, R.J., Roper, B., Gowen, B.B., and Bates, P. (2024). Safety, Immunogenicity, and Efficacy of a Recombinant Vesicular Stomatitis Virus Vectored Vaccine Against Severe Fever with Thrombocytopenia Syndrome Virus and Heartland Bandavirus. Vaccines (Basel) *12*, 1403. https://doi.org/10.3390/vaccines12121403.

60. Tian, L., Yan, L., Zheng, W., Lei, X., Fu, Q., Xue, X., Wang, X., Xia, X., and Zheng, X. (2021). A rabies virus vectored severe fever with thrombocytopenia syndrome (SFTS) bivalent candidate vaccine confers protective immune responses in mice. Vet. Microbiol. *257*, 109076.

61. Zhao, Z., Zheng, W., Yan, L., Sun, P., Xu, T., Zhu, Y., Liu, L., Tian, L., He, H., Wei, Y., and Zheng, X. (2020). Recombinant human adenovirus type 5 co-expressing RABV G and SFTSV Gn induces protective immunity against rabies virus and severe fever with thrombocytopenia syndrome virus in mice. Front. Microbiol. *11*, 1473.

62. Qian, H., Tian, L., Liu, W., Liu, L., Li, M., Zhao, Z., Lei, X., Zheng, W., Zhao, Z., and Zheng, X. (2024). Adenovirus type 5-expressing Gn induces better protective immunity than Gc against SFTSV infection in mice. npj Vaccin. *9*, 194.

63. Kim, J.Y., Jeon, K., Hong, J.J., Park, S.I., Cho, H., Park, H.J., Kwak, H.W., Park, H.J., Bang, Y.J., Lee, Y.S., et al. (2023). Heterologous vaccination utilizing viral vector and protein platforms confers complete protection against SFTSV. Sci. Rep. *13*, 8189. https://doi.org/10.1038/s41598-023-35328-9.

64. Travieso, T., Li, J., Mahesh, S., Mello, J.D.F.R.E., and Blasi, M. (2022). The use of viral vectors in vaccine development. NPJ Vaccin. *7*, 75. https://doi.org/10.1038/s41541-022-00503-y.

65. Heppner, D.G., Kemp, T.L., Martin, B.K., Ramsey, W.J., Nichols, R., Dasen, E.J., Link, C.J., Das, R., Xu, Z.J., Sheldon, E.A., et al. (2017). Safety and immunogenicity of the rVSVΔ G-ZEBOV-GP Ebola virus vaccine candidate in healthy adults: a phase 1b randomised, multicentre, double-blind, placebo-controlled, dose-response study. Lancet Infect. Dis. *17*, 854–866.

66. Henao-Restrepo, A.M., Camacho, A., Longini, I.M., Watson, C.H., Edmunds, W.J., Egger, M., Carroll, M.W., Dean, N.E., Diatta, I., Doumbia, M., et al. (2017). Efficacy and effectiveness of an rVSV-vectored vaccine in preventing Ebola virus disease: final results from the Guinea ring vaccination, open-label, cluster-randomised trial (Ebola Ça Suffit!). The Lancet *389*, 505–518.

67. Henríquez, R., and Muñoz-Barroso, I. (2024). Viral vector-and virus-like particle-based vaccines against infectious diseases: A minireview. Heliyon *10*, e34927.

68. Butt, M., Zaman, M., Ahmad, A., Khan, R., Mallhi, T., Hasan, M., Khan, Y., Hafeez, S., Massoud, E., and Rahman, M.H. (2022). Appraisal for the potential of viral and nonviral vectors in gene therapy: a review. Genes (Basel) *13*, 1370.

69. Manzoni, T.B., Westover, J.B., Lundgreen, K.A., Hicks, P.D., Petch, R.J., Ort, J.T., Weissman, D., Fan, S.H.Y., Hensley, S.E., Pardi, N., et al. (2025). Homologous and Heterologous Vaccination Regimens with mRNA and rVSV Platforms Induce Potent Immune Responses Against SFTSV Glycoprotein. Viruses *17*, 1095. https://doi.org/10.3390/v17081095.

70. Fu, Y., Zhao, J., Han, P., Zhang, J., Wang, Q., Wang, Q., Wei, X., Yang, L., Ren, T., Zhan, S., and Li, L. (2023). Cost-effectiveness of COVID-19 vaccination: A systematic review. J. Evid. Based. Med. *16*, 152–165. https://doi.org/10.1111/jebm.12525.

71. Prosser, L.A., Wallace, M., Rose, A.M., Mercon, K., Janusz, C.B., Gebremariam, A., Hutton, D.W., Leidner, A.J., Zhou, F., Ortega-Sanchez, I.R., et al. (2025). Cost-Effectiveness of 2023-2024 COVID-19 Vaccination in US Adults. JAMA Netw. Open *8*, e2523688. https://doi.org/10.1001/jamanetworkopen.2025.23688.

72. Ruiz, F.J., Torres-Rueda, S., Pearson, C.A.B., Bergren, E., Okeke, C., Procter, S.R., Madriz-Montero, A., Jit, M., Vassall, A., and Uzochukwu, B.S.C. (2023). What, how and who: Cost-effectiveness analyses of COVID-19 vaccination to inform key policies in Nigeria. PLOS Glob. Public Health *3*, e0001693. https://doi.org/10.1371/journal.pgph.0001693.

73. Ra, S.H., Kim, J.Y., Cha, H.H., Kwon, J.S., Lee, H.J., Jeon, N.Y., Kim, M.J., Chong, Y.P., Lee, S.O., Choi, S.H., et al. (2019). Coinfection of Severe Fever with Thrombocytopenia Syndrome and Scrub Typhus in Patients with Tick-Borne Illness. Am. J. Trop. Med. Hyg. *101*, 1259–1262. https://doi.org/10.4269/ajtmh.19-0242.

74. Xu, G., Mendell, N.L., Liang, Y., Shelite, T.R., Goez-Rivillas, Y., Soong, L., Bouyer, D.H., and Walker, D.H. (2017). CD8+ T cells provide immune protection against murine disseminated endotheliotropic Orientia tsutsugamushi infection. Plos Negl. Trop. Dis. *11*, e0005763. https://doi.org/10.1371/journal.pntd.0005763.

75. Hauptmann, M., Kolbaum, J., Lilla, S., Wozniak, D., Gharaibeh, M., Fleischer, B., and Keller, C.A. (2016). Protective and Pathogenic Roles of CD8+ T Lymphocytes in Murine Orientia tsutsugamushi Infection. Plos Negl. Trop. Dis. *10*, e0004991. https://doi.org/10.1371/journal.pntd.0004991.

76. Raskov, H., Orhan, A., Christensen, J.P., and Gögenur, I. (2021). Cytotoxic CD8(+) T cells in cancer and cancer immunotherapy. Br. J. Cancer *124*, 359–367. https://doi.org/10.1038/s41416-020-01048-4.

77. Pai, R.K., Askew, D., Boom, W.H., and Harding, C.V. (2002). Regulation of class II MHC expression in APCs: roles of types I, III, and IV class II transactivator. J. Immunol. *169*, 1326–1333.

78. León Machado, J.A., and Steimle, V. (2021). The MHC class II transactivator CIITA: not (quite) the odd-one-out anymore among NLR proteins. Int. J. Mol. Sci. *22*, 1074.

79. Zuo, J., and Rowe, M. (2012). Herpesviruses placating the unwilling host: manipulation of the MHC class II antigen presentation pathway. Viruses *4*, 1335–1353.

80. Zuo, J., Hislop, A.D., Leung, C.S., Sabbah, S., and Rowe, M. (2013). Kaposi's sarcoma-associated herpesvirus-encoded viral IRF3 modulates major histocompatibility complex class II (MHC-II) antigen presentation through MHC-II transactivator-dependent and-independent mechanisms: implications for oncogenesis. J. Virol. *87*, 5340–5350.

81. Butler, L.M., Jeffery, H.C., Wheat, R.L., Long, H.M., Rae, P.C., Nash, G.B., and Blackbourn, D.J. (2012). Kaposi's sarcoma-associated herpesvirus inhibits expression and function of endothelial cell major histocompatibility complex class II via suppressor of cytokine signaling 3. J. Virol. *86*, 7158–7166.

82. Sun, Q., Jin, C., Zhu, L., Liang, M., Li, C., Cardona, C.J., Li, D., and Xing, Z. (2015). Host responses and regulation by NFκB signaling in the liver and liver epithelial cells infected with a novel tick-borne bunyavirus. Sci. Rep. *5*, 11816.

83. Li, S., Li, H., Zhang, Y.-L., Xin, Q.-L., Guan, Z.-Q., Chen, X., Zhang, X.-A., Li, X.-K., Xiao, G.-F., Lozach, P.-Y., et al. (2020). SFTSV infection induces BAK/BAX-dependent mitochondrial DNA release to trigger NLRP3 inflammasome activation. Cell Rep. *30*, 4370–4385.e7.

84. Du, C., Fang, M., Li, Y., Li, L., and Wang, X. (2000). Smac, a mitochondrial protein that promotes cytochrome c–dependent caspase activation by eliminating IAP inhibition. Cell *102*, 33–42.

85. Szegezdi, E., Logue, S.E., Gorman, A.M., and Samali, A. (2006). Mediators of endoplasmic reticulum stress-induced apoptosis. EMBO Rep. *7*, 880–885.

86. Czabotar, P.E., Lessene, G., Strasser, A., and Adams, J.M. (2014). Control of apoptosis by the BCL-2 protein family: implications for physiology and therapy. Nat. Rev. Mol. Cell Biol. *15*, 49–63.

87. Yonekawa, H., and Akita, Y. (2008). Protein kinase Cepsilon: the mitochondria-mediated signaling pathway. Febs J. *275*, 4005–4013. https://doi.org/10.1111/j.1742-4658.2008.06558.x.

88. Centonze, M., Saponaro, C., and Mangia, A. (2018). NHERF1 Between Promises and Hopes: Overview on Cancer and Prospective Openings. Transl. Oncol. *11*, 374–390. https://doi.org/10.1016/j.tranon.2018.01.006.

89. Luo, Z., Tian, M., Yang, G., Tan, Q., Chen, Y., Li, G., Zhang, Q., Li, Y., Wan, P., and Wu, J. (2022). Hypoxia signaling in human health and diseases: implications and prospects for therapeutics. Signal Transduct. Target. Ther. *7*, 218. https://doi.org/10.1038/s41392-022-01080-1.

90. Lin, S., and Zhang, Y.-J. (2017). Interference of apoptosis by hepatitis B virus. Viruses *9*, 230.

91. Vinogradskaya, G.R., Ivanov, A.V., and Kushch, A.A. (2022). Mechanisms of Survival of Cytomegalovirus-Infected Tumor Cells. Mol. Biol. *56*, 668–683.

92. Guo, H., Koehler, H.S., Dix, R.D., and Mocarski, E.S. (2022). Programmed cell death-dependent host defense in ocular herpes simplex virus infection. Front. Microbiol. *13*, 869064.

93. Li, S., Li, H., Zhou, Z., Ye, M., Wang, Y., Li, W., Guan, Z., Guan, Z., Zhang, C., Zhang, Y., et al. (2025). A viral necrosome mediates direct RIPK3 activation to promote inflammatory necroptosis. Proc. Natl. Acad. Sci. USA *122*, e2420245122. https://doi.org/10.1073/pnas.2420245122.

94. Cook, I.F. (2021). Subcutaneous vaccine administration - an outmoded practice. Hum. Vaccin. Immunother. *17*, 1329–1341. https://doi.org/10.1080/21645515.2020.1814094.

95. Matsuno, K., Orba, Y., Maede-White, K., Scott, D., Feldmann, F., Liang, M., and Ebihara, H. (2017). Animal Models of Emerging Tick-Borne Phleboviruses: Determining Target Cells in a Lethal Model of SFTSV Infection. Front. Microbiol. *8*, 104. https://doi.org/10.3389/fmicb.2017.00104.

96. Brennan, B., Rezelj, V.V., and Elliott, R.M. (2017). Mapping of transcription termination within the S segment of SFTS phlebovirus facilitated generation of NSs deletant viruses. J. Virol. *91*, e00743-17. https://doi.org/10.1128/jvi.00743-00717.

97. Ha, N.-Y., Cho, N.-H., Kim, Y.-S., Choi, M.-S., and Kim, I.-S. (2011). An autotransporter protein from Orientia tsutsugamushi mediates adherence to nonphagocytic host cells. Infect. Immun. *79*, 1718–1727.

98. Min, C.K., Kim, H.I., Ha, N.Y., Kim, Y., Kwon, E.K., Yen, N.T.H., Youn, J.I., Jeon, Y.K., Inn, K.S., Choi, M.S., and Cho, N.H. (2018). A Type I Interferon and IL-10 Induced by Orientia tsutsugamushi Infection Suppresses Antigen-Specific T Cells and Their Memory Responses. Front. Immunol. *9*, 2022. https://doi.org/10.3389/fimmu.2018.02022.

99. Frey, A., Di Canzio, J., and Zurakowski, D. (1998). A statistically defined endpoint titer determination method for immunoassays. J. Immunol. Methods *221*, 35–41.

YMTHE, Volume *34*

Supplemental Information

Recombinant *Dabie bandavirus* as a bivalent vaccine platform inducing protective immunity against intracellular pathogens and cancer

Hyo-Jin Ro, Yebeen Lee, Kyeongseok Jeon, Yujin Kim, Seung Ho Baek, Green Kim, Joowan Kim, Jun-Gu Kang, Na-Yoon Jang, Si-Hyeon Lee, Sun-Young Kim, Yu-Jin Kim, Na-Young Ha, Yuri Kim, Young Ki Choi, Jae U. Jung, Jung Joo Hong, and Nam-Hyuk Cho

Table S1. Serum biochemistry in WT mice following intramuscular inoculation with SFTSV-ΔNSs or mock control

Sex	Male						Female					
D.P.I.[a]	2		17		31		2		17		31	
Group (n)	Mock (5)	SFTSV-ΔNSs (5)	Mock (9)	SFTSV-ΔNSs (10)	Mock (5)	SFTSV-ΔNSs (5)	Mock (5)	SFTSV-ΔNSs (5)	Mock (9)	SFTSV-ΔNSs (10)	Mock (5)	SFTSV-ΔNSs (5)
ALT (U/L)	33.7±17.0	26.6±6.5	29.0±5.8	27.4±4.4	28.0±7.5	30.7±3.6	32.0±16.1	27.4±6.0	30.3±5.6	31.7±9.7	44.2±16.0	40.7±14.4
AST (U/L)	63.2±22.2	54.4±12.8	56.6±11.4	60.1±26.0	45.4±4.5	46.7±8.4	55.4±16.5	54.6±9.5	81.2±19.0	94.5±33.4	129.3±24.8	185.7±49.1
ALP (U/L)	535.4±72.3	528.6±50.6	392.9±43.6	387.2±40.9	336.5±30.0	316.4±21.6	540.2±83.8	584.1±93.2	590.0±60.7	492.8±59.6**	425.7±22.8	419.1±40.7
GGT (U/L)	0.02±0.03	0.17±0.29	0.59±0.25	0.55±0.33	0.00±0.00	0.00±0.00	0.23±0.27	0.26±0.19	0.15±0.22	0.08±0.12	0.00±0.00	0.02±0.05
BUN (mg/dL)	23.4±2.8	20.7±4.4	27.4±2.9	24.6±3.8	24.0±1.7	25.1±1.4	19.2±2.0	19.8±1.7	24.6±3.3	27.4±2.9	28.5±2.9	27.2±2.0
Urea (mg/dL)	50±6	44±9	59±6	53±8	51±4	54±3	41±4	42±4	53±7	59±6	61±6	59±4
Crea (mg/dL)	0.36±0.02	0.33±0.02*	0.39±0.03	0.39±0.04	0.39±0.05	0.39±0.03	0.35±0.03	0.29±0.03*	0.31±0.03	0.32±0.05	0.36±0.07	0.37±0.05

[a]D.P.I: days post-inoculation. *, $p<0.05$ by Mann Whitney test.

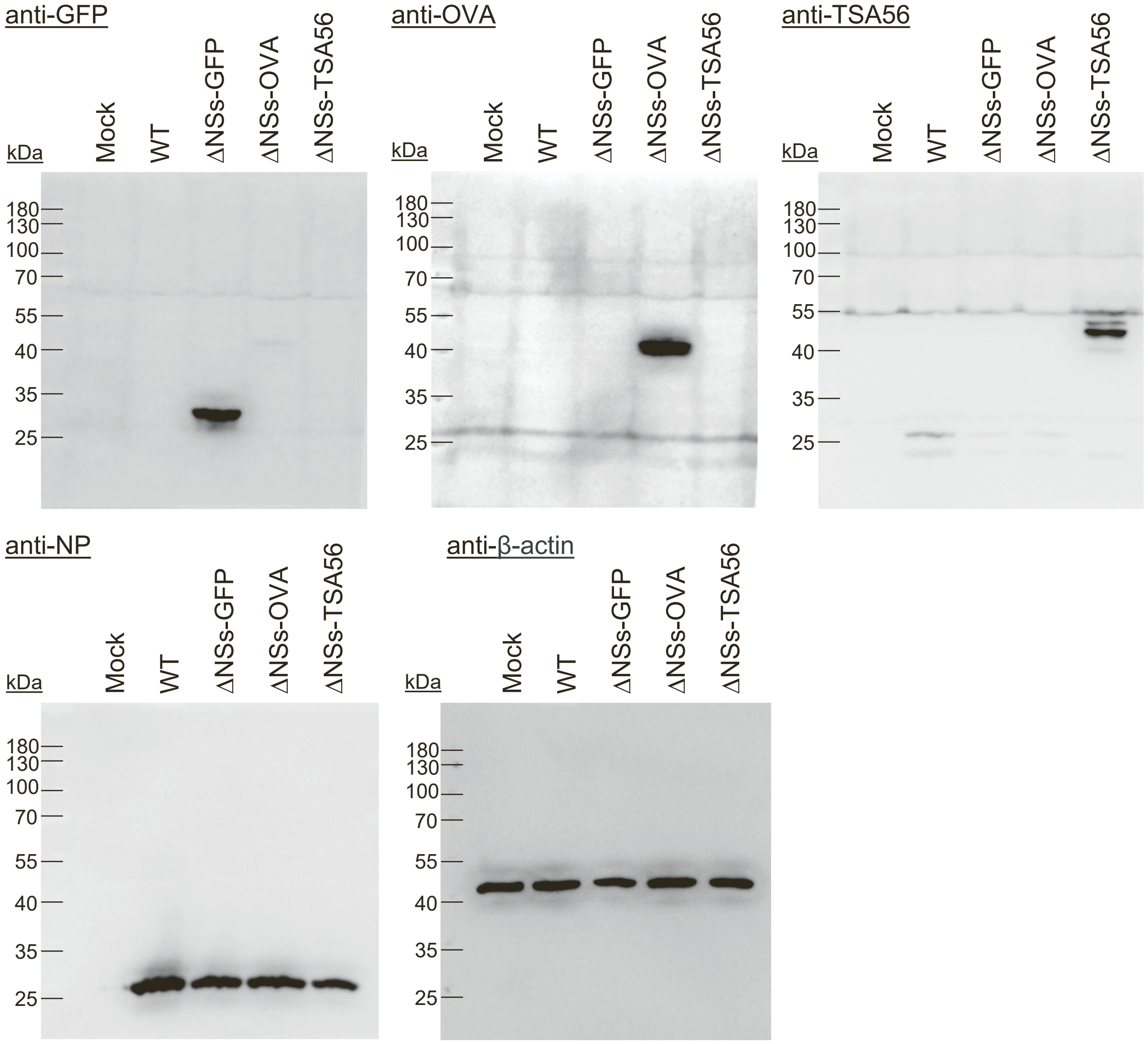

Figure S1. Immunoblot analysis of Vero E6 cells infected with SFTSV ΔNSs vectors expressing GFP, OVA, or TSA56 for 48 hours. Whole-membrane blots probed with the indicated antibodies are shown.

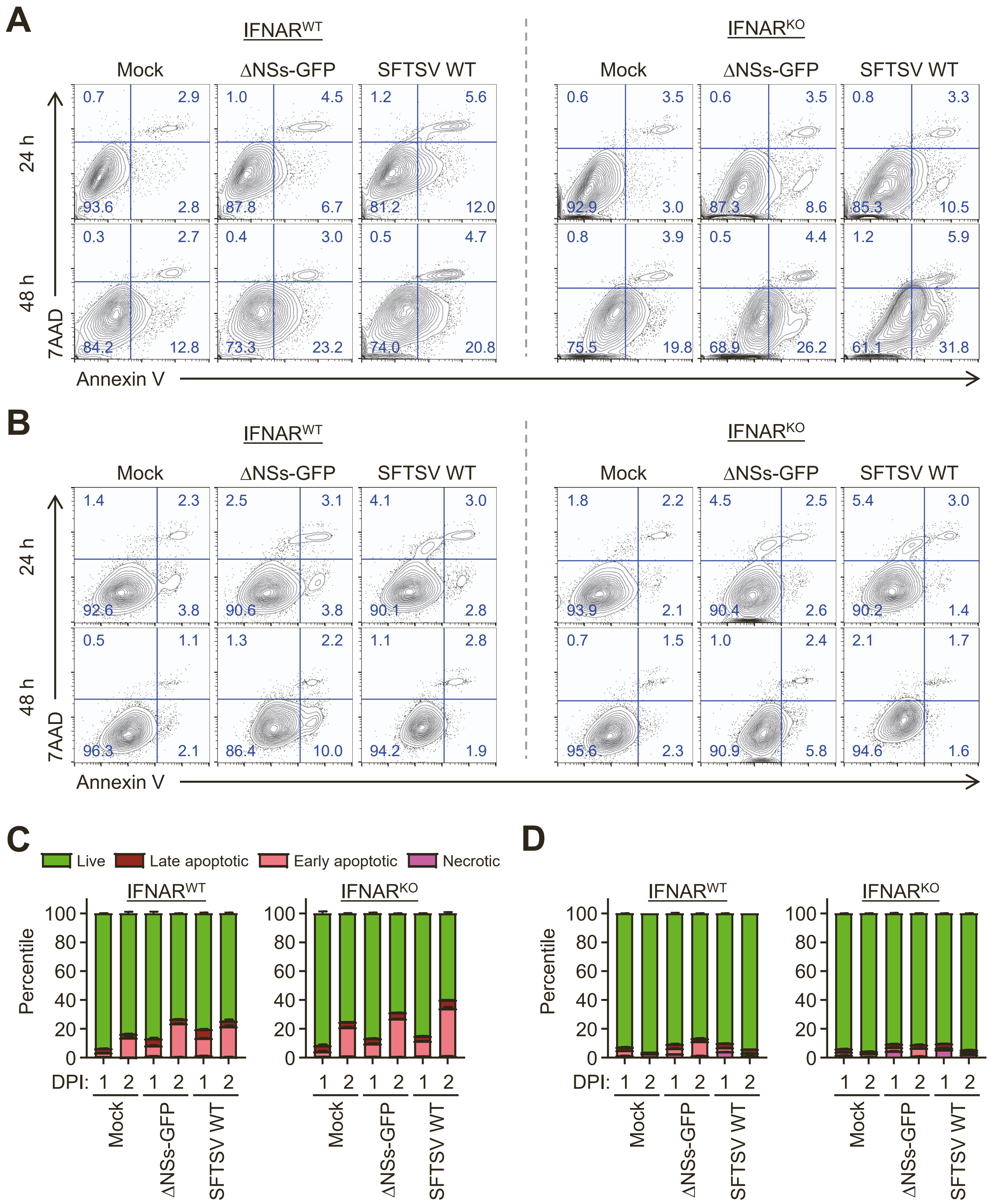

Figure S2. Apoptotic and necrotic cell death in SFTSV-infected APCs. BMDCs and BMDMs from WT or IFNAR KO mice were infected with SFTSV ΔNSs-GFP at an MOI of 1. Apoptotic and necrotic cell populations were analyzed by flow cytometry at 24 and 48 hours post-infection. **(A, B)** Representative flow cytometry plots showing Annexin V and 7-AAD staining in BMDCs **(A)** and BMDMs **(B)**. **(C, D)** Quantification of apoptotic and necrotic cell fractions in BMDCs **(C)** and BMDMs **(D)**. Cells were categorized as live (Annexin V$^-$ 7-AAD$^-$), late apoptotic (Annexin V$^+$ 7-AAD$^+$), early apoptotic (Annexin V$^+$ 7-AAD$^-$), necrotic (Annexin V$^-$ 7-AAD$^+$).

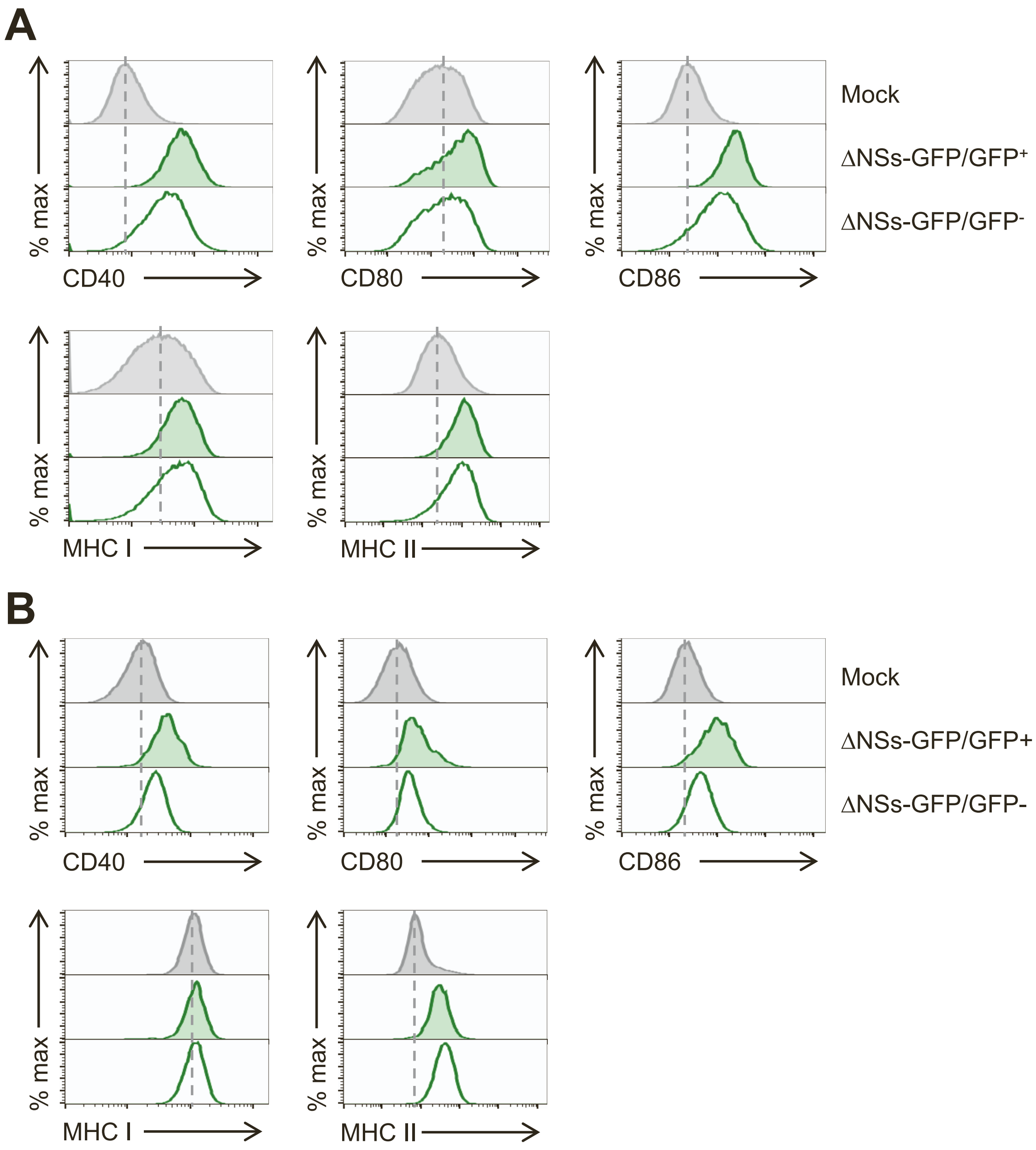

Figure S3. Histogram of co-stimulatory and MHC expression in GFP$^+$ and GFP$^-$ APCs. (A, B) WT BMDCs **(A)** and BMDMs **(B)** were infected with SFTSV ΔNSs-GFP at an MOI of 1. Representative histograms show the expression profiles of co-stimulatory molecules (CD40, CD80, CD86) and MHC class I and II in GFP$^+$ (infected) versus GFP$^-$ (uninfected) cell populations.

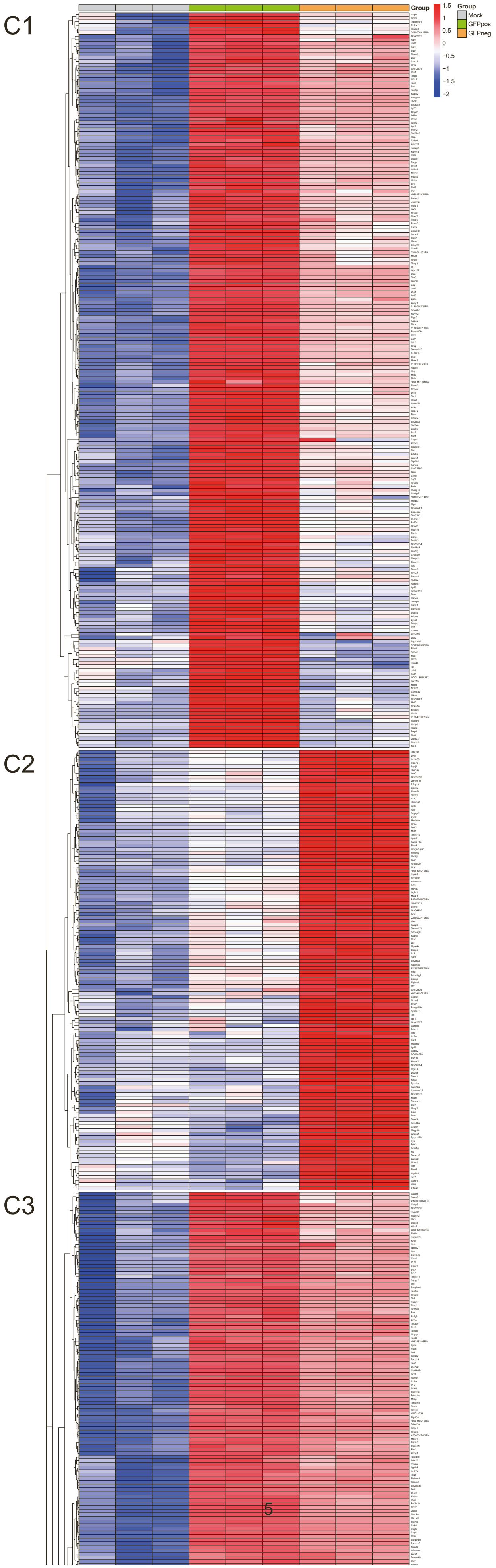
C1
C2
C3
Group
1.5
1
0.5
0
−0.5
−1
−1.5
−2
Group
Mock
GFPpos
GFPneg

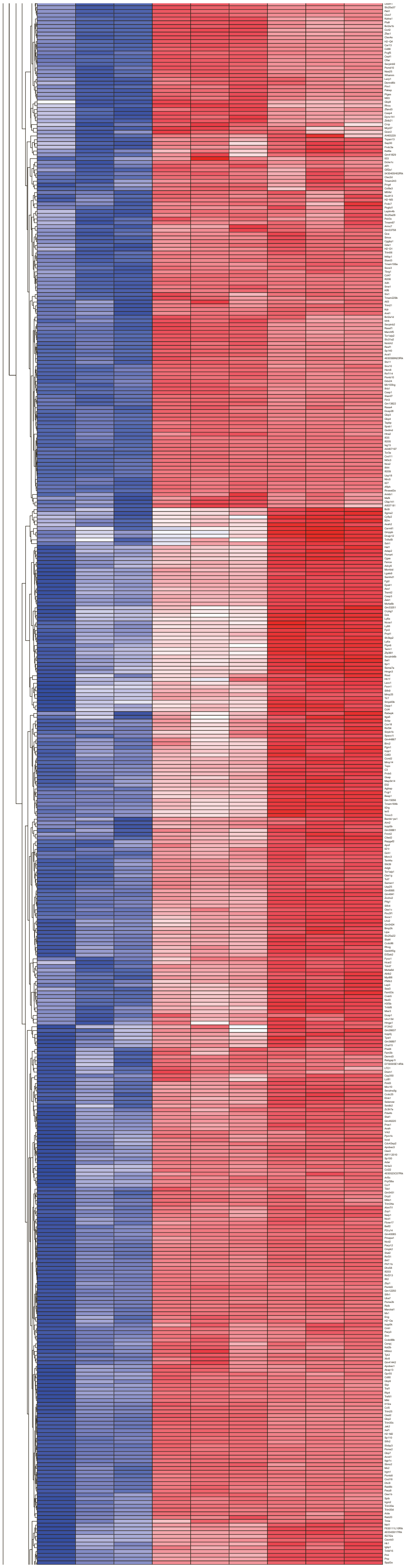

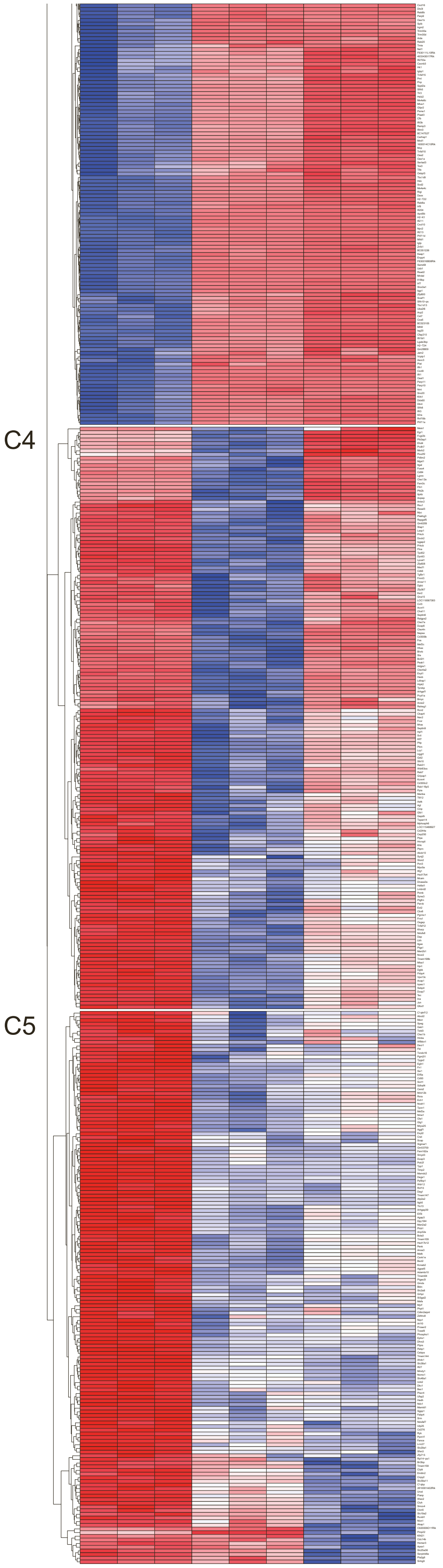

Figure S4. Heatmap of differentially expressed genes (DEGs) across groups. Genes with |Log2 (fold change, FC)| > 1 and FDR < 0.05 (relative to mock) were clustered into five modules (C1–C5) by unsupervised hierarchical clustering.

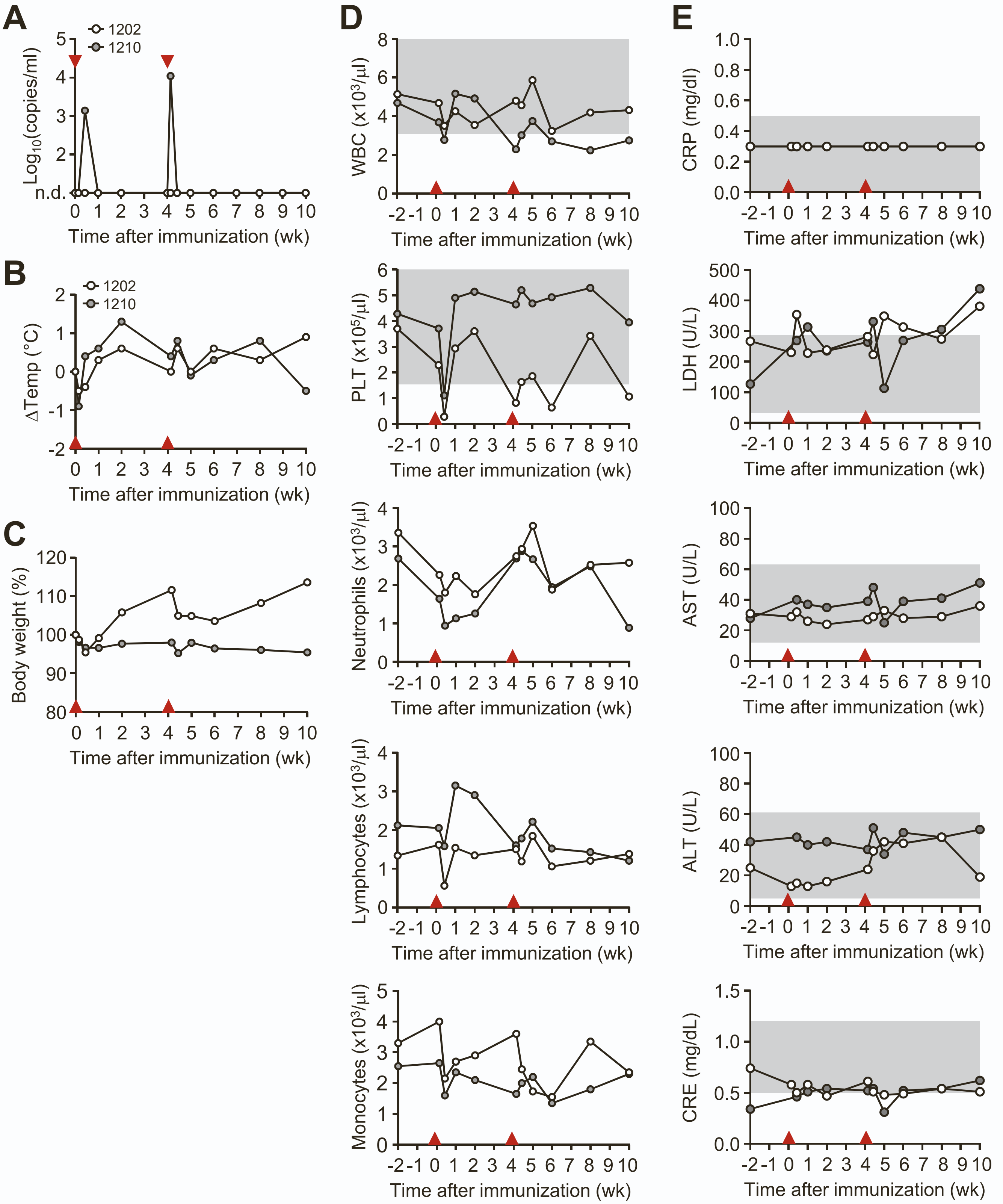

Figure S5. Hematological and biochemical monitoring following SFTSV ΔNS immunization in Rhesus macaques. Rhesus macaques were immunized twice with 10^6 FFU of SFTSV ΔNS at four-week intervals (i.m.). Red arrows indicate immunization time points. Body weight, temperature, viremia, complete blood count (CBC), and serum biochemistry were monitored over 10 weeks. **(A–C)** Viral RNA levels in serum **(A)**, body temperature **(B)**, and body weight **(C)** were measured at indicated time points. **(D)** Hematological parameters included WBC, platelet (PLT) count, neutrophils, lymphocytes, and monocytes. Gray shading indicates normal reference ranges for Rhesus macaques. **(E)** Biochemical markers included C-reactive protein (CRP), lactate dehydrogenase (LDH), AST, ALT, and creatinine (CRE).

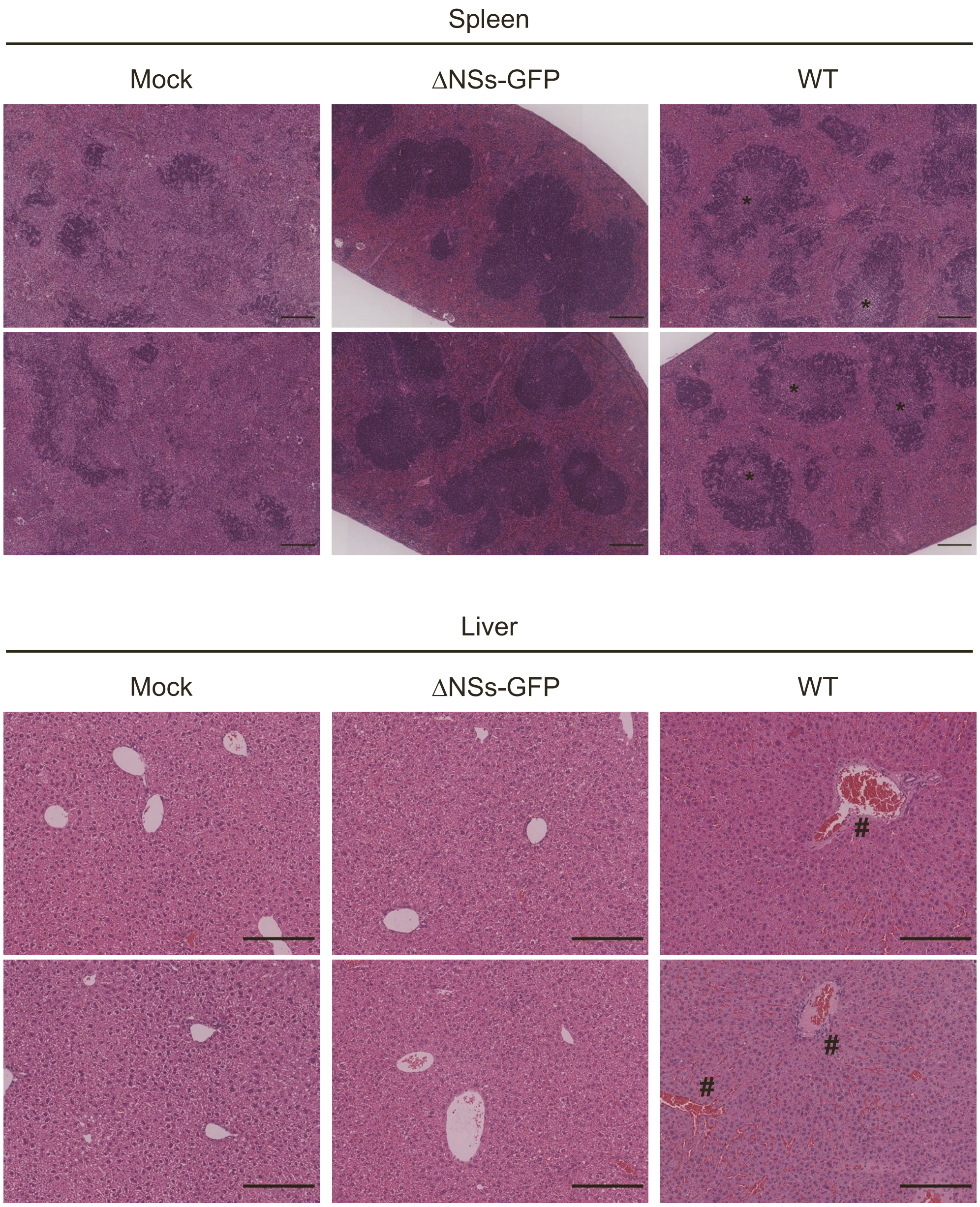

Figure S6. Histopathological analysis of IFNAR KO mice following infection with SFTSV. IFNAR KO mice were subcutaneously inoculated with 10^3 FFU of WT or ΔNSs-GFP SFTSV and sacrificed at day 3 post-infection. Representative hematoxylin and eosin (H&E)-stained spleen (left) and liver (right)sections. White pulp atrophy (*) in spleen and vascular congestion (#) in liver from mice infected with WT SFTSV are indicated. Scale bars, 200 µm.

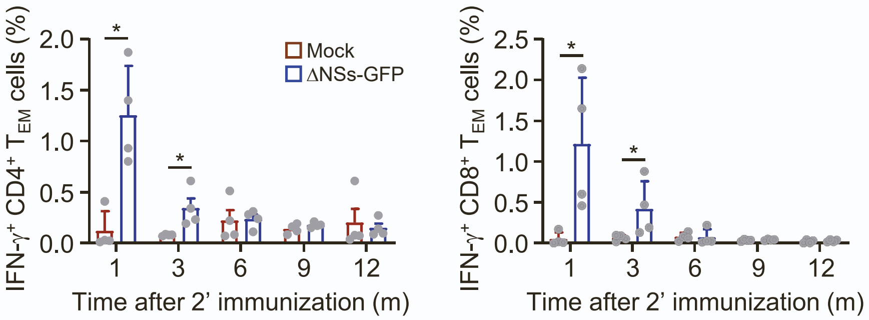

Figure S7. Immunization of recombinant SFTSV ΔNSs induces durable cellular immunity in IFNAR KO mice up to 3 months post-immunization. IFN-γ-producing CD4$^+$ and CD8$^+$ T cells were assessed by flow cytometry following NP stimulation of splenocytes collected at indicated time post-boost immunization (n = 4/group). *, $p < 0.05$ by Mann Whitney test.

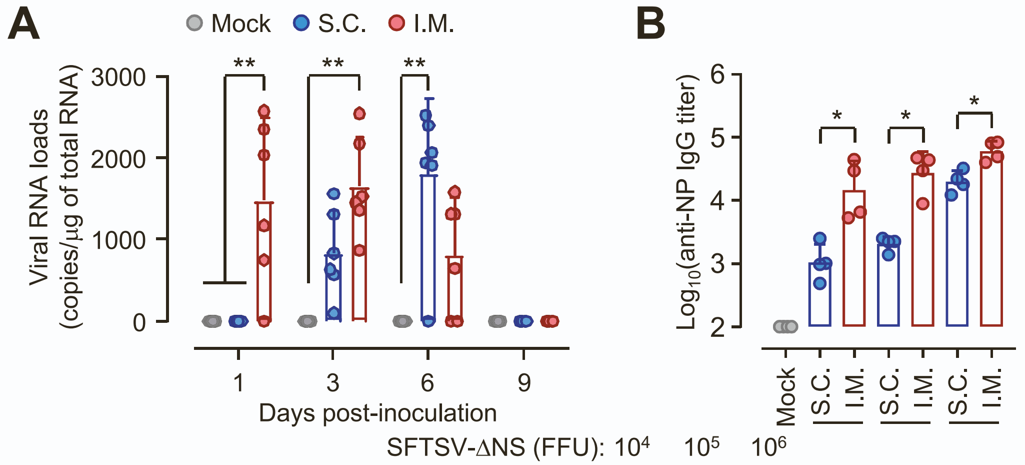

Figure S8. Differential viral dissemination and antibody responses according to inoculation route of SFTSV-ΔNSs in WT mice. (A) Viral RNA levels in draining (inguinal) lymph nodes at the indicated days post-inoculation (10^6 FFFU/mouse). **(B)** Anti-NP IgG responses measured two weeks after viral inoculation via the indicated routes and doses. S.C., subcutaneously; I.M., intramuscularly. *, $p < 0.05$; **, $p < 0.01$ by Kruskal Wallis test or Mann Whitney test.

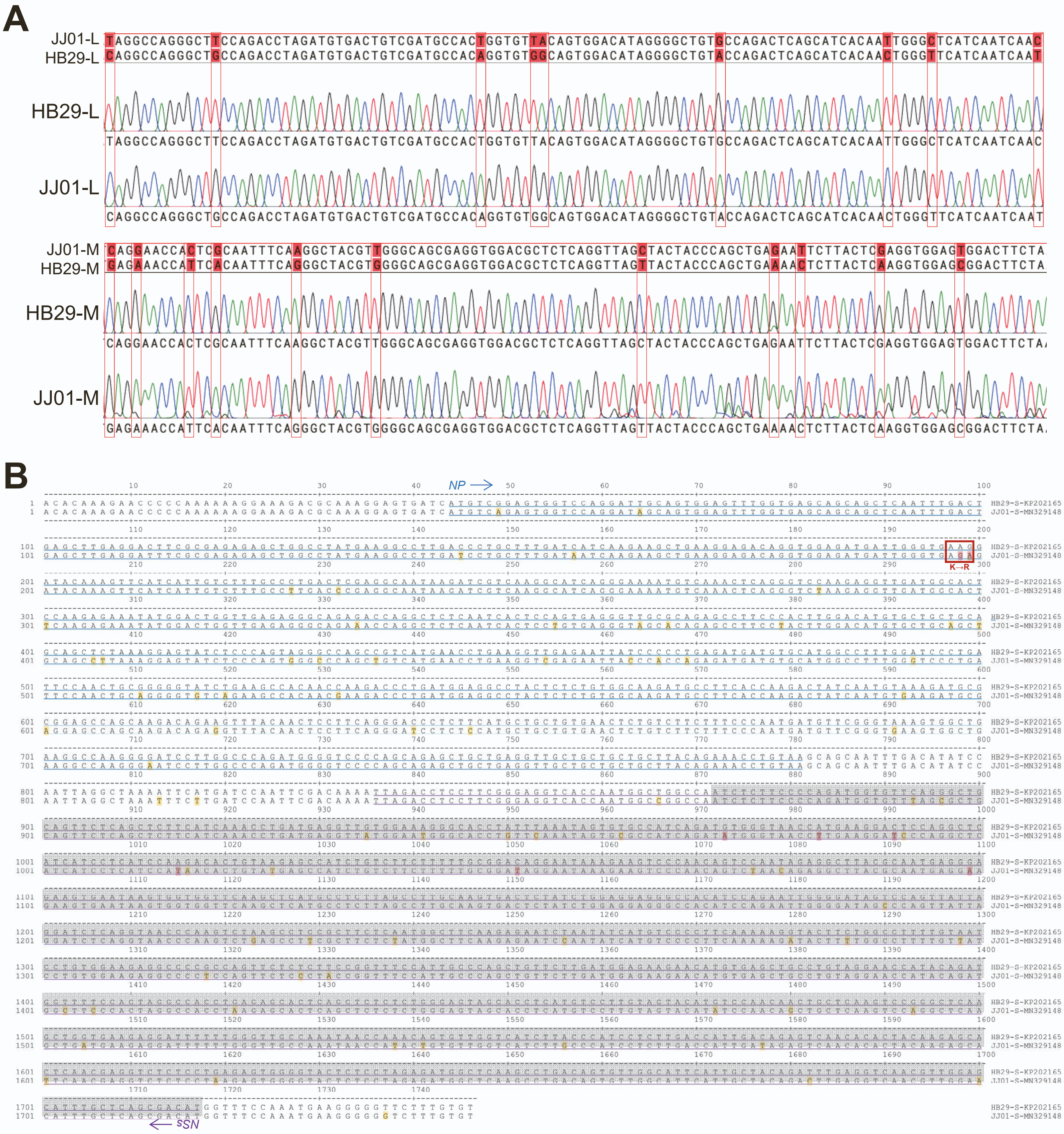

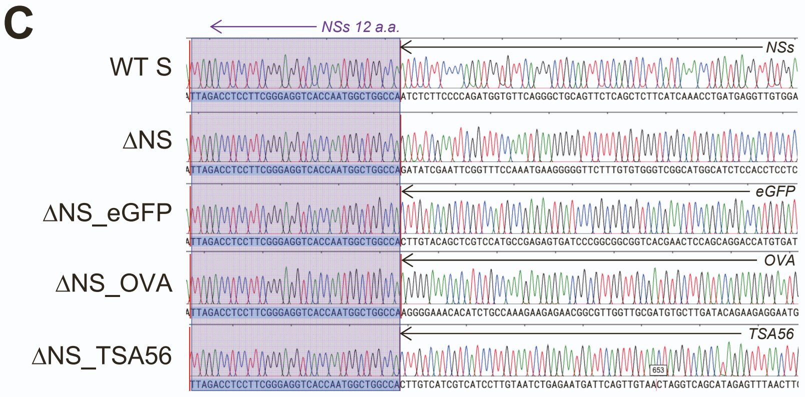

Figure S9. Representative sequencing results of isolated reassortant viral clones. (A) Sanger sequencing of the L and M segments, with sequence comparisons between HB29 and JJ strains. **(B)** Whole S segment sequence comparison between HB29 and JJ strains. A nonsynonymous mutation at position K53R in the *NP* gene is highlighted by a red box. **(C)** Sanger sequencing of the NSs-deleted region within the S segment of the indicated recombinant SFTSV isolates.

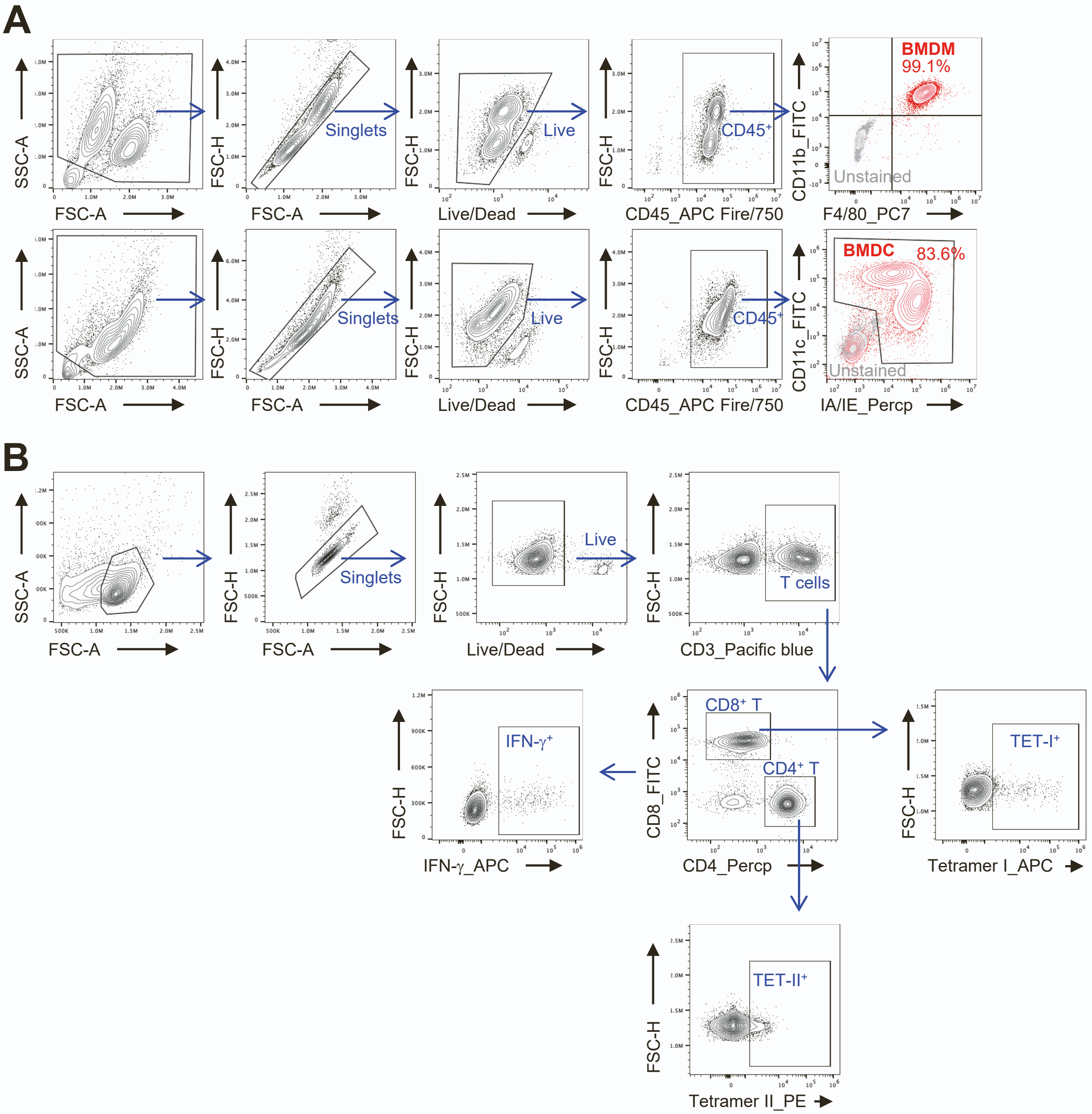

Figure S10. Representative flow cytometry gating strategies for APC and antigen-specific T cell identification. (A) Gating strategies for identifying BMDMs and BMDCs. BMDMs were defined as CD11b^{+} F4/80^{+} cells and BMDCs as CD11c^{+} IA/IE^{+} cells. **(B)** Antigen-specific T cells were gated on CD3^{+} populations, with further identification of CD4^{+} and CD8^{+} subsets. Intracellular IFN-γ^{+} cells, as well as OVA-specific tetramer I^{+} and tetramer II^{+} cells, were analyzed using appropriate isotype and negative controls.
